# Genome-Wide Analyses Reveal a Role for Peptide Hormones in Planarian Germline Development

**DOI:** 10.1371/journal.pbio.1000509

**Published:** 2010-10-12

**Authors:** James J. Collins, Xiaowen Hou, Elena V. Romanova, Bramwell G. Lambrus, Claire M. Miller, Amir Saberi, Jonathan V. Sweedler, Phillip A. Newmark

**Affiliations:** 1 Howard Hughes Medical Institute and Department of Cell and Developmental Biology, University of Illinois at Urbana-Champaign, Urbana, Illinois, United States of America; 2 Neuroscience Program, University of Illinois at Urbana-Champaign, Urbana, Illinois, United States of America; 3 Center for Biophysics and Computational Biology, University of Illinois at Urbana-Champaign, Urbana, Illinois, United States of America; 4 Department of Chemistry, University of Illinois at Urbana-Champaign, Urbana, Illinois, United States of America; UCLA, United States of America

## Abstract

Bioactive peptides (i.e., neuropeptides or peptide hormones) represent the largest class of cell-cell signaling molecules in metazoans and are potent regulators of neural and physiological function. In vertebrates, peptide hormones play an integral role in endocrine signaling between the brain and the gonads that controls reproductive development, yet few of these molecules have been shown to influence reproductive development in invertebrates. Here, we define a role for peptide hormones in controlling reproductive physiology of the model flatworm, the planarian *Schmidtea mediterranea*. Based on our observation that defective neuropeptide processing results in defects in reproductive system development, we employed peptidomic and functional genomic approaches to characterize the planarian peptide hormone complement, identifying 51 prohormone genes and validating 142 peptides biochemically. Comprehensive in situ hybridization analyses of prohormone gene expression revealed the unanticipated complexity of the flatworm nervous system and identified a prohormone specifically expressed in the nervous system of sexually reproducing planarians. We show that this member of the neuropeptide Y superfamily is required for the maintenance of mature reproductive organs and differentiated germ cells in the testes. Additionally, comparative analyses of our biochemically validated prohormones with the genomes of the parasitic flatworms *Schistosoma mansoni* and *Schistosoma japonicum* identified new schistosome prohormones and validated half of all predicted peptide-encoding genes in these parasites. These studies describe the peptide hormone complement of a flatworm on a genome-wide scale and reveal a previously uncharacterized role for peptide hormones in flatworm reproduction. Furthermore, they suggest new opportunities for using planarians as free-living models for understanding the reproductive biology of flatworm parasites.

## Abstract 2

Genomic/peptidomic analyses of the planarian *Schmidtea mediterranea* identifies >200 neuropeptides and uncovers a conserved neuropeptide required for proper maturation and maintenance of the reproductive system.

## Author Summary

Flatworms cause diseases affecting hundreds of millions of people, so understanding what influences their reproductive activity is of fundamental importance. Neurally derived signals have been suggested to coordinate sexual reproduction in free-living flatworms, yet the neuroendocrine signaling repertoire has not been characterized comprehensively for any flatworm. Neuropeptides are a large diverse group of cell-cell signaling molecules and play many roles in vertebrate reproductive development; however, little is known about their function in reproductive development among invertebrates. Here we use biochemical and bioinformatic techniques to identify bioactive peptides in the genome of the planarian flatworm *Schmidtea mediterranea* and identify 51 genes encoding >200 peptides. Analysis of these genes in both sexual and asexual strains of *S. mediterranea* identified a neuropeptide Y superfamily member as important for the normal development and maintenance of the planarian reproductive system. We suggest that understanding peptide hormone function in planarian reproduction could have practical implications in the treatment of parasitic flatworms.

## Introduction

Platyhelminthes (flatworms) inhabit a variety of aquatic and terrestrial environments and members of the phylum are thought to parasitize most vertebrate species [1]. The remarkable ability of flatworms to maintain plasticity in their reproductive cycles is a key to their success. As an example, free-living planarian flatworms are capable of reproducing sexually as cross-fertilizing hermaphrodites or asexually by transverse fission [2]. Some planarian species even maintain the ability to switch between modes of sexual and asexual reproduction, resorbing and regenerating their reproductive organs, depending on the environmental context [3]. This dynamic regulation of reproductive development is not limited to free-living platyhelminths; parasitic flatworms can also undergo dramatic changes in their reproductive development in response to external stimuli. In dioecious parasites of the genus *Schistosoma*, female reproductive development requires pairing with a male worm [4]–[8]. Thus, female schistosomes derived from single-sex infections have underdeveloped ovaries and accessory reproductive organs when compared to females from mixed sex infections. Interestingly, the reproductive organs of mature females deprived of their male counterpart regress and are capable of regrowing once male-female pairing is reestablished [9]. Because flatworms, including schistosomes, are responsible for causing important neglected tropical diseases, understanding the mechanisms that coordinate the reproduction of both free-living and parasitic members of the phylum is of fundamental importance.

Peptide hormones (i.e. neuropeptides) are among the most structurally and functionally diverse class of metazoan signaling molecules [10]. In vertebrates, a neuroendocrine axis involving peptide hormone signaling between the brain and the gonads controls the maturation and long-term maintenance of reproductive development and function [10]–[13]. A similar role for neuroendocrine signals in controlling flatworm reproduction is suggested by studies exploiting the well-known regeneration abilities of planarians. Head amputation (i.e. removal of the brain/cephalic ganglia) of sexually reproducing planarians results in regression of the testes [14],[15] to their germ cell primordia [16], which re-grow only when cephalic ganglia regeneration is complete. These observations suggest that neural signals control the dynamics of planarian reproduction. Thus, flatworms may employ peptide-based mechanisms, similar to vertebrates, to synchronize their reproductive development.

To date only limited data exist to support a “vertebrate-like” role for peptide hormones in invertebrate reproductive maturation. Insulin-like peptides influence germline stem cell proliferation in *Drosophila* [17],[18] and *C. elegans* [19] and promote oocyte maturation in the starfish *Asterina pectinifera* [20] and the mosquito *Aedes aegypti* [21]. In locusts, treatment with the peptide hormones ovary maturing parsin (OVP) [22] or short Neuropeptide F (sNPF) [23],[24] can stimulate ovarian development and vitellogenesis. Because of this paucity of data linking neuroendocrine function to invertebrate reproductive development, additional studies are required to determine how invertebrates modulate their reproductive output in response to external and metabolic cues.

Peptide hormones are processed proteolytically from longer secretory prohormone precursors and often require covalent modifications before becoming biologically active [10],[25]. As a result of this extensive processing, and because the biologically relevant signaling units are encoded by short stretches of amino acid sequence (usually 3–40 amino acids), predicting genes encoding these molecules represents a major challenge for bioinformatics-driven genome annotation. The recent application of bioinformatic approaches coupled to mass spectrometry-based peptide characterization techniques (an approach called peptidomics [26]–[28]) has revolutionized discovery efforts, uncovering hundreds of new genes encoding metazoan bioactive peptides. Among invertebrates, however, much of this recent progress has been focused on genome-wide studies of nematodes [29]–[31], arthropods [32]–[36], and mollusks [37],[38]. Thus, little is known of the peptide hormones present in phyla such as Platyhelminthes. Despite recent bioinformatic efforts to characterize flatworm peptide-encoding genes [39],[40], only three distinct peptides have been characterized extensively at the biochemical level in all flatworms [41].

Owing to a wealth of functional genomic tools [42] and a sequenced genome [43], the planarian *S. mediterranea* represents an ideal model to characterize flatworm neuropeptides. Furthermore, this species exists as two distinct strains: an asexual strain that lacks reproductive organs and propagates exclusively by fission and a sexual strain that reproduces as cross-fertilizing hermaphrodites [44]. This dichotomy presents a unique opportunity to explore the extent to which peptide hormones are associated with distinct reproductive states. To address the possibility that peptide signals influence planarian reproductive development, we began by disrupting a gene encoding a prohormone processing enzyme, *Smed-prohormone convertase 2* (*Smed-pc2*, GB: BK007043), in sexual planarians. Consistent with a role for peptide hormones in controlling planarian reproduction, knockdown of *Smed-pc2* led to a depletion of differentiated germ cells in the planarian testes. To identify potential peptide mediators of this effect, we used peptidomic approaches to characterize the peptide hormone complement of *S. mediterranea*. This analysis identified 51 genes predicted to encode more than 200 peptides, 142 of which we characterized biochemically by mass spectrometry. Global analysis of the expression of these genes by whole mount in situ hybridization revealed a distinct distribution of some peptide prohormones between sexual and asexual strains of *S. mediterranea*. We find one prohormone gene, *npy-8*, to be enriched in the nervous system of sexual planarians and show that this gene is required for the proper development and maintenance of reproductive tissues. These results demonstrate the utility of *S. mediterranea* as a model to characterize metazoan peptides and suggest that flatworm reproductive development is controlled by neuroendocrine signals.

## Results

### A Peptide Hormone-Processing Enzyme Is Required for the Maintenance of Differentiated Germ Cells

**Figure 1:**
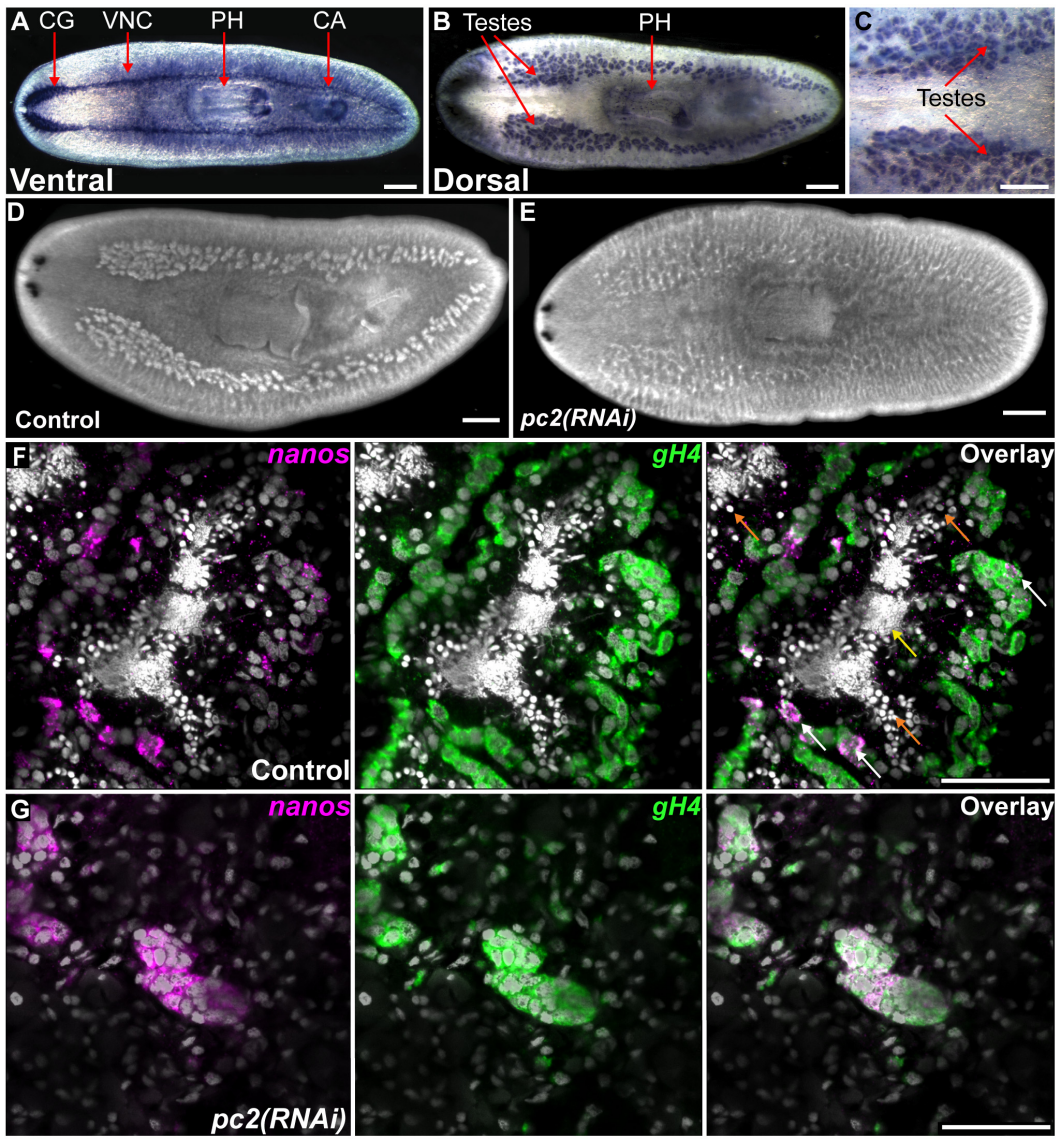
*pc2* is essential for the maintenance of the planarian testes. (A–C) Whole-mount in situ hybridization to detect *pc2* mRNA in sexual animals. (A) Ventral view, expression in CNS, pharynx, and copulatory apparatus. (B and C) Dorsal view, expression in testes. (D, E) DAPI staining showing the distribution of testes in (D) control and (E) *pc2(RNAi*) animals fixed 17 d after the initiation of RNAi treatment. (F and G) Single confocal sections showing expression of *nanos* (magenta) and *gH4* (green) in testes of (F) control and (G) *pc2(RNAi*) animals. DAPI staining is shown in grey. Orange and yellow arrows indicate spermatids and mature sperm, respectively. White arrows indicate germ line stem cells expressing both *gH4* and *nanos*. Scale bars: (A–C) 300 µm; (D and E) 500 µm; (F and G) 50 µm. Abbreviations: CG, Cephalic Ganglia; VNC, ventral nerve cord; PH, pharynx; CA, copulatory apparatus.

To explore potential roles for peptide signaling in regulating planarian reproductive physiology, we characterized *Smed-pc2* (Figure S1), whose orthologues are required in both vertebrate and invertebrate models for the proteolytic processing of prohormones to mature neuropeptides (in the interest of brevity, we will drop the prefix “*Smed*” from the remainder of the genes described below) [30],[45],[46]. A large-scale RNA interference (RNAi) screen determined that this gene was essential for coordinated movement and normal regeneration in asexual planarians [47]. Whole-mount in situ hybridization in sexual planarians revealed expression of *pc2* in the central nervous system [48], the pharynx, sub-muscular cells, the photoreceptors, the copulatory apparatus, and the testes (Figure 1A–C).

To determine if peptide signals are likely to play a functional role in coordinating reproductive development, we monitored the effects of *pc2* RNAi on the dynamics of germ cells within the planarian testes. Individual testis lobes consist of an outer spermatogonial layer in which cells divide to form cysts of eight spermatocytes that, after meiosis, give rise to spermatids and, ultimately, sperm [44],[49]. After 17 d of RNAi treatment, *pc2(RNAi)* animals displayed a decrease in both testis size (Figure 1E) and the number of animals producing mature sperm (28/29 for controls versus 2/36 for *pc2* RNAi; *p*<0.0001, Student's *t* test). To establish which cell types are affected by *pc2* RNAi, we performed fluorescence in situ hybridization (FISH) to detect *germinal histone H4* (*gH4*) (GB: DN306099) and *nanos* (GB: EF035555) mRNAs, which are expressed in spermatogonia and germline stem cells (GSCs), respectively [16],[50],[51]. In developed testes of control animals, relatively few cells within the outer spermatogonial layer are identifiable as *nanos*-positive GSCs (Figure 1F). However in *pc2(RNAi)* animals, regressed testes clusters almost always co-expressed both *gH4* and *nanos* (Figure 1G) (*n* = 16/17 animals). These results suggest that *pc2* is required for proper germ cell differentiation and/or for the maintenance of differentiated germ cells in the testes.

### Genomic Identification of Peptide Hormones and Their Encoded Peptides

**Figure 2:**
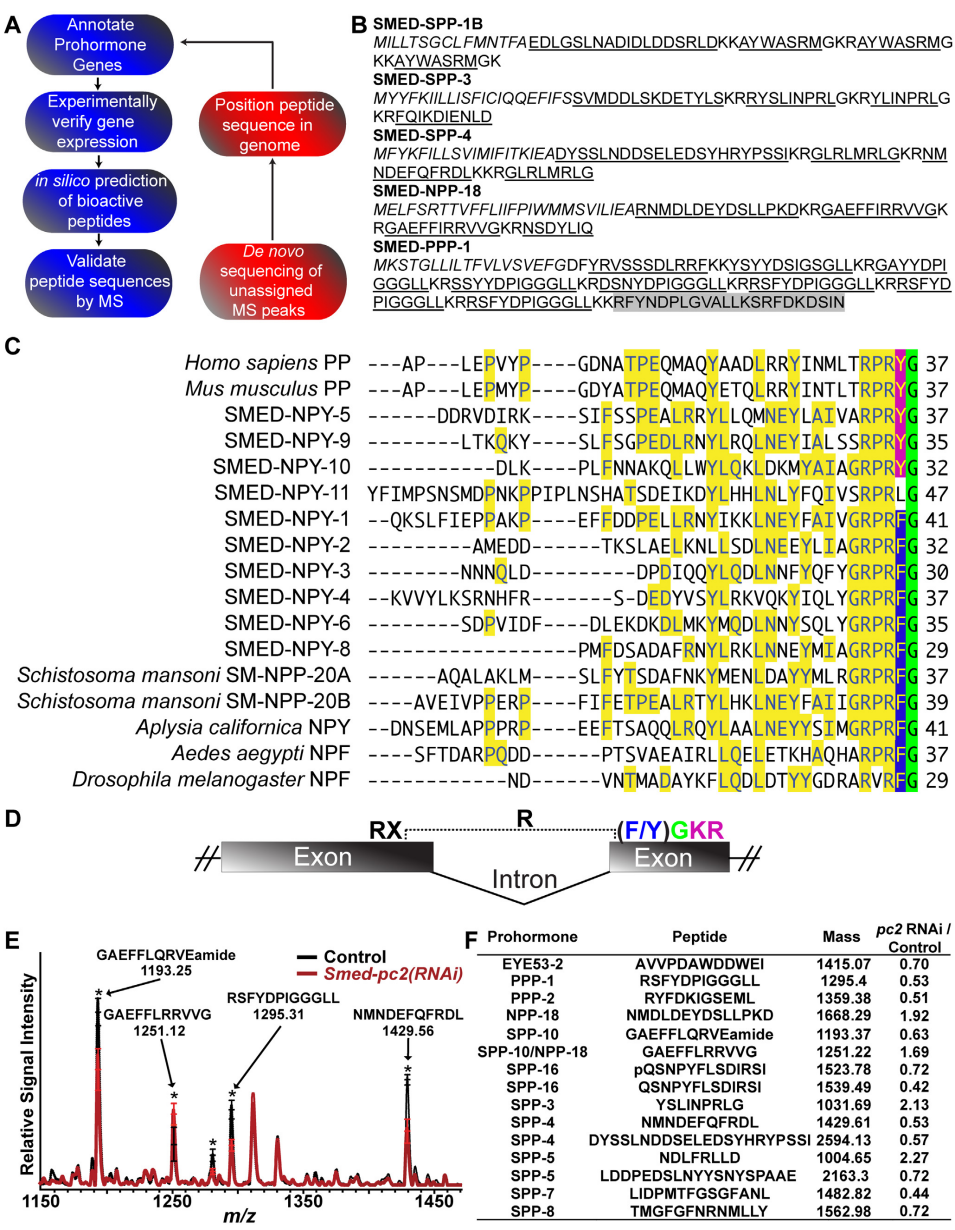
Overview of the peptidomic approach to characterize peptide-encoding genes from *S. mediterranea*. (A) Schematic representation of the methodology used for the identification and confirmation of planarian prohormones and their respective peptides. We performed homology and pattern searches for preliminary annotation of peptide prohormone genes and subsequently verified these predictions with molecular techniques. The post-translational processing of verified prohormones to bioactive peptides was then predicted in silico using Neuropred [52] and the sequences of mature peptides were then confirmed in whole animal tissue extracts from sexual and asexual planarians by LC-MS/MS and/or MALDI-TOF MS. This approach is depicted in blue ovals. To complement the bioinformatics-driven discovery, de novo sequencing of unassigned MS peaks was used to characterize novel neuropeptides (red ovals). The sequences of such peptides were then mapped to the *S. mediterranea* genome and new prohormone genes were annotated. These prohormone genes were then analyzed further, leading to the characterization of additional peptides. (B) Full sequence coverage of prohormones SPP-1B, SPP-3, SPP-4, NPP-18, and PPP-1 by mass spectrometry. Underlined sequences indicate peptides identified by MS/MS sequencing and the shaded sequence indicates a peptide detected by MS mass match. Signal peptides for each prohormone are italicized. (C and D) *S. mediterranea* possesses an expanded NPY family. (C) ClustalW alignment of two vertebrate NPY-family peptides, Pancreatic Polypeptide (PP), with a variety of invertebrate NPY family members. Matching residues are shown in yellow and a conserved α-amidation site is shown in green. C-terminal tyrosine and phenylalanine are highlighted in magenta and blue, respectively. (D) Gene structure of vertebrate and *S. mediterranea npy* genes. These prohormone genes have an intron within the arginine codon preceding the aromatic amino acid residue (blue), the α-amidation site (green), and the dibasic cleavage site (magenta). *npy-11* lacks a C-terminal aromatic residue but also shares this gene organization. (E and F) MALDI-MS analysis of *pc2* RNAi in sexual animals. (E) Comparison of peptide profiles for control and *pc2(RNAi)*-treated sexual animals 16 d after the initiation of RNAi treatment. MALDI-TOF MS spectra (limited to m/z 1150–1450) comparing control and *pc2(RNAi)* groups (*n* = 7 for each group); stars indicate peaks that were significantly different (*p*<0.05). (F) Characterized peptides and their respective prohormones that were detected at significantly different levels (*p*<0.05) following *pc2* RNAi. The *pc2* RNAi/control column reports the ratio of peak intensities of *pc2* RNAi relative to control.

Since our analysis of *pc2* implicated peptide signaling in regulating planarian reproductive development, we characterized the peptide hormone complement of *S. mediterranea*. We employed bioinformatic and mass spectrometry (MS)-based methodologies to identify peptide prohormone genes from the *S. mediterranea* genome [43] and predict their processing into bioactive peptides (Figure 2A) [52]. With these approaches, we identified 51 prohormone genes in *S. mediterranea*, with peptides from 40 prohormones detected by MS (Tables S1–S5, gene names and abbreviations are shown in Table 1). In most cases, MS confirmed multiple distinct peptides from a single prohormone, and in five prohormones we detected every predicted peptide by MS (Figure 2B). In total, we characterized 142 peptides biochemically, corresponding to ∼45% of the distinct peptides predicted from our collection of 51 prohormone genes (Table S5). This analysis identified genes encoding relatives of all previously characterized flatworm neuropeptides (YIRFamide [53], *spp-11*; FRFamide [54], *npp-4*; and neuropeptide Y-like [55], *npy-1* to *npy-11*) and provided biochemical validation for 10 prohormones previously predicted from the *S. mediterranea* genome [39].

**Table 1:** Abbreviations of gene names for *S. mediterranea* neuropeptide prohormones.

Gene Name	Abbreviation	Gene Subfamily
*cerebral peptide prohormone-1*	*cpp-1*	—
*gonadotropin releasing hormone like-1*	*grh-1*	—
*insulin-like peptide-1*	*Ilp-1*	—
*myomodulin prohormone-like-1,2*	*mpl-1* *,*2*	—
*neuropeptide precursor-1-5,8,12,18,22*	*npp-1-5* *,*8* *, *12* *,*18* *,*22* *	—
*neuropeptide y superfamily-1-11*	*npy-1* *	neuropeptide F
	*npy-2*	neuropeptide F
	*npy-3*	neuropeptide F
	*npy-4* *	neuropeptide F
	*npy-5*	neuropeptide Y
	*npy-6*	neuropeptide F
	*npy-7*	neuropeptide Y
	*npy-8*	neuropeptide F
	*npy-9* *	neuropeptide Y
	*npy-10*	neuropeptide Y
	*npy-11*	atypical neuropeptide Y
*pyrokinin prohormone like-1*	*ppl-1*	—
*pedal peptide prohormone* like-1,2	*ppp-1,2*	—
*secreted peptide prohormone-1-19*	*spp-1-19*	—

*Genes previously predicted from the *S. mediterranea* genome [39].

The neuropeptide Y (NPY)-superfamily represents a large family of neuropeptides that influence diverse processes in both vertebrate and invertebrate taxa [10],[41],[56]. This family is considered to consist of two types of peptides: the NPY-like peptides that possess a C-terminal amidated tyrosine (Y) residue and the NPF peptides that possess a C-terminal amidated phenylalanine (F) residue [55]. Vertebrate genomes typically encode NPY-like peptides [57], whereas invertebrate genomes encode NPF peptides [55],[58],[59]. Our studies found that the planarian genome possesses an expanded family of *npy* genes predicted to encode both NPY-like and NPF-like peptides (Figure 2C). Prohormones NPY-5, -7, -9, and -10 possess a C-terminal tyrosine residue, similar to vertebrate NPY peptides, and prohormones SMED-NPY-1, -2, -3, -4, -6, and -8 contain a C-terminal phenylalanine residue, similar to invertebrate NPF peptides. Three of these planarian *npy* genes (*npy-1*, *-4*, and *-9*) have been described previously [39],[60]. Additionally, our studies, and those of others [39],[61], find evidence of conservation in the genomic organization of flatworm NPY genes. NPY genes from vertebrates possess an intron that separates the exon encoding the RXR motif from the penultimate amidated amino acid residue (Figure 2D) [62]. We found an identical architecture for *S. mediterranea* genes *npy-1*, *-2*, *-3*, *-4*, *-5*, *-6*, -*8*, *-9*, *-10*, and *-11*, indicating a close evolutionary relationship between chordate and platyhelminth *npy* genes (Figure 2C,D).

The planarian genome also encodes peptides with sequence similarities to those from other invertebrate taxa, including mollusks (*ppp-1*, GB:BK007041; *ppp-2*, GB:BK007018; *mpl-1*, GB: BK007017; *mpl-2*, GB: BK007016; and, *cpp-1*, GB: BK007012) and arthropods (*ppl-1*, GB: BK007007). Furthermore, our analysis found that previously characterized, novel planarian genes encode peptide prohormones. Homologues of prohormones *eye53-1,2* (GB: BK007033 and GB: BK007024, respectively) and *1020-1,2* (GB: GU295180 and GB:BK007025, respectively) from the planarian *Dugesia japonica* are required for proper visual system function following amputation; knockdown animals show no morphological defects after injury yet are unable to respond properly to light [63]. These previous observations, together with our findings that these genes encode neuropeptides, suggest a role for peptide signaling in the functional recovery of the planarian nervous system following injury.

### *pc2* Is Required for Proper Prohormone Processing

To examine if *pc2* is required for prohormone processing in planarians, we disrupted *pc2* expression using RNAi and performed MS to analyze the peptide complement of *pc2(RNAi)* animals. Consistent with *pc2* encoding a genuine prohormone convertase, analysis of peptide profiles in planarian tissue extracts by MALDI-TOF MS analysis demonstrated that *pc2* RNAi resulted in a significant decrease in the signal intensity of a specific set of peptides in sexual animals (Figure 2E,F and Table S6). Interestingly, the levels of some peptides were increased following *pc2(RNAi)*; whether this alteration reflects a compensatory mechanism for regulating peptide levels or an altered threshold of detection for certain peptides caused by a global reduction in neuropeptide levels remains to be determined. However, these data parallel studies of *pc2* knockout mice, in which the abundance of some peptides was either increased or decreased [45]. Given that the *S. mediterranea* genome is predicted to encode at least three additional proteins with similarity to prohormone convertases (Figure S2), it is possible that compensatory mechanisms are responsible for the observed elevation in the levels of some peptides. This redundancy among prohormone convertases is also likely to explain why we only observed changes in a subset of peptides following *pc2* RNAi. These data suggest that the reproductive defects observed in *pc2(RNAi)* animals may be due to altered levels of specific peptides.

### In Situ Hybridization Analyses Reveal the Complexity of the Flatworm Nervous System

**Figure 3:**
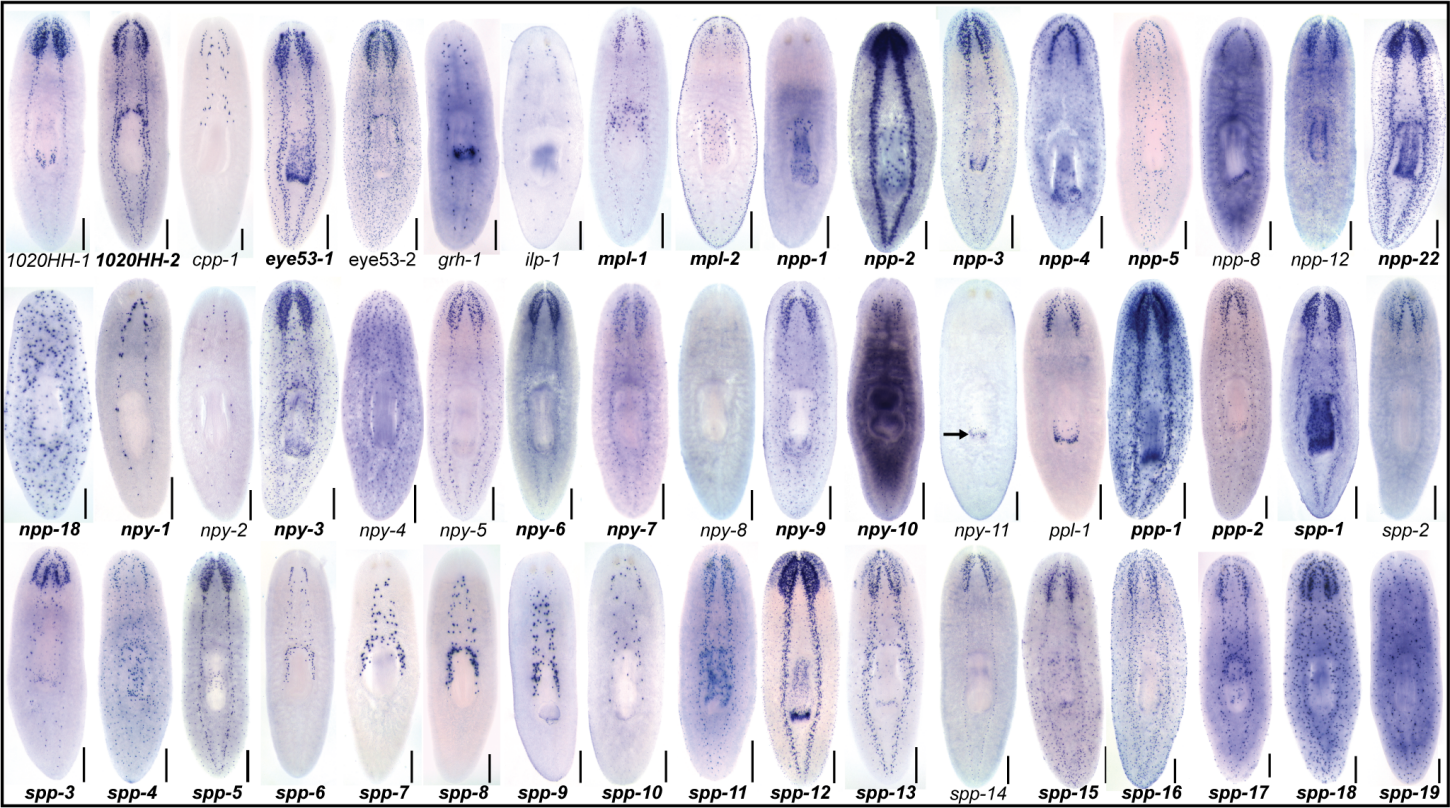
Whole-mount in situ hybridization to detect neuropeptide prohormone gene expression in asexual planarians. Prohormone genes are displayed alphabetically. Full gene names are provided in Table 1. No expression was detected for *npy-8* in asexual animals. Arrow for *npy-11* indicates expression at the distal region of the pharynx. Gene names in bold indicate prohormones with at least one peptide confirmed by MS analysis. Ventral views, anterior towards top. Scale bars (to right of images), 300 µm.

To determine the extent to which peptides may regulate flatworm reproduction, we took advantage of the fact that *S. mediterranea* exists as both sexually and asexually reproducing strains. By comparing prohormone gene expression between these strains we sought to uncover expression patterns specific to sexually or asexually reproducing animals. Thus, we began by performing comprehensive whole-mount in situ hybridization analyses of prohormone genes in asexual planarians (Figure 3).

Our studies indicate that in asexual planarians ∼85% (44/51) of prohormone genes are expressed in the central nervous system (CNS) (Table S5), which consists of bi-lobed cephalic ganglia and two ventral nerve cords (VNCs) that run the length of the body [64]. Of the prohormones expressed in the CNS, 20% (10/51) were detected only in the cephalic ganglia. Notably, the expression of individual prohormones was often enriched in specific cell types or regions within the CNS. For example, the expression of some prohormones was enriched in either lateral (e.g. *npp-4*, GB: BK007037; *npp-8*, GB: GU295189; *spp-4*, GB: GU295179; *and 1020HH-2*), medial (e.g. *spp-2*, GB: BK007032; and *spp-6*, GB: GU295177) or posterior (e.g. *npy-1*, GB: GU295175) regions of the cephalic ganglia (Figure 3). Strikingly, a large fraction of prohormone mRNAs were detected in restricted cell populations within the CNS (e.g. *npy-1*; *npy-2*, GB: BK007019; *cpp-1*; *spp-6*; *spp-9*, GB: BK007026; *spp-10*, GB: BK007028; *grh-1*, GB: GU295185; and *ilp-1*, GB: BK007034) (Figure 3).

Consistent with peptide signaling having a role in processes outside the CNS, we also detected prohormone expression in: the pharynx (e.g. *npp-1*, GB: BK007036; *npp-22*, GB: BK007038; *npy-11*, BK007021; and *ppp-1*); photoreceptors (e.g. *eye53-1,-2*; *npp-12*, GB: GU295182; *and mpl-2*); sub-epidermal marginal adhesive glands (e.g. *mpl-2*); an anterior domain between the VNCs (e.g. *spp-6*; *spp-7*, GB: GU295178; *spp-8*, GB: GU295181; *spp-9*; *cpp-1*; and *spp-10*, GB: BK007028); cells surrounding the ventral midline (e.g. *npp-5*, BK007015); the intestine (e.g. *npp-8*, GB: GU295189; and *npy-10*, GB: BK007011); and various sub-epidermal cell types (e.g. *npp-18*, GB: BK007027; *spp-4*; *spp-16*, GB: BK007042; *and npy-4*, BK007039) (Figure 3).

**Figure 4:**
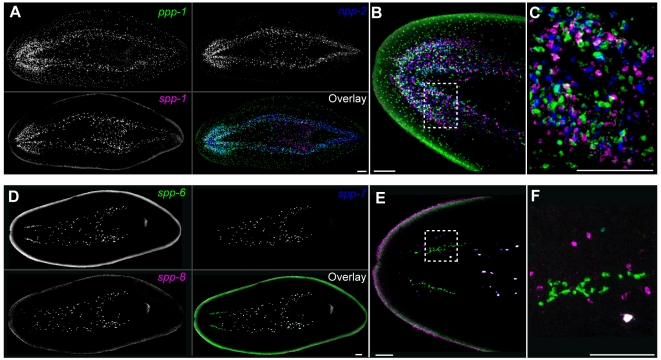
Prohormone gene expression reveals morphological complexity of the planarian nervous system. (A) Three-color FISH for *ppp-1*, *npp-2*, and *spp-1*. (B and C) Merged images, colors indicated in panel A. Prohormone genes *ppp-1*, *npp-2*, and *spp-1* are not predicted to encode any related peptides and do not appear to have overlapping distributions within the CNS. (D) Three-color FISH for prohormone genes *spp-6*, *spp-7*, and *spp-9*. (E and F) Merged images, colors indicated in panel D. Prohormones genes *spp-6*, *spp-7*, and *spp-9* encode related prohormones that are co-expressed in cells between the VNCs; the expression of these genes is not co-localized in the CNS. Images from (A–F) are confocal projections; whole animal views in (A) and (D) are derived from tiled stacks. Ventral views, anterior towards left. Scale bars, 100 µm.

To investigate the extent to which prohormones are expressed in overlapping or distinct cell types in the CNS, we compared the expression of prohormone genes using triple FISH. Prohormone genes *spp-1* (GB: GU295176), *npp-2* (GB: BK007035), and *ppp-1* encode unrelated peptides (Tables S1 and S5) that appear to be expressed ubiquitously in the CNS (Figure 3). Comparison of the expression domains of these prohormone genes revealed that *spp*-1, *npp-2*, and *ppp-1* are expressed in largely non-overlapping populations of cells of the cephalic ganglia and VNCs (Figure 4A–C). We also analyzed the expression of a family of paralogous prohormone genes (*spp-6*; *spp-7*; *spp-8*; *spp-9*; and *spp-17*, GB: GU295183) that encode similar neuropeptides (Figure S3). Because this gene family has been expanded in the *S. mediterranea* genome, we refer to these prohormones as the Planarins. Examination of Planarins *spp-6*, -*7*, and -*9* expression by FISH demonstrated that these genes are expressed in a common set of cells distributed between the VNCs and surrounding the pharynx (Figure 4D). Despite being co-expressed in cells outside the CNS, *spp-6* and *spp-9* transcripts were detected in distinct groups of cells within the cephalic ganglia (Figure 4E,F). These findings, with earlier observations [48],[64], suggest a level of complexity not previously appreciated for the patterning of the flatworm nervous system (see Figure S4).

### Prohormone Expression Identifies Anterior-Posterior and Dorsal-Ventral Compartments Within the Planarian Photoreceptors

**Figure 5:**
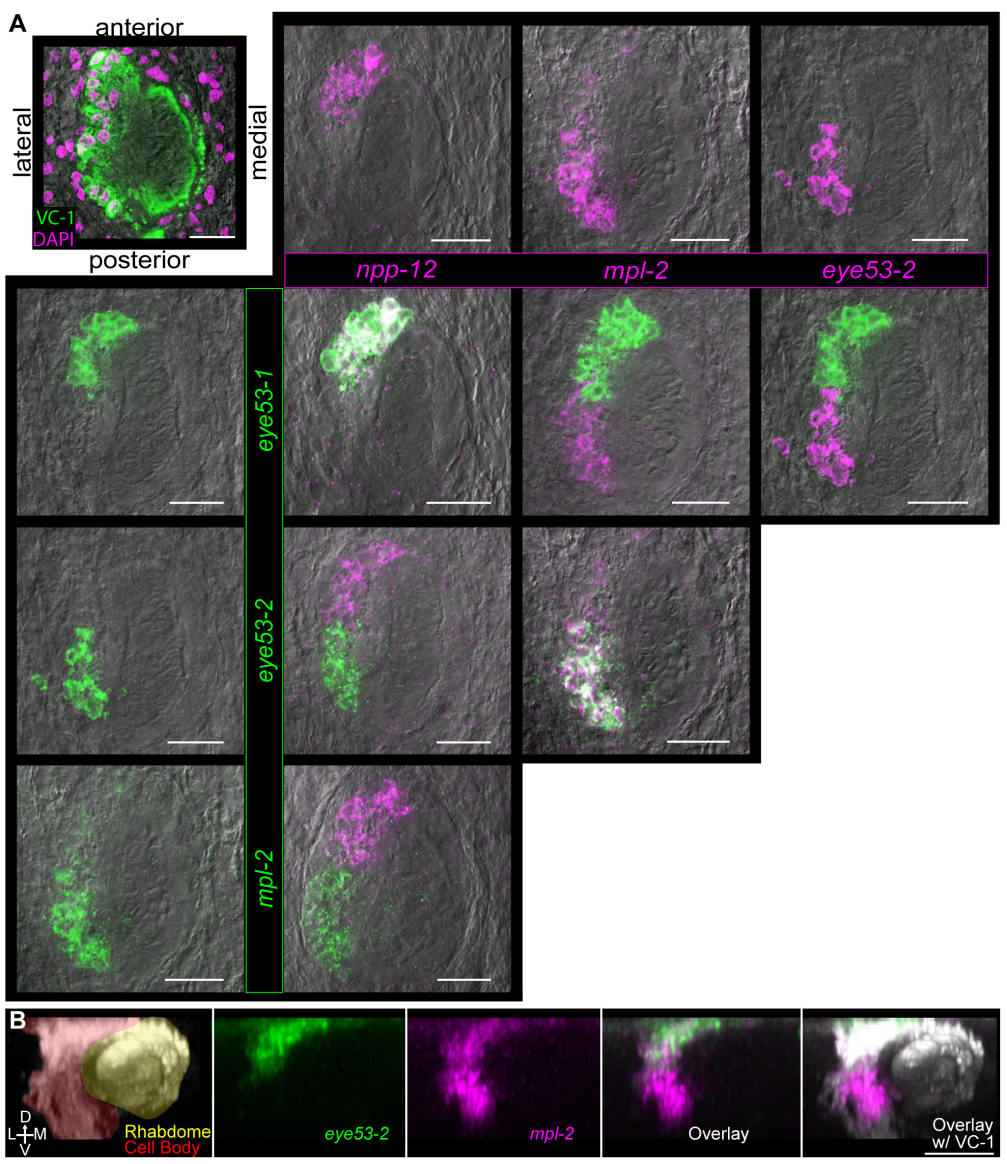
Prohormone gene expression reveals distinct photoreceptor neuron domains. (A) Double FISH showing expression of prohormone genes in anterior and posterior photoreceptor neurons. Top left, DAPI staining (magenta) and immunofluorescence with VC-1 antibody that recognizes arrestin (green) [63] to show the photoreceptor cell bodies (magenta surrounded by green) and their projections (green); image also indicates orientation for the other images in the panel. Remaining images are a matrix showing FISH for each prohormone gene expressed in the photoreceptors in comparison to the other three genes. All panels are shown overlaid with differential interference contrast optics. Dorsal view, anterior towards top. (B) Prohormones *mpl-2* and *eye53-2* are expressed differentially along the dorsal-ventral (D-V) axis of the photoreceptors. Shown is a maximum projection of a confocal XZ-series through the photoreceptors. Left, staining with the VC-1 antibody showing the photoreceptor cell bodies (pseudocolored red) and their rhabdomeric projections (pseudocolored yellow). Lateral (L) and medial (M) domains are indicated. Middle three panels, FISH with *mpl-2* and *eye53-2*; colors are indicated at bottom. Right, FISH and immunofluorescence with the VC-1 antibody (grey). Posterior view, dorsal towards top, medial towards right. Scale bars, 25 µm.

We also examined four prohormone genes (*eye53-1,-2*; *npp-12*, *and mpl-2*) expressed within the photoreceptors. The planarian photoreceptors are comprised of two distinct cell types: neuronal photoreceptive cells and pigment cells that envelop the rhabdomeric projections of the photoreceptor neurons [2],[65],[66]. Analysis of prohormone gene expression within the photoreceptors revealed that the planarian photoreceptor neurons are patterned along the anterior-posterior axis. Specifically, prohormone genes *npp-12* and *eye53-1* were expressed exclusively in the anterior photoreceptor neurons, whereas *mpl-2* and *eye53-2* were expressed exclusively in posterior neurons (Figure 5A). These findings are consistent with dye-tracing studies demonstrating that anterior and posterior photoreceptor neurons project to distinct anatomical regions [67]. In addition, we detected *mpl-2* expression in a ventral population of cells that was separate from the expression of *eye53-2* (Figure 5B); this result suggests that the photoreceptors are also patterned along the dorsal-ventral axis. Together, these data indicate that at least three chemically and anatomically distinct sets of neurons are present in the planarian photoreceptors.

### Peptide Hormones Are Expressed Differentially in Sexual Planarians

**Figure 6:**
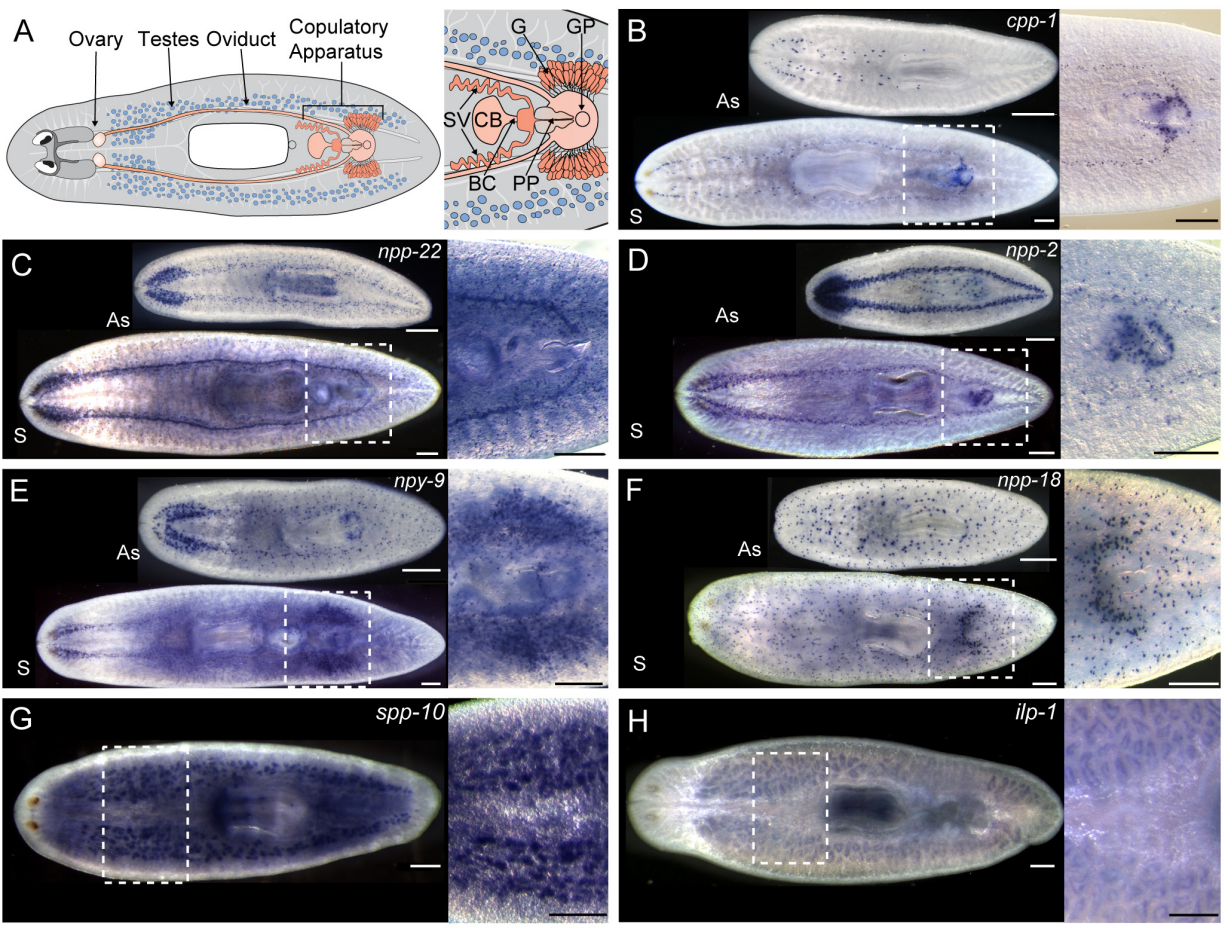
Several prohormone genes are expressed differentially in sexually reproducing planarians. (A) Diagram depicting the location of various organs in sexual *S. mediterranea*. Right, enlarged view of the copulatory apparatus. Abbreviations: SV, Seminal vesicles; CB, copulatory bursa; BC, bursa canal; PP, penis papilla; GP, gonopore; G, cement glands. (B–H) Genes are listed with their sexual-specific expression pattern; (B–F) expression in asexual (As) and sexual (S) animals is shown. (B) *cpp-1*; oviducts and penis papilla. (C) *npp-22*; oviducts and penis papilla. (D) *npp-2*; penis papilla. (E) *npy-9*; penis papilla and cement glands. (F) *npp-18*; gland cells surrounding copulatory apparatus. (G) *spp-10*; testes, (H) *ilp-1*; testes. Scale bars, 300 µm.

To determine if peptide expression is correlated with reproductive state, we next analyzed the expression of a subset of prohormones in the sexual strain of *S. mediterranea*. The reproductive system of this animal is comprised of a pair of ovaries located posterior to the cephalic ganglia, numerous dorsolateral testes lobes, as well as a variety of accessory reproductive organs (i.e. oviducts, sperm ducts, copulatory apparatus, and accessory glands) (Figure 6A). We found several prohormones expressed in sexual reproductive organs, including the oviducts (Figure 6B,C), the copulatory apparatus (Figure 6B,C, and D), gland cells surrounding the copulatory apparatus (Figure 6E,F), and the testes (Figure 6G,H). These expression patterns implicate peptide signaling in reproductive processes such as copulation, fertilization, egg-laying, and gonadal function.

**Figure 7:**
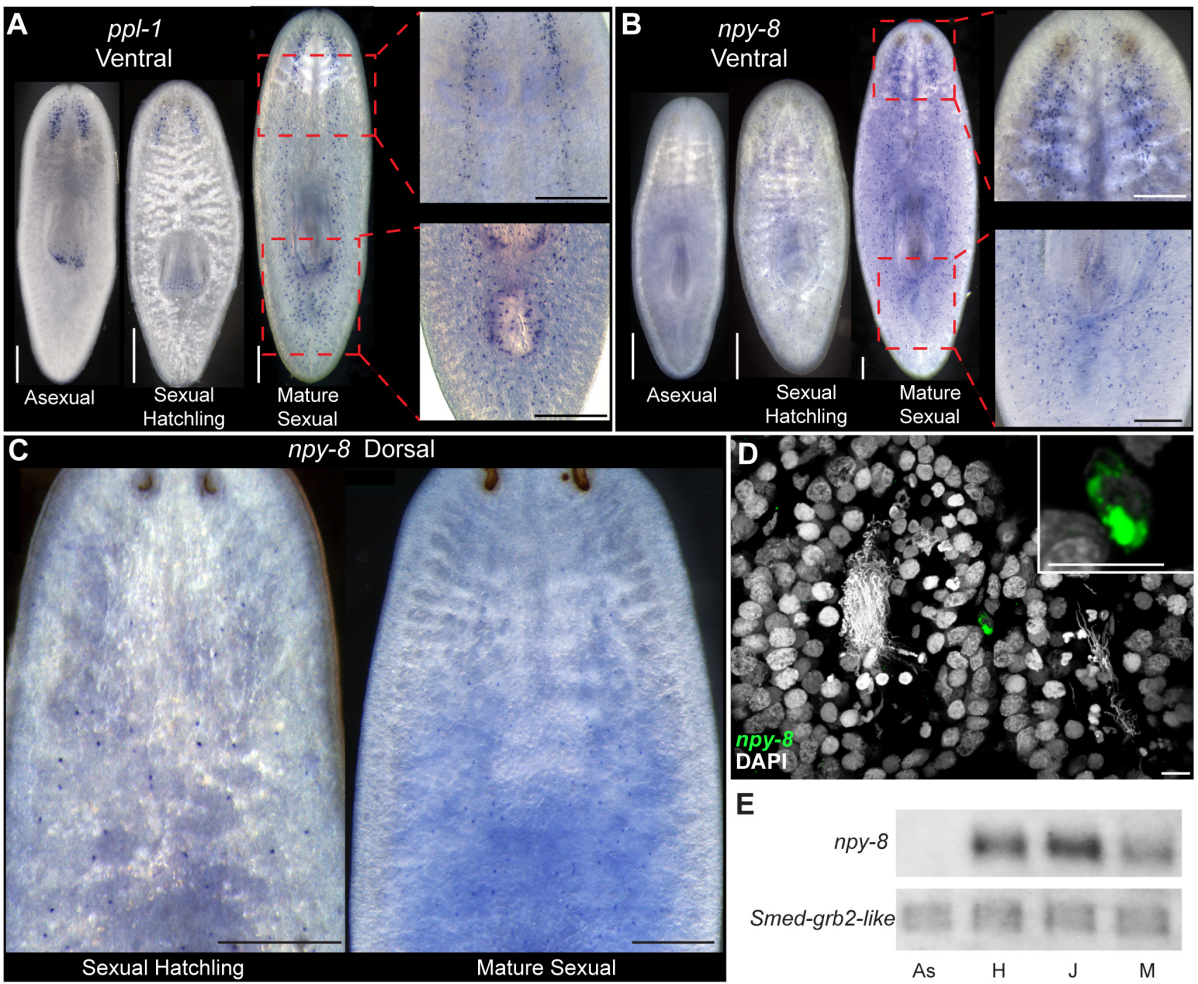
Some prohormone genes are expressed differentially in the CNS of sexual and asexual planarians. Comparison of the ventral expression of (A) *ppl-1* or (B) *npy-8* between asexual, immature sexual hatchlings, and mature sexual animals. (C) Dorsal expression of *npy-8* in immature sexual hatchlings (left) and mature sexual animals (right). (D) Transparency rendering showing expression of *npy-8* in a cell in close proximity to testes lobes. Inset shows higher magnification of *npy-8*-expressing cell. (E) Northern blot comparing expression of *npy-8* in asexual “As,” immature sexual hatchlings “H,” juvenile sexual animals “J,” and mature sexual animals “M.” *grb-2* (GB: DN305385) is expressed at similar levels in asexual and sexual animals (J. Stary and P. Newmark, unpublished observations) and is shown as a loading control. Scale bars: (A–C) 300 µm; (D) 10 µm.

Our expression analyses also found evidence of differential prohormone expression within the nervous system of sexual *S. mediterranea*. *ppl-1* encodes peptides related to the pyrokinin peptides originally isolated from arthropods [68],[69]. In contrast to asexual planarians in which *ppl-1* expression was detected almost exclusively in the cephalic ganglia and the distal region of the pharynx (Figure 3), *ppl-1* was expressed widely in the VNCs and surrounding the copulatory apparatus of mature sexual animals (Figure 7A). To explore if *ppl-1* expression was linked to sexual maturation, we determined the distribution of *ppl-1* in immature sexual animals. In sexual animals analyzed within one week of hatching from the egg capsule, *ppl-1* was expressed in a pattern similar to that of asexual animals (Figure 7A); thus, *ppl-1* expression undergoes a change in spatial distribution during the process of maturation.

**Figure 8:**
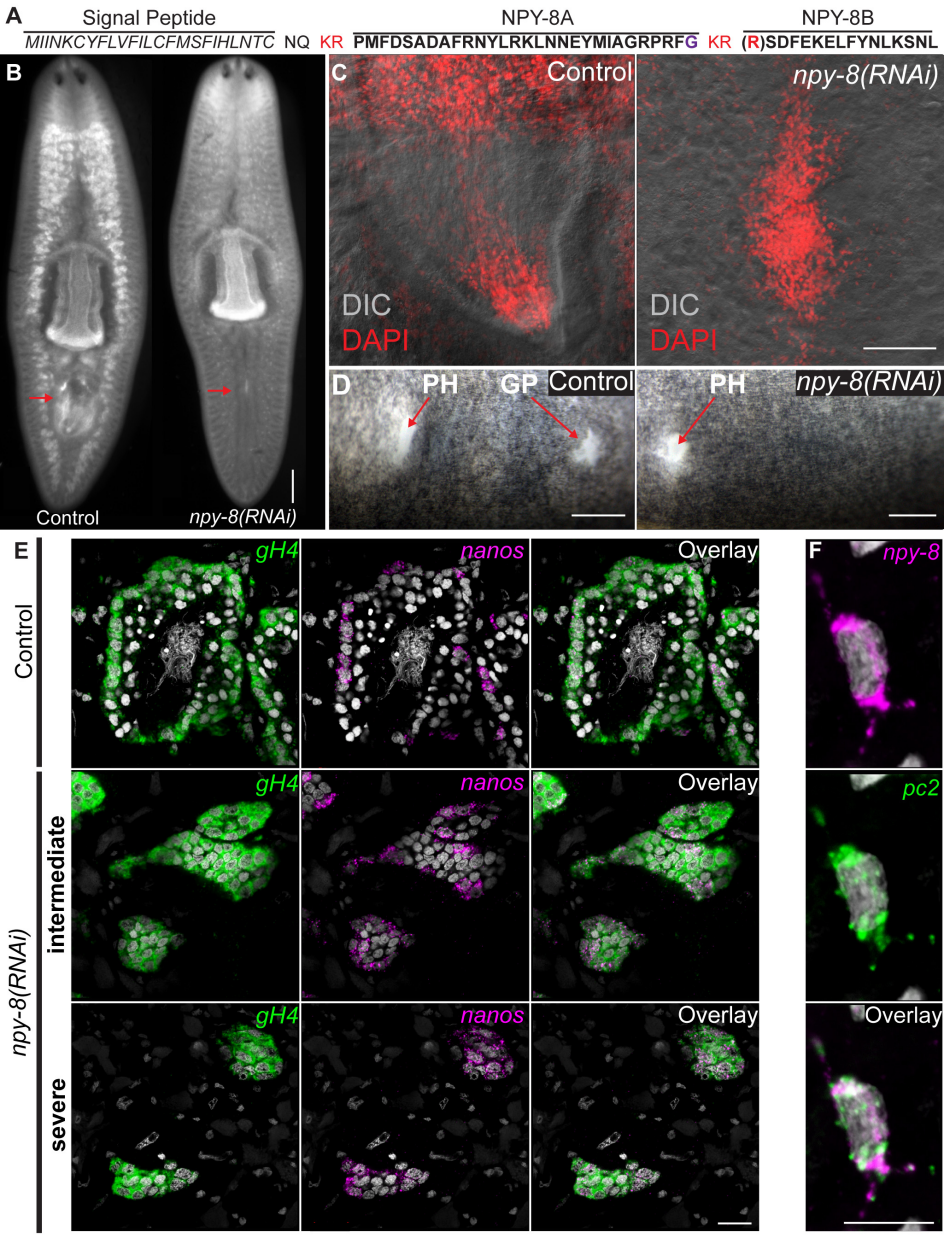
*npy-8* is required to maintain features of sexually mature planarians. (A) Sequence of the prohormone and predicted peptides encoded by *npy-8*. Following removal of the signal peptide (*italics*) the NPY-8 prohormone is predicted to be processed at two consensus prohormone convertase cleavage sites (red). This cleavage would result in two peptides: the C-terminally amidated peptide NPY-8A (potential amidation site is shown in purple) and the 15–16 AA peptide NPY-8B. (B) DAPI staining showing distribution of testes in control and *npy-8(RNAi)* animals at 4 wk after the first RNAi treatment. Arrows show region of the copulatory organs. (C) Penis papilla of control and *npy-8(RNAi)* animals visualized by DAPI staining (red) and differential interference contrast microscopy. Anterior towards top. (D) Ventral view of live control and *npy-8(RNAi)* animals showing the pharyngeal opening (PH) and the gonopore (GP). Anterior towards left. (E) Single confocal sections showing expression of *nanos* (magenta) and *gH4* (green) RNAs in testes of control (top) and *npy-8(RNAi*) animals that display either an intermediate or severe level of testes regression. DAPI staining is shown in gray. Animals were fixed ∼7 wk after the initiation of RNAi treatment. (F) Maximum confocal projection showing the localization of the *npy-8* and *pc2* transcripts surrounding the nucleus (gray) of a neuron at the level of the ventral sub-muscular neural plexus in a mature sexual animal. Similar co-localization was seen in other parts of the central and peripheral nervous systems (unpublished data). Scale bars: (B) 500 µm; (C–D) 300 µm; (E) 20 µm; (F) 10 µm.

The prohormone gene *npy-8* (GB: BK007010) is predicted to encode a 29 AA NPF-like peptide (NPY-8A) and a novel C-terminal peptide (NPY-8B) (Figure 8A). By in situ hybridization we failed to detect *npy-8* expression in asexual animals (Figures 3 and 7B). In mature sexual animals, however, *npy-8* RNA was detected in a variety of cells within the central and peripheral nervous systems including the cephalic ganglia, the VNCs, the sub-muscular plexus, and the pharyngeal nervous system (Figure 7B). Additionally, in a majority of animals (13/18) we detected *npy-8* RNA in a dorsal population of cells (Figure 6C). Analysis of this dorsal cell population by FISH localized *npy-8* expression to cells often, but not exclusively, found in association with testes lobes (Figure 7D). To determine if *npy-8* levels changed with sexual maturation we examined *npy-8* expression in sexual hatchlings. In recently hatched animals *npy-8* was detected in tissues similar to those of mature sexual animals including the cephalic ganglia, the VNCs, the sub-muscular plexus, and the pharyngeal nervous system (Figure 7B). Furthermore, we observed dorsal cells expressing *npy-8* in a majority of animals (8/13) (Figure 7C).

The lack of observable expression of *npy-8* in asexual animals by in situ hybridization suggested a relationship between *npy-8* expression and the ability to reproduce sexually. Because we initially cloned the *npy-8* gene by 3′ RACE with cDNA derived from asexual animals (Table S5), we wished to confirm our in situ hybridization results using an alternative approach. Therefore, we performed northern blot analyses to detect *npy-8* transcript in asexual, recently hatched sexual, juvenile sexual, and mature sexual animals (Figure 7E). Consistent with our in situ hybridization results, we detected high levels of *npy-8* in sexual animals of all developmental stages but not in asexual animals, suggesting that *npy-8* is expressed at negligible levels in asexual planarians.

### *npy-8* Is Required for the Maintenance of Reproductive Tissues

Because *npy-8* was expressed at high levels only in sexually reproducing planarians, we reasoned that peptides encoded from this gene may be important for reproduction. Therefore, we determined the knockdown phenotype of *npy-8* using RNAi. For this analysis we employed two distinct RNAi feeding regimens. First, we measured the effect of *npy-8* depletion on the maintenance of the reproductive system by feeding mature sexual animals bacterially expressed *npy-8* dsRNA and observing the structure of the reproductive system at 4- and 7-wk time points. As a complementary approach, we fed juvenile sexual planarians in vitro synthesized dsRNA and observed the development of the reproductive system after 1 mo of feeding. Mature sexual animals fed *npy-8* dsRNA over the course of 4–7 wk displayed a range of phenotypes consistent with loss of sexual maturity (data are summarized in Table 2). Specifically, in comparison to controls, a majority of *npy-8(RNAi)* animals had regressed testes and failed to produce mature sperm (1/18 for controls versus 14/21 for *npy-8* RNAi) (Figure 8B). In addition to testes defects, *npy-8(RNAi)* treatment resulted in regression of the copulatory organs (0/18 for controls versus 13/20 for *npy-8* RNAi) (Figure 8B,C) and a decrease in the size (or complete disappearance) of the gonopore (unpublished data). Similar to mature sexual animals, juvenile planarians fed *npy-8* dsRNA for 1 mo displayed stunted testes growth, failed to produce mature sperm (0/8 for controls and 6/8 for *npy-8(RNAi)*), and had shrunken or absent gonopores (0/20 for controls and 16/20 for *npy-8(RNAi)*, Figure 8D). Importantly, these effects on reproductive maturation were not due to an overall defect in growth since *npy-8(RNAi)* and control animals grew to similar sizes over this time period (Figure S5A).

**Table 2:** Summary of *npy-8(RNAi)* experiments with mature sexual animals.

Treatment	Days Since First RNAi Treatment	Animals With Testes Producing Sperm	Animals With a Complete Set of Copulatory Organs^a^	Distribution of *nanos* ^+^ and *gH4* ^+^ Cells^b^
control	28	7/7	7/7	Not determined
*npy-8(RNAi*)	28	4/12	4/11	Not determined
control	52	7/7	7/7	7/7 normal
*npy-8(RNAi)*	52	2/5	1/5	1/5 normal
				2/5 intermediate
				2/5 severe
control	54	3/4	4/4	4/4 normal
*npy-8(RNAi)*	54	1/4	2/4	1/4 normal
				1/4 intermediate
				2/4 severe
**Control (Cumulative)**	**—**	**17/18**	**18/18**	**10/11 normal**
***npy-8(RNAi)*** ** (Cumulative)**	**—**	**7/21**	**7/20**	**2/9 normal**
				**3/9 intermediate**
				**4/9 severe**

a Animals were considered to have a full set of copulatory organs if a copulatory bursa, bursa canal, and penis papilla could be detected by DAPI staining.

b “Normal” describes animals in which *nanos* expression was detected in a subset of *gH4*-expressing cells in the spermatogonial layer of mature testes lobes. “Intermediate” describes animals with regressed testes that label almost exclusively with *gH4*; a subset of these *gH4*-positive cells express *nanos*. “Severe” describes animals with regressed testes that label almost exclusively with both *gH4* and *nanos*.

Since *npy-8* is a member of an expanded family of *NPY-like* genes in *S. mediterranea* (Figure 2C), we examined both the effectiveness and the specificity of our *npy-8* knockdowns. We fed juvenile planarians dsRNA specific to *npy-8* and monitored the transcript levels of *npy-8* and its closest relative, *npy-1*, by quantitative RT-PCR. This analysis found that *npy-8* RNAi treatment resulted in a statistically significant decrease in *npy-8* transcript levels while having no effect on *npy-1* mRNA levels (Figure S5B). To further explore the specificity of the *npy-8(RNAi)* phenotype, we performed a long-term feeding experiment in which we fed juvenile animals dsRNA specific to *npy-8* or either of its two closest relatives, *npy-1* or *npy-2*. In contrast to *npy-8* RNAi, neither *npy-1* nor *npy-2* RNAi treatments produced observable defects in the maturation of the planarian reproductive organs (Figure S5C). Collectively, these studies suggest that the effects of *npy-8(RNAi)* on reproductive development are due to specific disruption of *npy-8* function and suggest that off-target effects are unlikely.

To examine the regressed testes of *npy-8(RNAi)* animals, we performed FISH to detect *nanos* and *gH4* expression. This analysis uncovered a range of phenotypes associated with *npy-8* RNAi (Figure 8E). Some *npy-8(RNAi)* animals had clusters of *gH4*-positive cells that were also *nanos*-positive; these testes clusters are similar to those observed in *pc2(RNAi)* animals (Figure 1G). In other animals we found *gH4*-positive clusters in which a subset of cells expressed *nanos*. We interpret the former to represent a “severe” *npy-8* knockdown phenotype, whereas we suggest that the latter represents an “intermediate” phenotype resulting from incomplete *npy-8* knockdown and/or perdurance of the peptide.

In the most severe cases, the testes regression phenotypes seen in *pc2(RNAi)* or *npy-8(RNAi)* animals were similar. One model to explain this observation is that PC2 is required for proteolytic processing of the NPY-8 prohormone, and loss of a mature peptide (or peptides) encoded by *npy-8* results in loss of the ability to achieve or maintain sexual maturity. Since our MS analysis did not identify any peptides encoded by *npy-8* in extracts from either asexual or sexual animals (Tables S1–S3), we used FISH to determine if *npy-8* and *pc2* transcripts are localized to similar cell types in the planarian nervous system. We found that *npy-8*-expressing cells within the cephalic ganglia, the VNCs, the pharynx, and the sub-muscular plexus also express high levels of *pc2* (Figure 8F; and unpublished data). This observation is consistent with PC2 being required for the processing of peptides encoded by the *npy-8* gene.

### Comparative Genomics Identifies Novel Peptide Hormones Encoded in the Genomes of Parasitic Flatworms

Related flatworms of the genus *Schistosoma* currently infect over 200 million people worldwide [70]. Because of their complicated life cycles, schistosomes are not readily amenable to the types of large-scale biochemical analyses that we have employed to characterize the planarian peptidome. As an indirect means of biochemically validating peptide sequences from these animals, we compared our MS-validated prohormones with predicted proteins from the genomes of the trematodes *Schistosoma mansoni* [40] and *Schistosoma japonicum* [71]. With this approach we validated the sequences of peptides from eight previously characterized schistosome prohormone genes (Tables 3 and S7) [39],[40]. Furthermore, we identified eight additional *Schistosoma* genes not previously annotated as peptide prohormones (Tables 3 and S7). Among these newly annotated prohormones are schistosome genes that encode the peptide YIRFamide, a well-conserved flatworm peptide that has potent stimulatory effects on schistosome muscle fibers [41] that was not identified in previous bioinformatic efforts [39],[40]. Together, these data provide biochemical validation for roughly half of the predicted prohormones in *Schistosoma* and demonstrate the utility of using planarians to understand flatworm parasites.

**Table 3:** Peptides detected in *S. mediterranea* that are conserved in *Schistosoma*.

*Schistosoma* Gene(s)^a^	S. *mediterranea* Gene	Predicted *Schistosoma* Peptide^c^	MS-Confirmed S. *mediterranea* Peptide^c^
*Sma-npp-23/Sja-npp-23*	*spp-11*	**YIRFG**	**YIRFG**
*Sma-npp-26*	*spp-15*	EH**FDPI** IY	**FDPI** M **F**a
		SY**FDPI** L **F**	**FDPI**Q**F**a
		SY**FDPI** IY	**FDPI**Q**F**G
		TL**F** N **PI** L **F**	
		N**FDPI** L **F**	
*Sja-npp-26*	*spp-15*	SY**FDPI** A **F**	**FDPI** M **F**a
		TY**FDPI** A **F**	**FDPI**Q**F**a
		N**FD**R**I** L **F**	**FDPI**Q**F**G
		N**FDPI** L **F**	
		SY**FDP**IA **F**	
		EY**FDPI** IY	
*Sma-npp-27/Sja-npp-27*	*1020HH-2*	VPP**Y** I **TGGIRY**	QS**Y** L **TGGIRY**
			**Y** L **TGGIRY**
*Sma-npp-28/Sja-npp-28*	*spp-18*, *-19*	A **YHFFRL**	G **YHFFRL**
*Sj-npp-1* b */Sm-npp-1* b	*npp-1*	A **FVRL**a	AS **FVRL**a
		G**FVRL**a	
		G**FVR** Ia	
*Sm-npp-5* b */Sj-npp-5* b	*npp-5*	A **A** YM**D** L **PW**a	PNWK**D** M **PW**a
		A **A** YI**D** L **PW**a	S **A** WR**D** M **PW**a
*Sm-npp-6* b */Sj-npp-6* b	*mpl-1*, *-2*	**AVRLMRLa**	**AVRLMRLa**
*Sj-npp-14* b */Sm-npp-14* b	*spp-4*	**GLR**N**MR** Ma	**GLR**L**MR** La

a Prefixes *Sma* and *Sja* are for genes from *S. mansoni* and *S. japonicum*, respectively.

b Prohormone genes described previously [39].

c Identical residues are shown in bold; similar residues are underlined. Lower case “a” indicates C-terminal amidation. All peptides except YIRFG were confirmed by tandem MS sequencing.

## Discussion

Traditional studies of neuropeptides have relied on the biochemical purification of individual peptides possessing interesting biological activities [72]. However, with the application of genomic and peptidomic technologies, a major bottleneck has been the characterization of this expanded collection of neuropeptide-encoding genes (and their encoded peptides) in vivo. Here we characterized peptide hormones in *S. mediterranea* using genomic, molecular, and biochemical approaches and determined the tissue-specific expression patterns for the entire collection of prohormone genes. Comparing the distribution of prohormone expression between asexual and sexual planarians, we identified a single prohormone gene, *npy-8*, as important for the maintenance of reproductive function. While our main focus was to understand the role of peptide hormones in planarian reproductive development, these studies lay the groundwork for using *S. mediterranea* as an experimental model for studies aimed at understanding the diverse functions of metazoan bioactive peptides.

### Diverse Prohormone Gene Expression Patterns Reveal Novel Biological Insights About Planarian Biology

Although previous studies have characterized the expression of subsets of prohormones or their corresponding peptides [73]–[76], a comprehensive accounting of the expression of these genes at the level of the whole animal has not yet been performed. Here we describe the distribution of all known neuropeptide-encoding genes in the planarian *S. mediterranea* by whole mount in situ hybridization. One surprising finding from these studies was the complexity of prohormone expression within the planarian CNS, which is considered to be among the most primitive centralized nervous systems in the animal kingdom [77]. We find that prohormone gene expression is localized to distinct regions of the cephalic ganglia and that many individual prohormones are expressed in unique CNS cell types. These results parallel observations in the planarian *D. japonica* in which small molecule neurotransmitters (e.g. serotonin and dopamine) are found in separate CNS cell populations [64]. The expression of prohormone genes in distinct regions/cell-types in the CNS suggests that processing centers for different neural functions (e.g. sensory, motor, and neuroendocrine) may be localized to chemically and spatially distinct domains of the flatworm CNS. In support of this idea, a “visual center” has been proposed to exist at the medial regions of the cephalic ganglia to which visual axons send their projections [78]. Elucidation of the functions of peptides expressed in these discrete CNS foci may help relate specific anatomical positions to distinct neural functionalities and allow for the dissection of planarian neural circuits.

Our analysis of prohormone expression also revealed that many prohormone genes are expressed in tissues of the reproductive tract. Expression of peptide prohormones has also been observed in the somatic reproductive organs of *C. elegans* [73]. Interestingly, the expression pattern of some planarian prohormones parallels the immunohistochemical localization of similar gene products in other invertebrates. The NPY family member *Smed-npy-9* was expressed in the cement glands (or shell glands) surrounding the copulatory apparatus that are thought to be involved in egg capsule synthesis and deposition [2],[79]. Studies of *S. mansoni* observed NPY-like immunoreactivity in the region of Mehlis' gland [80], which is morphologically, and likely functionally [79], similar to the glands labeled by *npy-9*. *cpp-1* encodes VPGWamide and TPGWamide, peptides that are related to the APGWamide peptides first described in molluscs [81]. We found *cpp-1* to be expressed around the penis papillia and the oviducts of sexual planarians, which mirrors APGWamide localization in the molluscan oviducts and male copulatory organs [82],[83]. While specific functions for any of these peptides in planarian reproductive function remain to be elucidated, these results suggest evolutionarily conserved roles for peptides in several reproductive organs.

Two prohormone genes (*ppl-1* and *npy-8*) were expressed differentially in the nervous systems of mature sexual versus asexual planarians. The expression of *ppl-1* was similar in asexual and immature sexual animals but underwent a dramatic change in distribution during sexual maturation. Conversely, *npy-8* expression was detected at similar levels and distribution in sexual animals yet was not detected in asexual animals. Interestingly, our biochemical analyses detected a number of peptides uniquely in either mature sexual or asexual planarians (Tables S1–S3). Taken together, these results indicate that sexually mature planarians possess unique signatures in both the composition and spatial distribution of peptide hormones relative to asexual and immature sexual animals.

### Peptide Hormone Signals Promote Planarian Sexual Maturation and Germ Cell Development

To address the role of peptide signaling in planarian reproductive physiology we first examined the planarian *prohormone convertase 2* orthologue, *pc2*. This analysis suggested that prohormone processing is required for regulating the dynamics of germ cell differentiation. A similar requirement for prohormone processing in germ cell development has not been described in other animal models. Loss-of-function mutations in the *C. elegans pc2* orthologue *egl-3* result in a range of neuromuscular defects [84],[85], but mutant animals are capable of germ cell development since they produce viable progeny. The role of the *Drosophila pc2* orthologue *Amontillado* has not been assessed in adult reproductive development due to a requirement for this gene at multiple points during embryonic and larval development [86],[87]. Despite the fact that peptide hormones are known to regulate vertebrate germ cells [11],[12], extensive studies of prohormone convertase knockout mice have also not revealed roles for prohormone processing in germ cell development [46]. Therefore, it is likely that functional redundancies exist among the enzymes responsible for processing hormones involved in vertebrate reproduction. Given this possibility of genetic redundancy in vertebrates, we suggest systematic characterization of prohormone processing in other invertebrate models (e.g. *C. elegans* and *Drosophila*) may help address the extent to which peptide signaling regulates reproductive development in other animals.

Our studies suggest that NPY-8 may be among the prohormones processed by PC2 that are required for normal sexual development. At present it is not known which of the two predicted peptides encoded by NPY-8 influence planarian reproductive physiology. Prohormones that encode NPY-like peptides, including NPY-8, often also encode a C-terminal peptide or CPON (C-flanking peptide of NPY) [39],[58],[88],[89]. Because the functions of both vertebrate and invertebrate CPON peptides remain elusive, we speculate that the NPY-related peptide NPY-8A is the functional unit of this prohormone. In vertebrates, NPY signaling is thought to elicit diverse effects on the neuroendocrine axis regulating reproduction. Depending on the hormonal milieu, NPY administration can either promote or inhibit surges of luteinizing hormone [90], a gonadotropin that regulates multiple functions in the male and female reproductive systems [10],[11],[13]. The hypothalamic gonadotropin-releasing hormone, which promotes luteinizing hormone release from the pituitary, can also be influenced by NPY [91],[92]. Additionally, NPY may influence the timing of sexual maturation in mammals since it has been suggested to either induce or inhibit the onset of puberty [93]. Since NPY is a well-known regulator of energy homeostasis, NPY has been suggested to coordinate reproductive function with nutrient status [94]. Studies of *Drosophila* and *Aplysia* indicate similar roles for NPY-like peptides in processes related to nutrient homeostasis, such as feeding behavior [56],[95]. However, functional analyses in vertebrate [96] and invertebrate models [56] have not described obvious reproductive deficits in animals deficient for NPY-like peptides. Given the fact that *S. mediterranea* possesses an expanded collection of NPY-like peptides relative to other animals, additional work will be required to determine whether the function of NPY-8 represents an ancestral or derived function for NPY-like peptides.

Coordinated signaling between the hypothalamus, the pituitary, and the gonads controls vertebrate reproduction. Although our initial observation with *pc2* RNAi implicated prohormone processing in planarian germ cell development, the site of action of this effect was difficult to interpret since *pc2* expression was detected in both the nervous system and the testes. Our studies of *npy-8* have clarified the role of the nervous system in planarian reproduction. *npy-8* is expressed in both the central and peripheral nervous systems, and its transcripts are not detected in tissues affected by *npy-8* RNAi, such as the testes. Therefore, peptides from NPY-8 are likely to act in a neuroendocrine fashion to influence reproductive development. Since amputation studies suggest that signals from the cephalic ganglia are essential for the maintenance of mature gonads in planarians [14],[15], one possible source of NPY-8 is from the cephalic ganglia.

The function of *pc2* within the testes is presently not known, but testes are likely to be a site of prohormone processing since we detect the expression of multiple peptide prohormones (*ilp-1* and *spp-10*) in this organ. Because peptide hormones can act as endocrine and paracrine signaling molecules in the vertebrate testes [12], it is possible that peptides play similar roles in planarians. Therefore, we propose that peptides (e.g. NPY-8 peptides) from the nervous system promote events associated with reproductive maturation (i.e. the production of differentiated germ cells) and peptides produced in the testes may provide feedback to the CNS and other organ systems about the physiological state of the gonads. Additionally, peptides expressed within the testes may serve as paracrine factors that regulate germ cell maturation. This possibility of coordinated signaling between CNS and the gonads may explain why the effects of *pc2* RNAi on the reproductive system are more severe than those of *npy-8* RNAi. Due to a lack of sufficient markers our studies have not examined the effects of neuropeptide signaling on ovarian development; future efforts will be directed at examining this question.

Although a chromosomal translocation distinguishes sexual and asexual *S. mediterranea* [42],[97], the strain-specific differences that account for their divergent modes of reproduction remain uncharacterized. With the exception of genes expressed in the reproductive system [98], little is known about the transcriptional differences between these strains. Here we identify *npy-8* as enriched in sexual animals and show an important role for this gene in sexual development. Interestingly, the regressed testes of mature sexual animals treated with either *pc2* RNAi or *npy-8* RNAi resemble the primordial germ cell clusters of asexual planarians that also label exclusively with *gH4* and *nanos* [44]. These observations, together with the loss of somatic reproductive structures in *npy-8(RNAi)* animals, suggest that lack of NPY-8 expression in asexual planarians may, in part, account for their inability to promote germ cell differentiation and initiate sexual maturation. However, because the phenotypes observed with *pc2(RNAi)* were more severe than those observed with *npy-8(RNAi)*, we anticipate future studies may uncover additional factors that act in concert with *npy-8* to influence planarian reproductive maturation.

### Studies of Planarians Will Help Us Inform the Biology of Parasitic Flatworms

According to one estimate, schistosomiasis (infection by *Schistosoma*) can be directly attributed to as many as 280,000 deaths per year in sub-Saharan Africa alone [99]. Despite the medical and economic impact of schistosomiasis, only a single chemotherapeutic agent (praziquantel) is currently used in treatment of this disease [100]. Therefore, identifying novel anthelmintic agents is an important goal of flatworm research. Schistosome eggs can become lodged in host tissues, such as the liver and bladder, forming granulomas that are the major cause of the pathology associated with schistosomiasis [100]. Thus, targeting reproductive function in adult animals represents a promising means by which to treat and control schistosome infection. The *S. mansoni* genome is predicted to encode two NPY-like prohormone genes: *Sm-npp-20a* and *Sm-npp-20b* [39],[101]. Comparison of the predicted peptides from these prohormones with NPY-like peptides from *S. mediterranea* found that the NPY-like peptide encoded from *Sm-npp-20a* shares its closest similarity to NPY-8A (∼48% identity, ClustalW) (Figure 2C). Given this observation, and the similarities in the reproductive anatomy between planarians and trematodes [2], it is possible that these animals employ similar mechanisms to control their reproductive output. Therefore, our results justify efforts aimed at understanding the role of peptide hormones in flatworm reproductive physiology and suggest that neuropeptide signaling may represent a viable target for the treatment and eradication of flatworm parasites.

## Materials and Methods

### Animal Culture

Sexual and asexual *S. mediterranea* were maintained at 20°C in 0.75× and 1.0× Montjuïc salts, respectively [102]. To minimize non-specific background from gut contents after feeding, animals were starved at least 1 wk prior to use. For all experiments with sexual *S. mediterranea*, sexually mature animals (∼1 cm in length, unless otherwise specified) with a well-developed gonopore were used, unless otherwise specified.

### Chemicals

All chemicals were obtained from Sigma-Aldrich (St. Louis, MO) unless otherwise stated. The peptide standards for Matrix-assisted laser desorption/ionization time-of-flight mass spectrometry (MALDI-TOF MS) calibration were purchased from Bruker Daltonics (Billerica, MA).

### Extraction of Peptides

For LC/MS analysis, peptide extracts were prepared from 80–100 sexual or asexual planarians. Whole animals were mechanically homogenized in 8–10 mL of acidified acetone (40∶6∶1 acetone/water/HCl) or acidified methanol (90∶9∶1 methanol/acetic acid/water). After sonication, vortexing, and centrifugation of the homogenate, the supernatant was collected and the organic solvent was removed by evaporation in a SpeedVac concentrator (Thermo Scientific, San Jose, CA). The supernatant was then filtered through a Microcon centrifugal filter with a 10 kDa cutoff (Millipore, Billerica, MA), evaluated for peptide content by MALDI-TOF MS sampling of 0.5 µL and subjected to sequential separations by HPLC prior to tandem MS for peptide identification.

### Peptide Separation and Measurement

Peptide extracts were fractionated using a microbore HPLC system Magic 2000 (Michrom BioResources, Inc., Aubum, CA) with a C18 reverse phase column (Dionex, 1,000 µm i.d., particle size 3 µm, and pore size 100 Å) at a 20 µL/min flow rate over a 70 min run. A four-step linear solvent gradient was generated by mixing mobile phases A (95% water and 5% acetonitrile (ACN), 0.1% formic acid (FA) and 0.01% trifluoroacetic acid (TFA), and B (95% ACN, 5% water, 0.1% FA, and 0.01% TFA) as follows: 5%–10% B in 20 min, 10%–50% B in next 30 min, 50%–80% B in next 10 min, isocratic 80% B for 5 min, 80%–5% B in 4 min. Fractions were manually collected, evaluated for peptide content by MALDI-TOF MS, and subjected to 2nd stage separation using a Micromass HPLC system (Manchester, U.K.) equipped with a C18 reverse phase column (Dionex, 300 µm i.d., particle size 3 µm, and pore size 100 Å) and coupled to a HCT Ultra ion-trap mass spectrometer via an electrospray ionization source (ESI) (Bruker Daltonics, Bremen, Germany). Second stage separation parameters were optimized individually for each fraction using either the same water/ACN solvent system or water/methanol with 0.1% FA as a counter-ion. Mass spectrometric detection of eluting peptides was controlled by the Esquire software (Bruker Daltonics, Bremen, Germany) in a data-dependent manner. Tandem MS ion precursor selection was limited to 3 ions per min sorted by signal intensity, preferred charge state was set to +2, and the active dynamic exclusion of previously fragmented precursor ions limited to 2 spectra per minute. The scan m/z ranges for MS and MS/MS were 300–1,800 and 50–3,000, respectively.

### Peptide Identification

For peptide identification, tandem mass spectra were converted to the .mgf file format (Mascot generic file) and processed for sequencing automatically using the PEAKS Studio 4.5 software (Bioinformatics Solutions, Inc., Waterloo, CA). PEAKS generated data were manually inspected and verified. Automatic sequencing was performed against an in-house planarian prohormone database using the following search parameters: cleavage sites, variable Post-Translational Modifications (PTMs) (including N-terminal pyro-Glu and pyro-Gln, C-terminal amidation, and disulfide bond; the maximum number of PTMs on a single peptide was set to four), mass tolerance equal 0.3 Da for the precursor ion, and 0.5 Da for fragments.

Criteria for peptide assignments and prohormone confirmation were based on confidence scores generated by PEAKS for each sequenced peptide and detection mass error. A PEAKS confidence score is given as a percentage value from 1% to 99% and represents the statistical likelihood that an amino acid sequence matches a given MS fragmentation spectrum. The PEAKS statistical algorithm considers factors such as signal to noise, total intensity, and spectrum tagging (PEAKS Studio Manual 4.5 http://www.bioinformaticssolutions.com/products/peaks/support/PEAKSStudioManual4.5.pdf). Our results are based on the current database of 51 prohormones. Our criteria for the validation of a prohormone include the identification of at least one peptide from the prohormone with a PEAKS score >80% and a mass accuracy ≤300 ppm, or with a score of >50% and a mass accuracy within 150 ppm. In addition, we manually verified automatic sequencing results, examined prohormone cleavage sites, and evaluated the possible PTMs of the identified peptides. A match of at least three consecutive fragments in an ion series from manual sequencing to an automatically generated peptide sequence was considered sufficient to validate the peptide assignment. As prohormone identification increases with the number of detected encoded peptides, we employed high identification criteria for the first peptide but allowed lower standards for assignment of additional peptides from the same prohormone (PEAKS score >20%, mass accuracy ≤500 ppm) provided the fragmentation spectrum satisfied manual verification.

In cases in which a prohormone had already been confirmed by tandem MS, occasionally we assigned peptides by mass match with MALDI-TOF-MS data. Such assignments were based on a mass-match within 200 ppm to protonated molecular ions of peptides predicted by NeuroPred (http://neuroproteomics.scs.uiuc.edu/neuropred.html) [52]. These assignments are tentative since they are not accompanied by sequencing data.

### Gene Prediction and Annotation

Two distinct bioinformatic approaches were used to identify prohormone genes in the *S. mediterranea* genome. First, similarity searches were performed with collections of peptides or prohormones from invertebrate species such as *Drosophila melanogaster*, *Aplysia californica*, *Apis mellifera* [32], *Caenorhabditis elegans* [73], and various Platyhelminthes [39] with stand-alone BLAST (BLOSSUM62 or PAM30 matrices and Expect values ≥10). Peptides and prohormones were obtained from genome databases (i.e. Wormbase, http://www.wormbase.org), from NCBI, or from an online catalog of bioactive peptides (http://www.peptides.be, [103]). Additionally, sequence tags generated by de novo MS sequencing of unassigned peptides were also used as queries for genomic BLAST searches (BLOSSUM62 or PAM30 matrices and Expect values ≥10). As an alternative to similarity searching we analyzed translated *S. mediterranea* EST [98],[104] and 454 (Roche, Mannheim, Germany) sequence data (Y. Wang and P.A. Newmark, unpublished) for sequences that possessed characteristics of prohormone genes including multiple dibasic cleavage sites and a signal sequence (www.cbs.dtu.dk/services/SignalP). Translations of nucleotide sequences were performed with longorf.pl, a script that translates the longest open reading frame in a nucleotide sequence (www.bioperl.org/wiki/Bioperl_scripts). Putative prohormone genes identified using these two approaches were used as queries to search the *S. mediterran*ea genome to determine if additional related prohormones existed in the genome. The full-length coding sequences of prohormone genes were predicted using a variety of gene and splice-site prediction tools, including NetGene2 (http://www.cbs.dtu.dk/services/NetGene2), FSPLICE (http://www.softberry.com), GENSCAN (http://genes.mit.edu/GENESCAN.html), and GeneQuest (v8.0.2, DNASTAR, Madison, WI). Where full-length sequences could not be predicted in silico, 5′ and 3′ Rapid Amplification of cDNA Ends (RACE) (FirstChoice RLM-Race Kit, Ambion, Austin, TX) analyses were performed following the manufacturer's protocol. The predictions of all genes reported here were independently verified by cDNA analysis (see below). Once verified, genes were considered to be genuine prohormone genes if they (1) possessed a signal sequence, (2) possessed basic cleavage sites that flanked predicted or MS-confirmed peptides, and (3) were less than 200 amino acids in length. Sequences were excluded if they shared similarity with genes previously annotated to be other than neuropeptide prohormones. All genes were named according to the *S. mediterranea* genome nomenclature guidelines [105].

### Comparison of Prohormones from *S. mediterranea* and *Schistosoma*

Translated nucleotide sequences were downloaded either from the *Schistosoma mansoni* FTP server (ftp.sanger.ac.ik/pub/pathogens/Schistosoma/mansoni) or from the NCBI taxonomy browser (http://www.ncbi.nlm.nih.gov/Taxonomy/). These sequences were then compared to the sequences of MS-confirmed *S. mediterranea* prohormones using BLASTP. NPY-family members were not included in this analysis, although three NPY-like proteins have been previously described in *Schistosoma* [39],[101]. Additionally we analyzed EST sequences in the NCBI database to identify schistosome prohormone genes. Newly annotated schistosome prohormones were analyzed further with SignalP and Neuropred to predict final gene products. These genes were named as described previously [39].

### Molecular Analyses

To facilitate efficient analyses of prohormone genes, we constructed a plasmid vector that permits TA-mediated cloning of PCR-amplified cDNAs. To generate a suitable vector backbone, oligonucleotide primers 5′-GATCACGCGTCGATTTCGGCCTATTGGTTA-3′ and 5′-GATCACGCGTGCTTCCTCGCTCACTGACTC-3′ were used to amplify the kanamycin and ampicillin resistance markers and the origin of replication of plasmid pCRII (Invitrogen, Carlsbad, CA); this PCR product was digested with *MluI* and ligated to generate a circular plasmid. Following circularization, an *Eam1105I* restriction site was removed from the *β-lactamase* gene of this plasmid by introduction of a silent mutation using site-directed mutagenesis (Quickchange II, Statagene, La Jolla, CA). For the functional elements of the vector, two mini genes were synthesized (Integrated DNA Technologies, Coralville, IA): T7TermSP6 and T7TermT3. T7TermSP6 included (5′ to 3′) *KpnI*, *MluI*, T7-terminator, *AscI*, T7 Promoter, SP6 promoter, GACCTTAGGCT (an *Eam1105I* site), and *XhoI*. T7TermT3 included (5′ to 3′) *SacI*, *MluI*, T7 terminator, T7 promoter, T3 promoter, GACCTTAGGCT (an *Eam1105I* site), and *NotI*. T7TermSP6 and T7TermT3 were shuttled to pBluescript SK II+ using the *KpnI* and *XhoI* sites from T7TermSP6 or the *SacI* and *NotI* sites from T7TermT3. These plasmids were digested with *MluI* and *EcoRI* and ligated with the *MluI* site of the vector backbone. A *XhoI* and *NotI*-digested PCR fragment including the *ccdB* and *camR* genes from plasmid pPR244 [47] were inserted to generate the final plasmid-pJC53.2. *Eam1105I* (Fermentas, Burlington, Ontario) restriction digest of this plasmid generates 3′ T overhangs that can be ligated to an A-tailed *Taq* polymerase-amplified PCR product [106]. The *ccdB* gene prevents any undigested plasmid from giving rise to viable clones [107]. Once cDNAs have been inserted into pJC53.2, riboprobes for in situ hybridization analysis can be generated by in vitro transcription with SP6 or T3 RNA polymerases and dsRNA for RNAi knockdowns can be generated by in vitro transcription with T7 RNA polymerase, or by transformation of *E. coli (HT115[DE3])* [108].

To generate riboprobes for in situ hybridization, prohormone genes not represented by EST clones [98] were PCR amplified (Platinum *Taq*, Invitrogen, Carlsbad, CA) from cDNA generated from total RNA (iScript cDNA Synthesis Kit, Bio-Rad, Hercules, CA) or 3′ RACE cDNA (RLM-RACE Kit, Ambion, Austin, TX) generated from either total or poly-(A)^+^ RNA (Poly-A Purist, Ambion, Austin, TX). For cDNA preparations, RNA was extracted using Trizol Reagent (Invitrogen, Carlsbad, CA). For cloning, 2–3 µL of PCR product was ligated with 70 ng of *Eam1105I*-digested pJC53.2 (Rapid DNA Ligation Kit, Roche, Mannheim, Germany) and used to transform DH5α. In vitro transcriptions with the appropriate RNA polymerase were performed using standard approaches with the addition of Digoxigenin-12-UTP (Roche, Mannheim, Germany), Fluorescein-12-UTP (Roche, Mannheim, Germany), or Dinitrophenol-11-UTP (Perkin Elmer, Waltham, MA).

In situ hybridizations were performed using the formaldehyde-based fixation procedure essentially as described previously [109]. However, due to their large size, sexual animals were killed in 10% N-Acetyl Cysteine, fixed for 20–30 min in 4% Formaldehyde in PBSTx (PBS+0.3% Triton X-100), permeabilized with 1% SDS (10 min at RT) prior to reduction (10 min at RT), and treated with 10 µg/mL Proteinase K (10–20 min at RT) after bleaching. Some samples were processed in either a BioLane HTI (Hölle & Hüttner, Tübingen, Germany) [98] or an Insitu Pro (Intavis, Koeln, Germany) hybridization robot [102]. Sexual animals were imaged with either a Microfire digital camera (Optronics, Goleta, CA) mounted on a Leica MZ12.5 stereomicroscope or a Leica DFC420 camera mounted on a Leica M205A stereomicroscope (Leica, Wetzlar, Germany). Both microscopes were equipped with a Leica TL RC base. Asexual animals were imaged over a piece of white filter paper and illuminated from above with an LED light source.

For FISH, following post-hybridization washes and blocking, animals were incubated in α-Digoxigenin-POD (1∶1000, Roche, Mannheim, Germany), α-Fluorescein-POD (1∶1000, Roche, Mannheim, Germany), or α-Dinitrophenol-HRP (1∶100, Perkin Elmer, Waltham, MA) overnight at 4°C, washed in MABT, equilibrated in TNT (100 mM Tris pH 7.5, 150 mM NaCl, and 0.05% Tween-20), and developed in Amplification Diluent containing a fluorescent-tyramide conjugate (Cy3-tyramide, Cy5-tyramide, or Fluorescein-tyramide; TSA-Plus, Perkin Elmer, Waltham, MA). Following development, animals were washed in TNT and HRP activity was quenched by a 1 h incubation in 1.5%–2.0% H_2_O_2_ dissolved in TNT. Following HRP inactivation, animals were washed in MABT, incubated in a different α-hapten-HRP antibody, and the process was repeated with a different fluorescent-tyramide conjugate. Samples were mounted in Vectashield (Vector Laboratories, Burlingame, CA) and imaged on a Zeiss LSM 710 confocal microscope (Carl Zeiss, Germany) (Plan-Apochromat 20×/0.8, C-Apochromat 40×/1.2 W korr UV-VIS-IR, or Plan-Apochromat 63×/1.4 Oil DIC objectives). Fluorescein, Cy3, and Cy5 were excited with 488 nm, 561 nm, and 633 nm lasers, respectively. Images were processed using either Zen 2008 (Carl Zeiss, Germany) or ImageJ [110].

Northern blot procedures were performed essentially as previously described [111] and hybridization signals were detected using an anti-digoxigenin alkaline phosphatase-conjugated antibody and chemiluminescence (CDP-STAR, Roche, Mannheim, Germany). Chemiluminescent signals were detected using a FluorChem Q (Alpha Innotech, San Leandro, CA).

Sequences of EST clones corresponding to *pc2* [43],[98] were assembled with one another and the *S. mediterranea* genome (Sequencher 4.7, Gene Codes, Ann Arbor, MI) to determine the full-length sequence and genomic structure of the *pc2* gene.

### RNAi Analysis

For RNAi analysis of *pc2*, EST clone PL05006A1C09 [98], which corresponds to *pc2*, was shuttled to plasmid pPR244 using a Gateway reaction (Invitrogen, Carlsbad, CA) [47]. For *npy-8* RNAi, a 3′ RACE product specific to *npy-8* was cloned in pJC53.2. RNAi feedings were performed essentially as described previously [112], with some modifications. In *pc2* RNAi experiments, ∼6.25 mL of IPTG-induced culture was pelleted, frozen at −80°C, and resupended in 30 µL of a mixture of homogenized beef liver and water. ∼5 mature sexual animals (>1 cm in length) received 1–2 feedings over the course of ∼48 h. *npy-8* RNAi experiments were performed similarly to *pc2* RNAi except feedings included 50% less bacteria and animals were fed every 5–7 d over the indicated time course; for some feedings, bacteria were omitted. On occasion, because of either refusal to feed or improper nutrition, some animals (both controls and treatment groups) decreased in size over the long time courses of the *npy-8* RNAi experiments. Therefore, only animals >1 cm in length at the time of fixation were included in our analyses at time points greater than 4 wk. For all RNAi experiments with bacterially expressed dsRNA, control feedings were performed with bacteria containing empty plasmid pPR242.

For RNAi experiments conducted with juvenile planarians, dsRNA was generated by in vitro transcription [113],[114]. To generate dsRNA, templates cloned in pJC53.2 were amplified with a modified T7 oligonucleotide (GGATCCTAATACGACTCACTATAGGG), cleaned up using the DNA Clean & Concentrator kit (Zymo Research, Orange, CA, D4003), and eluted in 10 µL of water. 4 µL of each PCR product was used as template for in vitro transcription in a reaction containing 5.5 µL DEPC-treated water, 5 µL 100 mM mix of rNTPs (Promega, E6000), 2 µL high-yield transcription buffer (0.4 M Tris pH 8.0, 0.1 M MgCl2, 20 mM spermidine, 0.1 M DTT), 1 µL thermostable inorganic pyrophosphatase (New England Biolabs, Madison, WI, M0296S), 0.5 µL Optizyme recombinant ribonuclease inhibitor (Fisher Scientific, Pittsburg, PA, BP3222-5), and 2 µL HIS-Tagged T7 RNA polymerase [115]. Samples were incubated at 37°C for 4–5 h and then treated with RNase-free DNase (Fisher Scientific, Pittsburg, PA, FP2231). Synthesized RNA was then melted by heating at 75°C, 50°C, and 37°C each for 3 min. 2.5–10 µg of each dsRNA solution was mixed with 45 µL of 3∶1 liver to water mix and used to feed up to 8 worms. For these experiments, animals without visible gonopores (juveniles) were fed every 4–5 d for the indicated time period and starved 1 wk before fixation. Unless otherwise specified, as a negative control, animals were fed dsRNA synthesized from the *ccdB* and *camR*-containing insert of pJC53.2.

To analyze the structure of the testes, animals were killed in 2% HCl for 3 min, fixed in either Methacarn (6 MeOH:3 Chloroform: 1 Glacial Acetic Acid) or 4% formaldehyde for 1–2 h, dehydrated in MeOH, bleached in 6% H_2_O_2_ in MeOH, and stained with 4′,6-diamidino-2-phenylindole (DAPI) (Sigma-Aldrich, St. Louis, MO). Alternatively, samples were processed for in situ hybridization, as described above. Following staining, animals were mounted in Vectashield, flattened, and imaged on either a Zeiss SteREO Lumar (Carl Zeiss, Germany) or a Zeiss LSM 710 confocal microscope (DAPI was excited with a 405 nm laser).

### Animal Size Measurements

To examine if *npy-8(RNAi)* affected overall growth, animals were immobilized on ice and imaged on a Leica M205A stereomicroscope. The area of each animal was determined using ImageJ.

### Quantitative PCR

To examine transcript levels in *npy-8* knockdowns, juvenile animals were fed either liver homogenate or 45 µL of liver homogenate mixed with 2.5 µg of in vitro synthesized *npy-8* dsRNA. 7 d later RNA was extracted from individual planarians using Trizol Reagent (Invitrogen, Carlsbad, CA). Following DNase treatment (DNA-free RNA Kit, Zymo Research, Orange, CA), reverse transcription was performed (iScript cDNA Synthesis Kit, Bio-Rad, Hercules, CA) and quantitative PCR was conducted using Power SYBR Green PCR Master Mix (Applied Biosystems, Warrington, UK) and a 7900HT real-time PCR system (Applied Biosystems). Standard curves were generated from serial dilutions of either plasmid DNA containing the gene of interest (*npy-8* and *npy-1*) or from genomic DNA (*β-tubulin* GB: DN305397). All samples were measured in triplicate to account for pipetting error. Absolute quantities of each transcript were determined from the standard curves and the levels of *npy-8* or *npy-1* were normalized to the level of *β-tubulin* in each sample. The mean value (i.e. *npy-8*/*β-tubulin* or *npy-1*/*β-tubulin*) for each treatment (i.e. control or *npy-8(RNAi)*) was then compared using a Student's *t* test. The primers used for these studies were *npy-8* Forward AATCAGAAAAGGCCGATGTTTG, Reverse CAAATAGTTCCGAAAGGCATCAG; *npy-1* Forward GTCGACCAAGATTCGGTAAACG, Reverse CATTCTTTTATGAAAATCCCCTGT; *β-tubulin* F TGGCTGCTTGTGATCCAAGA R AAATTGCCGCAACAGTCAAATA.

### Analysis of Prohormone Processing Following *pc2* RNAi

To investigate the effect of *pc2* RNAi on the proteolytic processing of prohormones, peptide profiles were measured by MALDI-TOF MS and compared by principal component analysis followed by a *t* test in tissue extracts prepared from 7 individual control and 7 individual RNAi-treated animals. Extracts were prepared by homogenizing each specimen in 100 µL of acidified acetone (see above). Following centrifugation at 14,000× g for 15 min, supernatant was collected, dried in SpeedVac concentrator (Thermo Scientific, San Jose, CA), and reconstituted in 30 µL of 0.01% TFA. For MALDI-TOF MS analysis, 0.7 µL of each extract was spotted on a stainless steel sample holder and co-crystallized with 0.7 µL of freshly prepared concentrated DHB matrix (DHB: 2,5-dihydroxybenzoic acid, 50 mg/mL 50% acetone). Three technical replicates were sampled for each biological sample, 42 spots total. Positive ion mass spectra were acquired manually in 600–6,000 *m/z* region using a Bruker Ultraflex II mass spectrometer in linear mode with external calibration. For each spot 700 laser shots in 7 acquisitions were accumulated into a sum spectrum representative of a replicate.

For comparison of peptide profiles in control and *pc2(RNAi)* animals, raw MALDI-TOF MS data were loaded into an evaluation version of ClinProTools software (Bruker Daltonics, Bremen, Germany) using the following processing parameters: convex hull baseline subtraction, baseline flatness 0.2, mass range 1,000–6,000 *m/z*, Savizky-Golay smoothing over 1 *m/z* width with 11 cycles, data reduction factor of 10, null spectra exclusion enabled, recalibration with maximum peak shift of 200 ppm. All spectra were normalized to the total ion count (TIC) prior to PCA calculations. Sum spectra from technical replicates were grouped into a representative sample spectrum in ClinProTools, thus representing a biological replicate for statistical calculations. From representative sample spectra a mean spectrum was generated by ClinProTools to reveal general peptide features for control and *pc2(RNAi)* groups. Standard deviation of signal intensities among biological replicates was derived for each peak in the group profile. Unlimited peak picking on the base of maximal peak intensity and minimal signal-to-noise ratio of 6 was done on the mean spectrum representative of each sample group in order to take advantage of noise reduction effect due to spectra addition. Peptide profiles of mean spectra representative of biological replicates were compared by principal component analysis followed by Anderson-Darling (AD) normality test and paired Student's *t* test for peaks showing normal distribution. Peaks not showing a normal distribution (p_AD_≤0.05) were evaluated by the Wilcoxon or Kruskal-Wallis tests, respectively [116]–[118]. To decrease the number of false positives while computing individual peak statistics on highly complex spectra, the Benjamini-Hochberg procedure incorporated into ClinProTool was automatically applied for *p* value adjustment during analysis [119].

## Supporting Information

Figure S1 **The *Smed-pc2* gene.** (A) Predicted structure of the 2435 bp *Smed-pc2* transcript: 5′ untranslated region (UTR) (Purple, nucleotides 1–295), Coding region (Red, nucleotides 296–2243), and 3′ UTR (Yellow, nucleotides 2244–2435). An additional putative transcriptional start site was also detected 12 nucleotides upstream of the initiator methionine (unpublished data). The *Smed-pc2* locus occupies ∼20 kB on supercontig 98 of the *S. mediterranea* genome. (B) The *Smed-pc2* gene encodes a predicted 649 amino acid (AA) protein that shares significant identity with Proprotein Convertase Substilisin/Kexin Type 2 proteins from *H. sapiens* (60% identities, 72% positives; NP_002585.2), *D. melanogaster* (60% identities, 72% positives; NP_477318.1), and *S. mansoni* (69% identities, 81% positives; CAY17138.1). SMED-PC2 domains and functional regions are color coded as follows: secretory signal sequence (Purple; AA 1–16), autocatalytic cleavage site (Gray; AA 98–101), Peptidase-S8 domain (Pink; AA 158–465; PFAM domain PF00082, E-value 4.2×10^−108^), and Proprotein Convertase P-Domain (Yellow; AA 525–613; PFAM domain PF01483, e-value 1.9×10^−31^). Asterisks shown above bolded residues indicate amino acids comprising the putative catalytic core of SMED-PC2.
(0.30 MB TIF)

Figure S2 **The *S. mediterranea* genome is predicted to encode multiple prohormone convertase proteins.** Shown is a ClustalW alignment of a region of the Peptidase-S8 domain from PC2 with three related proteins predicted from the *S. mediterranea* genome [43]. Although these are the only predicted proteins with similarity to this region of the Peptidase-S8 domain, additional sequences in the *S. mediterranea* genome show similarity to other regions of PC2.
(4.34 MB TIF)

Figure S3 **The Planarin family of prohormones.** (A) A ClustalW alignment of prohormones SMED-SPP-6, -7, -8, -9, and -17. Matching residues are highlighted in yellow, basic cleavage sites are highlighted in green, and the signal sequence is highlighted in magenta. (B) The genomic organization of prohormone genes *Smed-spp-6*, *7*. These genes are located in close proximity to one another and are transcribed in opposite orientations. Given their sequence similarity and genomic organization, it is likely that the Planarin family of genes was expanded by a series of recent gene duplication events.
(0.20 MB TIF)

Figure S4 **Schematic representation of the distribution of prohormone gene expression in the planarian cephalic ganglia.** Cartoon depicting the distribution of some prohormone genes expressed in distinct regions of the cephalic ganglia (gray) and photoreceptors. Although *npp-12*, *eye53-1*, *mpl-2*, and *eye53-2* are all expressed in the cephalic ganglia, their expression is only depicted in the photoreceptors. Abbreviations: LB, Lateral branches; PR, photoreceptors; PC, pigment cups; OC, optic chiasma; and VNC, ventral nerve cords.
(1.45 MB TIF)

Figure S5 ***npy-8(RNAi)*** **does not affect animal growth or the activity of other** ***npy*** **genes.** (A) Area measurements of animals after 1 mo of being fed either control or *npy-8* dsRNA. *p* value from Student's *t* test is given above and error bars represent 95% confidence intervals. *n* = 19 for controls and *npy-8(RNAi)*. (B) Levels of either *npy-8* (left) or *npy-1* (right) transcripts normalized to *β-tubulin* mRNAs. *n* = 3 animals for controls and *n* = 5 animals for *npy-8(RNAi)*. *p* value from Student's *t* test is given above and error bars represent 95% confidence intervals. (C) DAPI staining showing distribution of testes in *npy-8(RNAi)*, *npy-1(RNAi)*, *and npy-2(RNAi)* animals ∼2 mo after the first RNAi treatment. *n* = 4 animals for each treatment. Scale bars: 1 mm.
(9.06 MB TIF)

Table S1 **Summary of MS analysis from sexual and asexual** ***S. mediterranea*** **.**
(0.08 MB PDF)

Table S2 **Peptides characterized by MS from sexual** ***S. mediterranea*** **.**
(0.12 MB PDF)

Table S3 **Peptides characterized by MS from asexual** ***S. mediterranea*** **.**
(0.13 MB PDF)

Table S4 **Peptide families encoded from** ***S. mediterranea*** **prohormone genes.**
(0.07 MB PDF)

Table S5 **Sequence information for** ***S. mediterranea*** **prohormone genes.**
(0.25 MB PDF)

Table S6 **Changes in characterized and uncharacterized peptides following** ***pc2(RNAi)*** **treatment.**
(0.09 MB PDF)

Table S7 **Prohormone genes from** ***Schistosoma*** **that encode peptides related to peptides from** ***S. mediterranea*** **.**
(0.10 MB PDF)

## Abbreviations

CNS: central nervous system
FISH: fluorescence in situ hybridization
*gH4*: *germinal histone H4*
GSCs: germline stem cells
LC: liquid chromatography
MALDI-TOF: matrix-assisted laser desorption/ionization time-of-flight
MS: mass spectrometry
NPY: neuropeptide Y
OVP: ovary maturing parsin
PTMs: Post-Translational Modifications
RNAi: RNA interference
*Smed-pc2*: *Smed-prohormone convertase 2*
sNPF: short Neuropeptide F
VNC: ventral nerve cord
