# Correction: Genome-Wide Analyses Reveal a Role for Peptide Hormones in Planarian Germline Development

**DOI:** 10.1371/journal.pbio.1002234

**Published:** 2015-08-14

**Authors:** James J. Collins, Xiaowen Hou, Elena V. Romanova, Bramwell G. Lambrus, Claire M. Miller, Amir Saberi, Jonathan V. Sweedler, Phillip A. Newmark

The authors would like to clarify the figure presentation of Figure 7. A corrected legend for Figure 7 is provided here.

**Figure 7:**
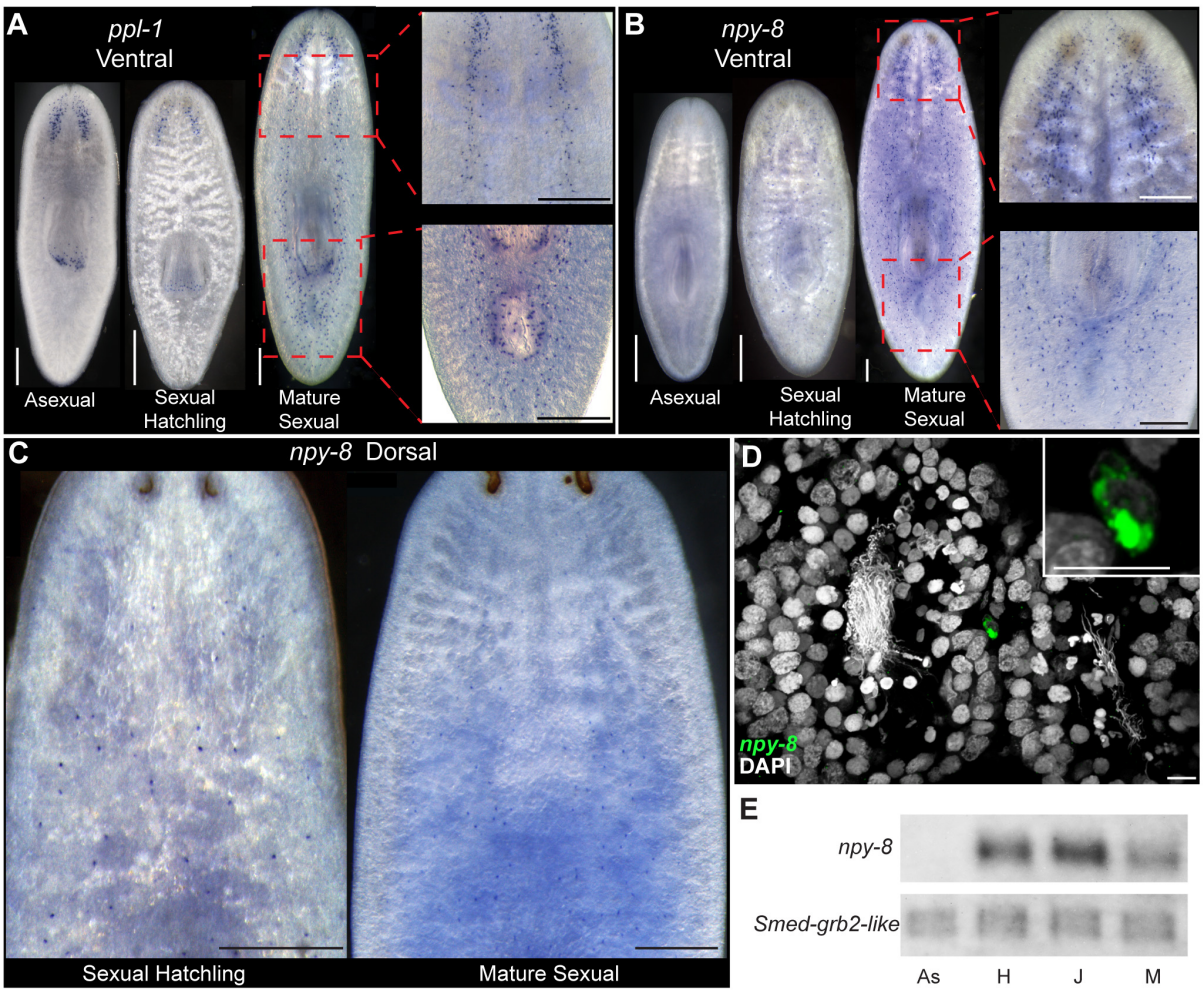
Some prohormone genes are expressed differentially in the CNS of sexual and asexual planarians. Comparison of the ventral expression of (A) *ppl-1* or (B) *npy-8* between asexual, immature sexual hatchlings, and mature sexual animals. Whole animal images were obtained using dark-field microscopy. Enlarged views were captured at higher magnification using Rottermann Contrast imaging. Magnified views do not necessarily represent the same focal plane or same individual shown in the whole-animal image. Red dashed boxes indicate the general body regions from which these images were obtained. (C) Dorsal expression of *npy-8* in immature sexual hatchlings (left) and mature sexual animals (right). (D) Transparency rendering showing expression of *npy-8* in a cell in close proximity to testes lobes. Inset shows higher magnification of *npy-8*-expressing cell. (E) Northern blot comparing expression of *npy-8* in asexual “As,” immature sexual hatchlings “H,” juvenile sexual animals “J,” and mature sexual animals “M.” *grb-2* (GB: DN305385) is expressed at similar levels in asexual and sexual animals (J. Stary and P. Newmark, unpublished observations) and is shown as a loading control. Scale bars: (A–C) 300 μm; (D) 10 μm.
