# Supplementary figures and images for: Genome-Wide Analyses Reveal a Role for Peptide Hormones in Planarian Germline Development
Source: PLoS Biol. 2010 Oct 12;8(10):e1000509. doi: 10.1371/journal.pbio.1000509 (PMC2953531; doi:10.1371/journal.pbio.1000509)

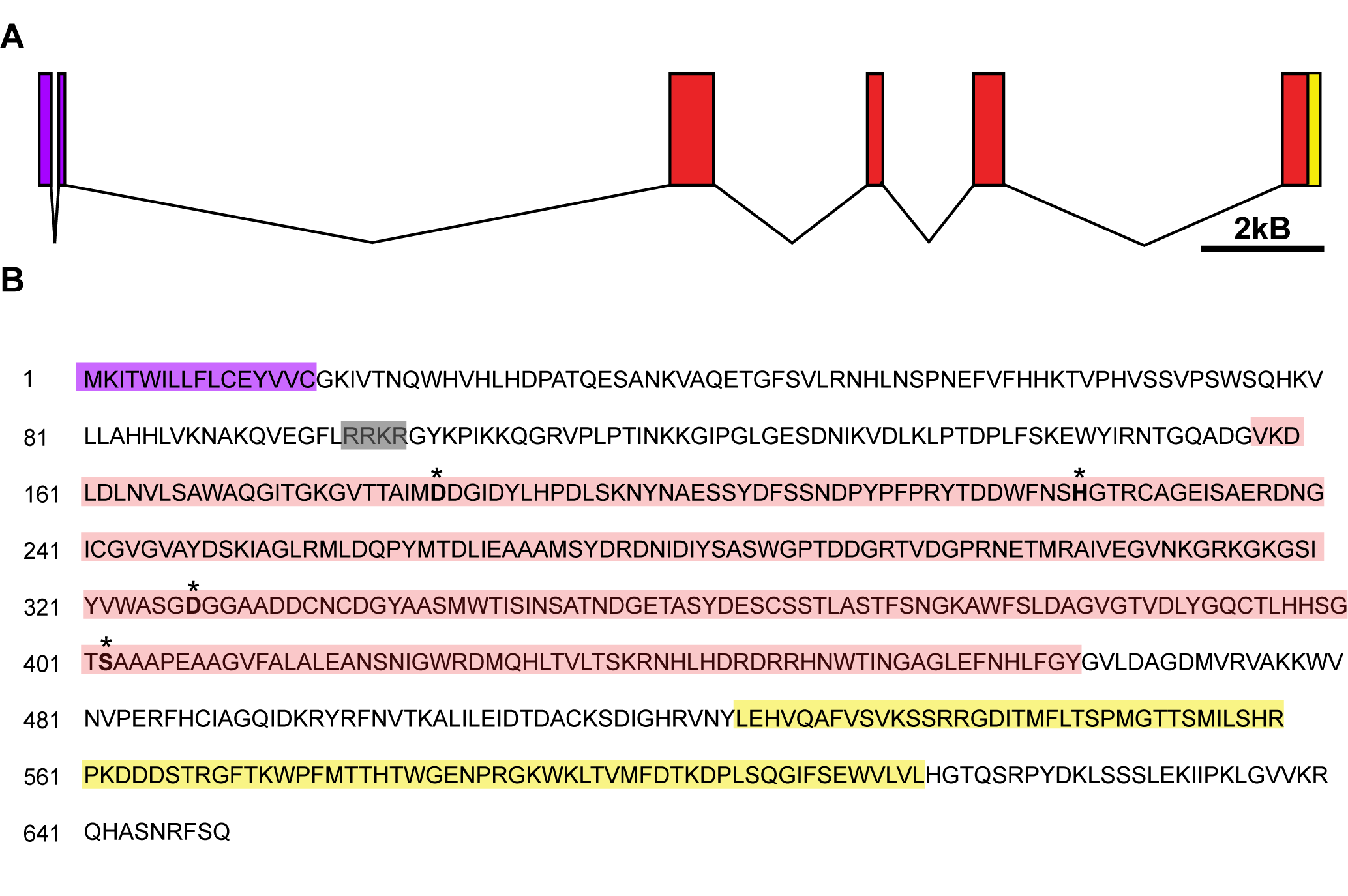

Supplement: Figure S1 — The Smed-pc2 gene. (A) Predicted structure of the 2435 bp Smed-pc2 transcript: 5′ untranslated region (UTR) (Purple, nucleotides 1–295), Coding region (Red, nucleotides 296–2243), and 3′ UTR (Yellow, nucleotides 2244–2435). An additional putative transcriptional start site was also detected 12 nucleotides upstream of the initiator methionine (unpublished data). The Smed-pc2 locus occupies ∼20 kB on supercontig 98 of the S. mediterranea genome. (B) The Smed-pc2 gene encodes a predicted 649 amino acid (AA) protein that shares significant identity with Proprotein Convertase Substilisin/Kexin Type 2 proteins from H. sapiens (60% identities, 72% positives; NP_002585.2), D. melanogaster (60% identities, 72% positives; NP_477318.1), and S. mansoni (69% identities, 81% positives; CAY17138.1). SMED-PC2 domains and functional regions are color coded as follows: secretory signal sequence (Purple; AA 1–16), autocatalytic cleavage site (Gray; AA 98–101), Peptidase-S8 domain (Pink; AA 158–465; PFAM domain PF00082, E-value 4.2×10−108), and Proprotein Convertase P-Domain (Yellow; AA 525–613; PFAM domain PF01483, e-value 1.9×10^−31^). Asterisks shown above bolded residues indicate amino acids comprising the putative catalytic core of SMED-PC2. (0.30 MB TIF) [file pbio.1000509.s001.tif]

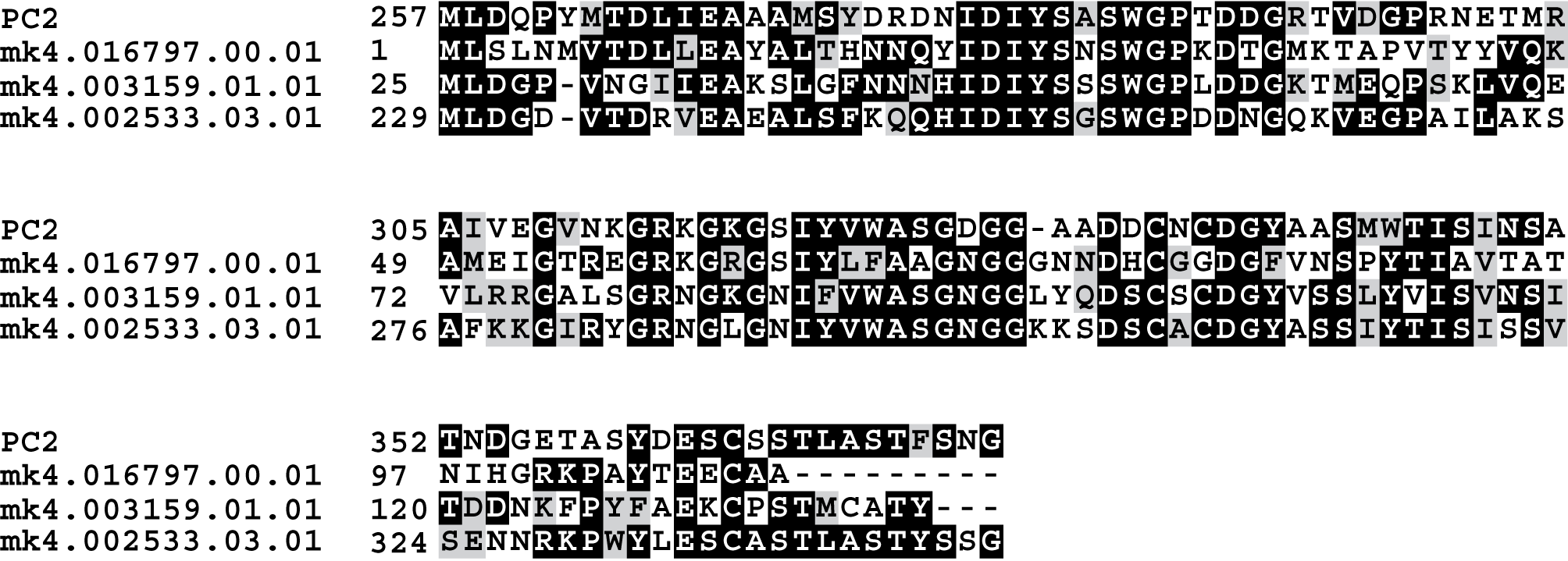

Supplement: Figure S2 — The S. mediterranea genome is predicted to encode multiple prohormone convertase proteins. Shown is a ClustalW alignment of a region of the Peptidase-S8 domain from PC2 with three related proteins predicted from the S. mediterranea genome [43]. Although these are the only predicted proteins with similarity to this region of the Peptidase-S8 domain, additional sequences in the S. mediterranea genome show similarity to other regions of PC2. (4.34 MB TIF) [file pbio.1000509.s002.tif]

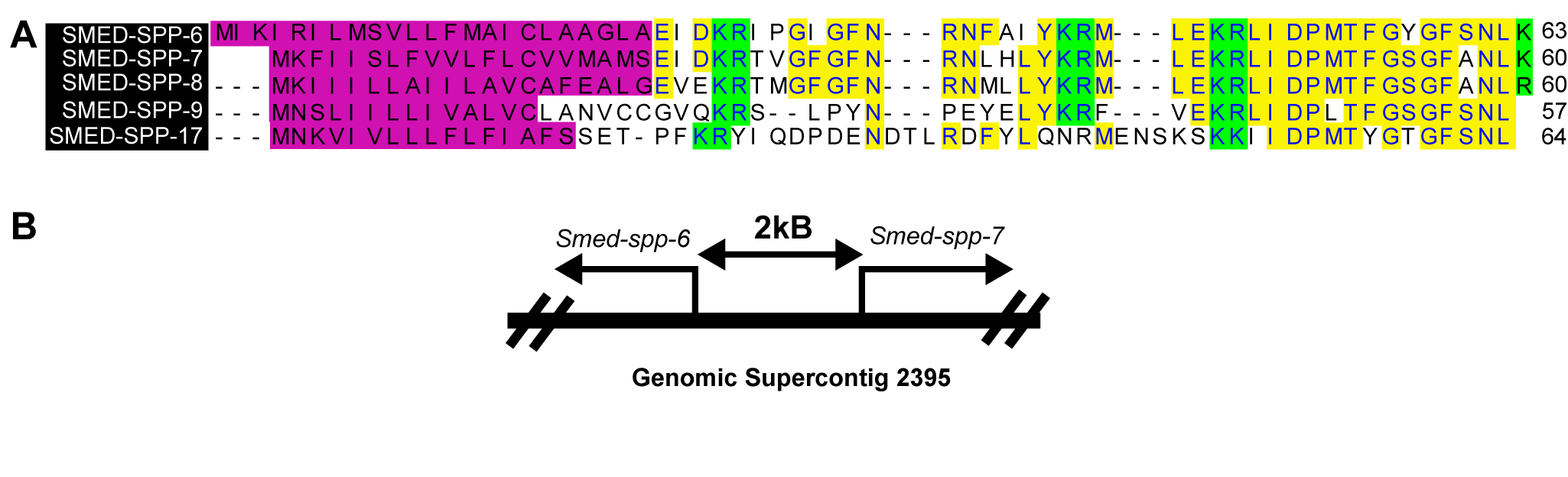

Supplement: Figure S3 — The Planarin family of prohormones. (A) A ClustalW alignment of prohormones SMED-SPP-6, -7, -8, -9, and -17. Matching residues are highlighted in yellow, basic cleavage sites are highlighted in green, and the signal sequence is highlighted in magenta. (B) The genomic organization of prohormone genes Smed-spp-6, 7. These genes are located in close proximity to one another and are transcribed in opposite orientations. Given their sequence similarity and genomic organization, it is likely that the Planarin family of genes was expanded by a series of recent gene duplication events. (0.20 MB TIF) [file pbio.1000509.s003.tif]

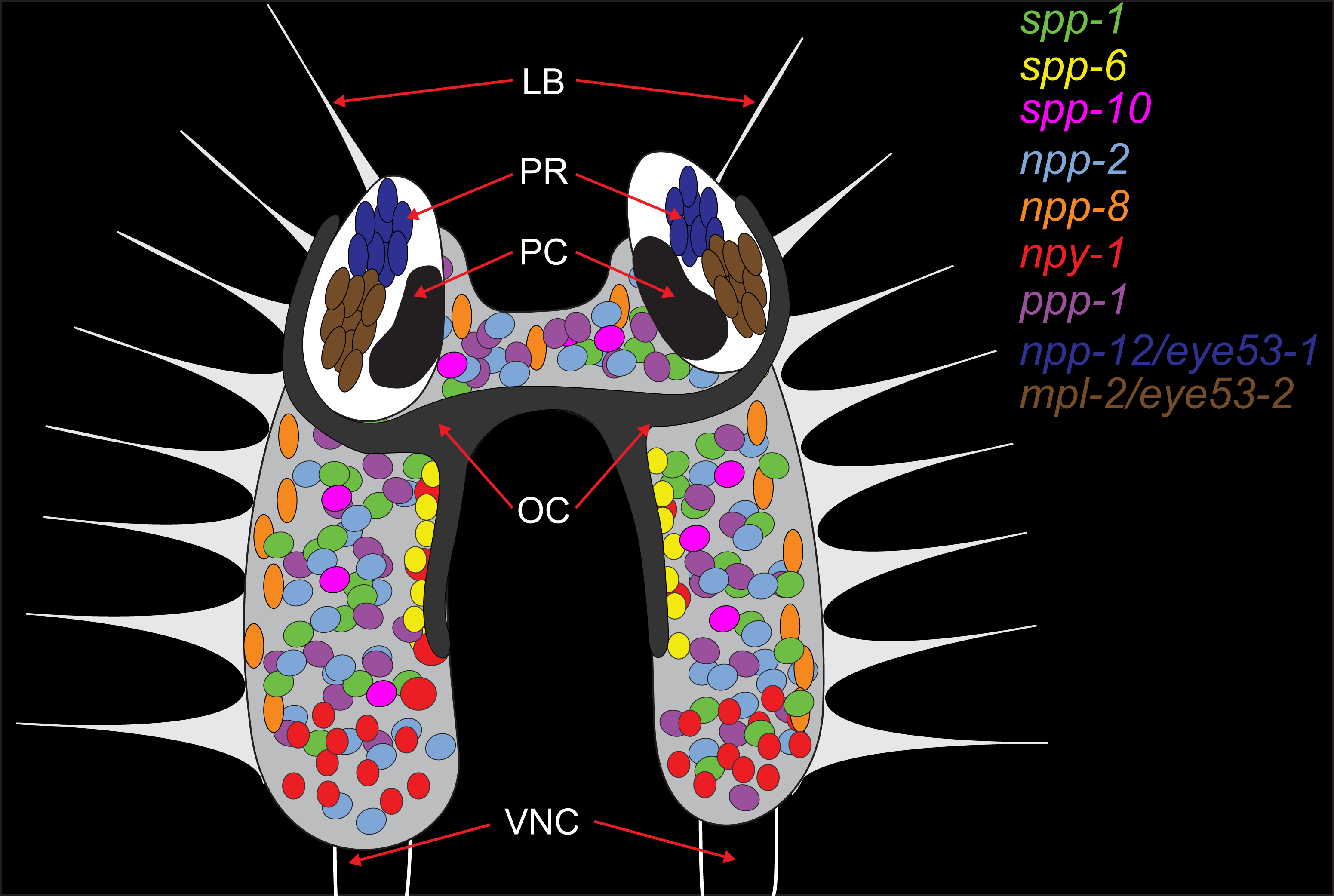

Supplement: Figure S4 — Schematic representation of the distribution of prohormone gene expression in the planarian cephalic ganglia. Cartoon depicting the distribution of some prohormone genes expressed in distinct regions of the cephalic ganglia (gray) and photoreceptors. Although npp-12, eye53-1, mpl-2, and eye53-2 are all expressed in the cephalic ganglia, their expression is only depicted in the photoreceptors. Abbreviations: LB, Lateral branches; PR, photoreceptors; PC, pigment cups; OC, optic chiasma; and VNC, ventral nerve cords. (1.45 MB TIF) [file pbio.1000509.s004.tif]

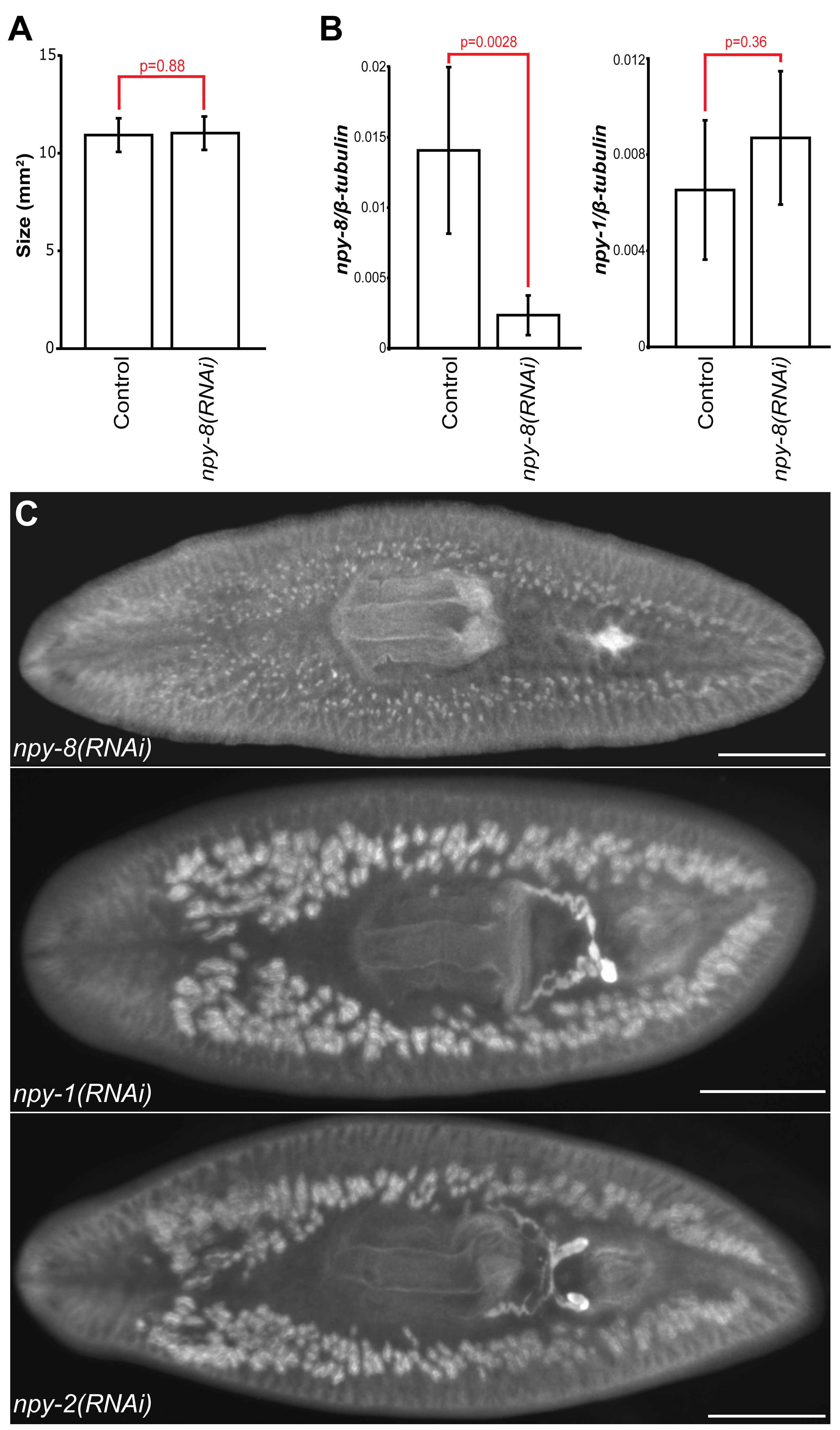

Supplement: Figure S5 — npy-8(RNAi) does not affect animal growth or the activity of other npy genes. (A) Area measurements of animals after 1 mo of being fed either control or npy-8 dsRNA. p value from Student's t test is given above and error bars represent 95% confidence intervals. n = 19 for controls and npy-8(RNAi). (B) Levels of either npy-8 (left) or npy-1 (right) transcripts normalized to β-tubulin mRNAs. n = 3 animals for controls and n = 5 animals for npy-8(RNAi). p value from Student's t test is given above and error bars represent 95% confidence intervals. (C) DAPI staining showing distribution of testes in npy-8(RNAi), npy-1(RNAi), and npy-2(RNAi) animals ∼2 mo after the first RNAi treatment. n = 4 animals for each treatment. Scale bars: 1 mm. (9.06 MB TIF) [file pbio.1000509.s005.tif]
