# Supplementary material for: Genome-Wide Analyses Reveal a Role for Peptide Hormones in Planarian Germline Development
Source: PLoS Biol. 2010 Oct 12;8(10):e1000509. doi: 10.1371/journal.pbio.1000509 (PMC2953531; doi:10.1371/journal.pbio.1000509)
Supplement: Table S1 — Summary of MS analysis from sexual and asexual S. mediterranea . (0.08 MB PDF) [file pbio.1000509.s006.pdf]

**Table S1. Summary of MS analysis from sexual and asexual *S. mediterranea***

| | Number of Peptides in Sexual animals | | | Number of Peptides in Asexual animals | | |
|---|---|---|---|---|---|---|
| Gene Name | Sequenced Peptides | Mass Match Peptides | Total | Sequenced Peptides | Mass Match Peptides | Total |
| *1020HH-1*$^{\Phi}$ | N/A | N/A | N/A | 2 | N/A | 2 |
| *1020HH-2* | 3 | N/A | 3 | 4 | N/A | 4 |
| *eye53-1* | 2 | N/A | 2 | 2 | N/A | 2 |
| *eye53-2*$^{\Phi}$ | 1 | N/A | 1 | N/A | N/A | N/A |
| *Ilp-1*$^{\Phi}$ | 2 | 1 | 3 | N/A | N/A | N/A |
| *mpl-1* | 1 | 1 | 2 | 3 | 2 | 4 |
| *mpl-2* | 1 | N/A | 1 | 3 | 1 | 3 |
| *npp-1* | 2 | 1 | 2 | 3 | 1 | 3 |
| *npp-2* | N/A | N/A | N/A | 1 | N/A | 1 |
| *npp-3* | 2 | 1 | 2 | 3 | 1 | 4 |
| *npp-4* | 2 | 4 | 4 | 3 | 2 | 5 |
| *npp-5* | 2 | 2 | 4 | N/A | N/A | N/A |
| *npp-18* | 6 | 1 | 6 | 6 | N/A | 6 |
| *npp-22* | 1 | 2 | 2 | 2 | 3 | 3 |
| *npy-1* | N/A | N/A | N/A | 3 | 1 | 4 |
| *npy-3* | N/A | N/A | N/A | 1 | 1 | 2 |
| *npy-5*$^{\Phi}$ | N/A | N/A | N/A | 2 | N/A | 2 |
| *npy-6* | N/A | N/A | N/A | 3 | N/A | 3 |
| *npy-7* | N/A | N/A | N/A | 2 | 1 | 3 |
| *npy-9* | 2 | N/A | 2 | N/A | N/A | N/A |
| *npy-10* | N/A | N/A | N/A | 2 | 1 | 3 |
| *ppp-1* | 5 | 4 | 6 | 7 | 2 | 7 |
| *ppp-2* | 4 | 2 | 4 | 3 | 2 | 3 |
| *spp-1* | 1 | 2 | 2 | 2 | 2 | 3 |
| *spp-3* | 5 | N/A | 5 | 5 | 1 | 5 |
| *spp-4* | 4 | 1 | 4 | 4 | 3 | 4 |
| *spp-5* | 4 | 1 | 4 | 3 | N/A | 3 |
| *spp-6* | 4 | 1 | 4 | 3 | 2 | 3 |
| *spp-7* | 2 | 2 | 2 | 2 | 1 | 2 |
| *spp-8* | 2 | 2 | 2 | 2 | 1 | 2 |
| *spp-9* | 3 | 1 | 3 | 4 | 2 | 4 |
| *spp-10* | 3 | 1 | 4 | 3 | N/A | 3 |
| *spp-11* | 2 | 2 | 3 | 2 | 2 | 3 |
| *spp-12* | 2 | 2 | 3 | 1 | 1 | 1 |
| *spp-13* | N/A | N/A | N/A | 1 | 1 | 1 |
| *spp-15* | 2 | 3 | 3 | 1 | 2 | 3 |
| *spp-16* | 5 | 2 | 5 | 6 | 3 | 7 |
| *spp-17* | 1 | N/A | 1 | 1 | N/A | 1 |
| *spp-18* | 1 | 1 | 1 | 1 | 1 | 1 |
| *spp-19* | 1 | 1 | 1 | 3 | 1 | 3 |

Four prohormones labeled with Φ are tentative and not completely validated, since they do not meet the criteria for prohormone identification as described in experimental section.
