# Supplementary material for: Genome-Wide Analyses Reveal a Role for Peptide Hormones in Planarian Germline Development
Source: PLoS Biol. 2010 Oct 12;8(10):e1000509. doi: 10.1371/journal.pbio.1000509 (PMC2953531; doi:10.1371/journal.pbio.1000509)
Supplement: Table S2 — Peptides characterized by MS from sexual S. mediterranea . (0.12 MB PDF) [file pbio.1000509.s007.pdf]

Table S2. Peptides Characterized by MS from Sexual *S. mediterranea*

Prohormone Name	Amino Acid Positions in Prohormone	Sequence	Experimental Mass (Da)	Cal. Mass (Da)	Error (ppm)	PEAKS Score	LC-ESI-IT-MS/MS	LC-MALDI-TOF-MS
1020HH-2	[52-61]	KR.pQSYLTGGIRY.KK	1139.57	1139.56	8.8	98	X	
1020HH-2	[52-61]	KR.QSYLTGGIRY.KK	1156.61	1156.59	17.3	99	X	
1020HH-2	[70-77]	KR.YLTGGIRY.#	941.51	941.5	10.6	99	X	
EYE53-1	[30-42]	KK.LSIPTYWDDIDTS.KR	1524.71	1524.7	6.6	98	X	
EYE53-1	*[49-60]*	*KR.LSVPTYFDDWES.RKKR*	*1457.54*	*1457.64*	*-68.6*	*99*	*X*	
**EYE53-2[Φ]**	**[60-71]**	**KKR.AVVPDAWDDWEI.KR**	**1415.71**	**1414.64**	**756.4**	**43**	**X**	
**ILP-1[Φ]**	**[21-49]**	***.EIFKYELYNQSQADLERNLEVRFCQHRLL.K**	**3654.23**	**3653.85**	**104**			**X**
**ILP-1[Φ]**	**[43-48]**	**R.FCQHRL.LK**	**802.47**	**802.39**	**99.7**	**25**	**X**	
**ILP-1[Φ]**	**[97-107]**	**RY.YSPESINCNAY.KK**	**1259.1**	**1259.51**	**-325.5**	**28**	**X**	
MPL-1	[43-49]	KR.AYRLMRMa.GKR	938.45	938.5	-53.3			X
MPL-1	[63-69]	KK.AVRLMRLa.GKR	856.58	856.54	46.7	53	X	
MPL-2	[45-51]	KK.AVRLMRLa.GKR	856.58	856.54	46.7	53	X	
NPP-1	[70-81]	KK.SRLDYEPVDDYN.KR	1484.62	1484.64	-13.5	98	X	X
NPP-1	*[84-93]*	*KR.ASFVRLGRTY.E#*[§]	*1168.75*	*1168.64*	*94.1*	*28*	*X*	
NPP-3	[56-62]	KR.AILLTRYa.GKR	847.58	847.53	59	98	X	
NPP-3	[80-86]	KR.AIVLTRFa.G#	817.8	817.52	342.5	95	X	X
NPP-4	[25-30]	KR.SSVFRFa.GKR	740.46	740.4	81	35	X	X
NPP-4	[25-31]	KR.SSVFRFG.KR	798.28	798.41	-162.8			X
NPP-4	[85-91]	KR.RGVAFRFa.GKR	850.61	850.49	141.1	74	X	X
NPP-4	[104-109]	KR.QSVFRYa.G#	797.31	797.43	-150.5			X
**NPP-5**	**[21-29]**	***.ENSDLIDAL.KKR**	**988.38**	**988.48**	**-101.2**			**X**
**NPP-5**	**[33-40]**	**KKR.PNWKDMPWa.GKR**	**1071.59**	**1071.5**	**84**	**52**	**X**	
**NPP-5**	**[44-51] (x5)**	**KR.SAWRDMPWa.GKR**	**1046.46**	**1046.48**	**-19.1**	**87**	**X**	
**NPP-5**	**[110-134]**	**KK.QGLHDLSLNNPFEKSLILNNSEFED.#**	**2873.64**	**2874.11**	**-163.5**			**X**
NPP-18	[28-42]	*.RNMDLDEYDSLLPKD.KR	1822.85	1822.84	5.5	99	X	X
NPP-18	[29-42]	*R.NMDLDEYDSLLPKD.KR	1666.65	1666.74	-54	97	X	
NPP-18	[34-42]	D.EYDSLLPKD.KR	1078.59	1078.52	64.9	51	X	
NPP-18	[45-54](x2)	KR.GAEFFIRRVVa.GKR	1191.68	1191.69	-8.4	98	X	
NPP-18	[45-55](x2)	KR.GAEFFIRRVVG.KR	1249.66	1249.69	24	35	X	
NPP-18	[71-77]	KR.NSDYLIQ.#	851.27	851.4	152.7	59	X	
NPP-22	[64-69](x3)	KR.AKYFRLa.GKR	795.45	795.48	-37.7	88	X	X
NPP-22	[64-70](x3)	KR.AKYFRLG.KR	853.32	853.49	-199.2			X
**NPY-9**	**[33-40]**	**K.YSLFSGPE.DLR**	**898.44**	**898.41**	**33.4**	**51**	**X**	
**NPY-9**	**[66-72]**	**KRN.pEWTSISE.#**[§]	**832.56**	**832.36**	**240.3**	**27**	**X**	
PPP-1	[35-46]	KK.YSYYDSIGSGLL.KR	1336.36	1336.62	-194.5	37	X	
PPP-1	[49-60]	KR.GAYYDPIGGGLL.KR	1194.66	1194.59	58.6	99	X	X
PPP-1	[63-74]	KR.SSYYDPIGGGLL.KR	1240.71	1240.6	88.7	88	X	
PPP-1	[77-88]	KR.DSNYDPIGGGLL.KRR	1219.66	1219.57	73.8	99	X	X
PPP-1	[91-102](x3)	KR.RSFYDPIGGGLL.KRR	1293.66	1293.67	-7.7	99	X	X
PPP-1	[133-154]	KK.RFYNDPLGVALLKSRFDKDSIN.#	2568.53	2568.92	-151.8			X
PPP-2	[34-45]	KR.FRYFDKIGSDLL.KR	1472.74	1472.77	-20.4	97	X	X
PPP-2	[48-58]	KR.SYFDKIGNDLL.KR	1283.61	1283.64	-23.4	98	X	X
PPP-2	[61-71]	KR.SYMDKIGSDLL.KRR	1240.62	1240.6	16.1	99	X	
PPP-2	[74-84]	KR.RYFDKIGSEML.KR	1357.72	1357.67	36.8	98	X	
SPP-1 A<br>B	[70-76](x3)<br>[36-42](x3)	KK.AYWASRMa.GKR	882.47	882.42	56.7	99	X	X

SPP-1	A<br>B	[70-77](x3)<br>[36-43](x3)	KK.AYWASRMG.KR	940.41	940.43	-21.3			X
SPP-3		[23-36]	*.SVMDDLSKDETYLS.KRR	1601.72	1601.71	6.2	80	X	
SPP-3		[39-47]	KR.RYSLINPRLa.GKR	1129.64	1129.67	-26.6	98	X	
SPP-3		[51-57]	KR.YLINPRLa.GKR	886.57	886.54	33.8	93	X	
SPP-3		[51-58]	KR.YLINPRLG.KR	944.54	944.54	0	95	X	
SPP-3		[61-70]	KR.FQIKDIENLD.#	1233.83	1233.62	170.2	99	X	
SPP-4		[45-51](x2)	KR.GLRLMRLa.GKR	856.54	856.54	0	46	X	
SPP-4		[45-52](x2)	KR.GLRLMRLG.KR§	914.39	914.55	-174.9	22	X	
SPP-4		*[56-65]*	*KRN.MNDEFQFRDL.KKR*	*1312.75*	*1312.59*	*121.9*	*42*	*X*	
SPP-4		[55-65]	KR.NMNDEFQFRDL.KKR	1427.6	1427.61	-7	99	X	X
SPP-5		[44-50]	KR.GLRILRMa.KR	856.54	856.54	0	59	X	
SPP-5		[44-51]	KR.GLRILRMG.KR	915.2	915.17	32.8	40	X	
SPP-5		[54-61]	KR.NDLFRLLD.KR	1004.5	1004.53	-29.9	93	X	X
SPP-5		*[56-61]*	*KRND.LFRLLD.KR* §	*775.48*	*775.46*	*25.8*	*25*	*X*	
SPP-6		[29-39]	KR.IPGIGFNRNFA.IYKR	1204.64	1204.64	0	68	X	
SPP-6		[29-41]	KR.IPGIGFNRNFAIY.KR	1480.8	1480.78	13.5	99	X	X
SPP-6		[49-62]	KR.LIDPMTFGYGFSNL.K#	1573.72	1573.75	-19.1	99	X	
SPP-6		*[52-62]*	*KRLID.PMTFGYGFSNL.K#*	*1232.57*	*1232.55*	*16.2*	*96*	*X*	
SPP-7		[26-38]	KR.TVGFGFNRNLHLY.KR	1536.74	1536.78	-26	96	X	X
SPP-7		[46-59]	KR.LIDPMTFGSGFANL.K#	1481.68	1481.72	-27	99	X	X
SPP-8		[26-38]	KR.TMGFGFNRNMLLY.KR	1562.69	1562.74	-32	99	X	X
SPP-8		[46-59]	KR.LIDPMTFGSGFANL.R#	1481.68	1481.72	-27	99	X	X
SPP-9		*[15-23]*	_*.LANVCCGVQ.KR_ §	*905.3*	*905.41*	*-121.5*	*44*	*X*	
SPP-9		[26-36]	KR.SLPYNPEYELY.KR	1386.66	1386.63	21.6	82	X	
SPP-9		[44-57]	KR.LIDPLTFGSGFSNL.#	1479.87	1479.76	74.3	99	X	X
SPP-10		[42-51]	KR.GAEFFLQRVEa.GKR	1193.52	1193.62	-83.8	94	X	
SPP-10		[55-64]	KR.GAEFFLRRVVa.GKR	1191.68	1191.69	-8.4	98	X	
SPP-10		[55-65]	KR.GAEFFLRRVVG.KR	1249.77	1249.69	64	37	X	
SPP-10		*[68-83]*	*KR.STKPIDPNQYPLVYGE.#*	*-1*	*1819.9*	*-65.9*			*X*
SPP-11		[24-28]	KR.YIRFG.KR	654.23	654.35	-183.4			X
SPP-11		[31-37]	KR.HQQLFPN.KR	882.32	882.43	-124.7	74	X	X
SPP-11		[47-52]	KR.EYIPLD.KR	748.22	748.36	-187.1	91	X	
SPP-12		[18-26]	*.EATPAMRSD.K §	976.55	976.43	122.9	36	X	
SPP-12		[40-54]	KR.NYMDFFGLNGDMQRF.KK	1853.72	1853.79	-37.8	99	X	X
SPP-12		[57-70]	KK.pQQFFRNHRPEIEWN.#	1882.52	1882.89	-196.5			X
SPP-15		[48-53](x8)	KR.FDPIQFa.GKR	-1	764.37	-157			X
SPP-15		[48-54](x8)	KR.FDPIQFG.KR	822.36	822.39	-36.5	82	X	X
SPP-15		[120-125]	KR.FDPIMFa.GR# §	767.33	767.37	-52.1	25	X	X
SPP-16		*[32-43]*	*KR.pQFDPIMYGKLRQ.FYRR*	*1477.45*	*1477.74*	*-196.2*	*64*	*X*	
SPP-16		[32-45]	KR.pQFDPIMYGKLRQFY.RR	1787.59	1787.87	-156.6	94	X	
SPP-16		[70-76](x2)	KR.QFDPIMY.KR	912.24	912.41	-186.3	69	X	X
SPP-16		[79-91]	KR.pQSNPYFLSDIRSI.KR	1521.69	1521.75	-39.4	99	X	X
SPP-16		[79-91]	KR.QSNPYFLSDIRSI.KR	1538.84	1538.77	45.5	99	X	
SPP-17		[51-64]	KK.IIDPMTYGTGFSNL.#	1527.72	1527.73	-6.5	99	X	
SPP-18		[29-35]	KR.GYHFFRL.KK	938.52	938.48	42.6	98	X	X
SPP-19		[42-48]	KR.GYHFFRL.RK	938.52	938.48	42.6	98	X	X

[a] Period (.) indicates a cleavage site. Asterisk (*) indicates the presence of signal peptide before the sequence. Pound (#) indicates the end of a precursor sequence. (x number) indicates the number of repeated sequences in this prohormone.

“X” indicates the analytical platform used to identify the peptide. Putative PTMs include N-terminal pyroglutamination (“p”) and C-terminal amidation (“a”). **Bold** type indicates prohormone precursors characterized only in sexual animals by mass spectrometry. Underlined peptides are only detected in sexual animals, while the peptides in *italic* are identified as different forms (such as modified by PTM, etc.) compared to the ones in asexual animals. In addition, peptides detected with lower confidence are marked as §. Two prohormones labeled with Φ are tentative and not confirmed, since they do not meet the criteria for prohormone identification as described in experimental section.
