# Supplementary material for: Genome-Wide Analyses Reveal a Role for Peptide Hormones in Planarian Germline Development
Source: PLoS Biol. 2010 Oct 12;8(10):e1000509. doi: 10.1371/journal.pbio.1000509 (PMC2953531; doi:10.1371/journal.pbio.1000509)
Supplement: Table S3 — Peptides characterized by MS from asexual S. mediterranea . (0.13 MB PDF) [file pbio.1000509.s008.pdf]

Table S3. Peptides Characterized by MS from Asexual *S. mediterranea*

Prohormone Name	Amino Acid Positions in Prohormone	Sequence	Experimental Mass (Da)	Cal. Mass (Da)	Error (ppm)	PEAKS Score	LC-ESI-IT-MS/MS	LC-MALDI-TOF-MS
**1020HH-1[Φ]**	**[29-41]**	**L.ENFDDLESNSDYM.KR**	**1576.44**	**1577.58**	**-722.6**	**51**	**X**	
**1020HH-1[Φ]**	**[44-53]**	**KR.YSYLKGGIRW.KK**	**1240.48**	**1241.66**	**-950.3**	**60**	**X**	
1020HH-2	[52-61]	KR.pQSYLTGGIRY.KK	1139.57	1139.56	8.8	97	X	
1020HH-2	[52-61]	KR.QSYLTGGIRY.KK	1156.51	1156.59	-69.2	96	X	
1020HH-2	*[53-61]*	*KRQ.SYLTGGIRY.KK*	*1028.52*	*1028.53*	*-9.7*	*99*	*X*	
1020HH-2	[70-77]	KR.YLTGGIRY.#	941.49	941.5	-10.6	99	X	
EYE53-1[Φ]	[30-42]	KK.LSIPTYWDDIDTS.KR	1525.45	1524.7	491.9	93	X	
EYE53-1[Φ]	*[49-61]*	*KR.LSVPTYFDDWESR.KKR*	*1613.09*	*1613.74*	*-402.8*	*23*	*X*	
MPL-1	[43-49]	KR.AYRLMRMa.GKR	938.52	938.5	21.3			X
MPL-1	[45-51]	KR.AVRLMRLa.GKR	856.53	856.54	-11.7	87	X	X
MPL-1	*[45-52]*	*KR.AVRLMRLG.KR*	*914.54*	*914.55*	*-10.9*	*51*	*X*	
MPL-1	[53-60]	KR.AVRLMRMG.KK	932.75	932.51	257.4	84	X	
MPL-2	[45-51]	KR.AVRLMRLa.GKR	856.53	856.54	-11.7	87	X	X
MPL-2	*[45-52]*	*KR.AVRLMRLG.KR*	*914.54*	*914.55*	*-10.9*	*51*	*X*	
MPL-2	[61-68]	KR.AVKLMRLG.KR [§]	886.28	886.54	-293.3	35	X	
NPP-1	*[41-46](x3)*	*KR.ASFVRLa.GR*	*690.39*	*690.418*	*-40.6*	*61*	*X*	
NPP-1	*[41-47](x3)*	*KR.ASFVRLG.R*	*748.38*	*748.42*	*-53.4*	*91*	*X*	
NPP-1	[70-81]	KK.SRLDYEPVDDYN.KR	1484.64	1484.64	0	99	X	X
**NPP-2**	**[110-123]**	**KR.pQLLNSKQSIFQDEY.#**	**1695.09**	**1694.82**	**159.3**	**94**	**X**	
NPP-3	[56-62]	KR.AILLTRYa.GKR	847.44	847.51	-82.6	94	X	
NPP-3	*[56-63]*	*KR.AILLTRYG.KR*	*905.6*	*905.53*	*77.3*	*99*	*X*	
NPP-3	[66-77]	KR.GSRRYFLNNDDS.KR	1442.67	1442.66	6.9			X
NPP-3	[70-77]	RR.YFLNNDDS.KR	986.72	986.4	324.4	58	X	
NPP-4	[25-30]	KR.SSVFRFG.KR	798.33	798.4	-87.7	44	X	
NPP-4	[85-91]	KR.RGVAFRFa.GKR	850.47	850.49	-23.5	99	X	
NPP-4	*[85-92]*	*KR.RGVAFRFG.KR*	*908.45*	*908.5*	*-55*	*99*	*X*	
NPP-4	[104-109]	KR.QSVFRYa.G#	797.44	797.43	12.5			X
NPP-4	*[104-110]*	*KR.QSVFRYG.#*	*855.47*	*855.43*	*46.8*			*X*
NPP-18	[28-42]	*.RNMDLDEYDSLLPKD.KR	1822.8	1822.84	-21.9	99	X	
NPP-18	[29-42]	*R.NMDLDEYDSLLPKD.KR	1666.65	1666.74	-54	99	X	
NPP-18	*[30-40]*	**RN.MDLDEYDSLLP.KDKR [§]*	*1309.22*	*1309.57*	*-267.3*	*41*	*X*	
NPP-18	[45-54](x2)	KR.GAEFFIRRVVa.GKR	1191.75	1191.69	50.3	87	X	
NPP-18	[45-55](x2)	KR.GAEFFIRRVVG.KR	1249.71	1249.69	16	93	X	
NPP-18	[71-77]	KR.NSDYLIQ.#	851.26	851.4	-164.4	71	X	
NPP-22	[26-61]	*.TTDDEVNENICQSLCHKSLSCLDECHDISESNDSME.KR	4044.74	4045.29	-136			X
NPP-22	[64-69](x3)	KR.AKYFRLa.GKR	795.46	795.48	-25.1	91	X	X
NPP-22	[64-70](x3)	KR.AKYFRLG.KR	853.59	853.48	128.9	83	X	X
**NPY-1**	**[25-37]**	**I.pEPPAKPEFFDDPE.LLR**	**1498.65**	**1498.67**	**-13.3**	**51**	**X**	
**NPY-1**	**[46-59]**	**KK.LNEYFAIVGRPRFG.KR**	**1638.11**	**1637.87**	**146.5**			**X**
**NPY-1**	**[48-57]**	**KKLN.EYFAIVGRPR.FGKR [§]**	**1206.94**	**1206.65**	**240.3**	**38**	**X**	
**NPY-1**	**[48-60]**	**KKLN.EYFAIVGRPRFGK.R**	**1538.75**	**1538.84**	**-58.5**	**47**	**X**	
**NPY-3**	**[26-34]**	***.NKDELDILF.KKR**	**1105.55**	**1105.57**	**-18.1**	**99**	**X**	
**NPY-3**	**[70-75]**	**KR.pQKFHRD.#**	**812.44**	**812.4**	**49.2**			**X**
**NPY-5[Φ]**	**[49-62]**	**RRY.LLQMNEYLAIVARP.RYGKR**	**1629.69**	**1629.89**	**-122.7**	**37**	**X**	
**NPY-5[Φ]**	**[56-65]**	**RRYLLQMNEY.LAIVARPRYG.KR**	**1114.54**	**1114.66**	**-107.7**	**45**	**X**	
**NPY-6**	**[22-39]**	***.ILSTFSSDPVIDFDLEKD.K [§]**	**2040.73**	**2039.99**	**362.7**	**45**	**X**	

**NPY-6**	**[22-40]**	***.ILSTFSSDPVIDFDLEKDK.D**	**2168.1**	**2168.09**	**4.6**	**65**	**X**	
**NPY-6**	**[65-78]**	**KR.SLNSFQTIREFVRD.RR**	**1710.9**	**1710.87**	**17.5**	**48**	**X**	
**NYP-7**	**[37-46]**	**K.SRTPIAGIVN.K** §	**1026.23**	**1026.58**	**-340.9**	**30**	**X**	
**NYP-7**	**[39-51]**	**R.TPIAGIVNKMGQI.RK**	**1340.57**	**1340.75**	**-134.3**			**X**
**NYP-7**	**[63-79]**	**L.VYLLNEHFAIYGRPRYG.#**	**2067.1**	**2067.07**	**14.5**	**55**	**X**	
**NPY-10**	**[38-64]**	**KR.DLKPLFNNAKQLLWYLQKLDKMYAIAG.R**	**3195.79**	**3195.81**	**-6.3**			**X**
**NPY-10**	**[59-64]**	**K.MYAIAG.R**	**624.29**	**624.29**	**0**	**52**	**X**	
**NPY-10**	**[61-68]**	**KMY.AIAGRPRYa.GKR** §	**902.23**	**902.05**	**199.5**	**33**	**X**	
PPP-1	[22-32]	*DF.YRVSSSDLRRF.KK	1384.66	1384.72	-43.4	66	X	
PPP-1	[35-46]	KK.YSYYDSIGSGLL.KR	1336.63	1336.62	7.5	93	X	
PPP-1	[49-60]	KR.GAYYDPIGGGLL.KR	1194.5	1194.59	-75.3	99	X	
PPP-1	*[50-60]*	*KRG.AYYDPIGGGLL.KR*	*1137.41*	*1137.57*	*-140.7*	*54*	*X*	
PPP-1	[63-74]	KR.SSYYDPIGGGLL.KR	1240.61	1240.6	8.1	93	X	X
PPP-1	[77-88]	KR.DSNYDPIGGGLL.KRR	1219.59	1219.57	16.4	68	X	
PPP-1	[91-102](x3)	KR.RSFYDPIGGGLL.KRR	1293.68	1293.67	7.7	94	X	X
PPP-2	[34-45]	KR.FRYFDKIGSDLL.KR	1472.73	1472.77	-27.2	93	X	X
PPP-2	[48-58]	KR.SYFDKIGNDLL.KR	1283.6	1283.64	-31.2	99	X	
PPP-2	[61-71]	KR.SYMDKIGSDLL.KRR	1240.61	1240.6	8.1	99	X	X
SPP-1	A [50-67]<br>B [16-33]	A.EDLGSLNADIDLDDSRLD.KK<br>*.EDLGSLNADIDLDDSRLD.KK	1974.36	1974.9	-273.4	85	X	
SPP-1	A [70-76](x3)<br>B [36-42](x3)	KK.AYWASRMa.GKR	882.31	882.42	-124.7	85	X	X
SPP-1	A [70-77](x3)<br>B [36-43](x3)	KK.AYWASRMG.KR	940.48	940.43	53.2			X
SPP-3	[39-47]	KR.RYSLINPRLa.GKR	1129.71	1129.67	35.4	77	X	X
SPP-3	*[39-48]*	*KR.RYSLINPRLG.KR*	*1187.67*	*1187.68*	*-8.4*	*81*	*X*	
SPP-3	[51-57]	KR.YLINPRLa.GKR	886.45	886.54	-101.5	99	X	
SPP-3	[51-58]	KR.YLINPRLG.KR	944.48	944.54	-63.5	99	X	
SPP-3	[61-70]	KR.FQIKDIENLD.#	1233.56	1233.62	-48.6	98	X	
SPP-4	[21-42]	*.DYSSLNDDSELEDSYHRYPSSI.KR	2591.1	2591.09	3.9	89	X	X
SPP-4	[45-51](x2)	KR.GLRLMRLa.GKR	856.53	856.54	-11.7	57	X	X
SPP-4	[45-52](x2)	KR.GLRLMRLG.KR	914.54	914.55	-10.9	46	X	
SPP-4	[55-65]	KR.NMNDEFQFRDL.KKR	1427.66	1427.61	35	99	X	X
SPP-5	[44-50]	KR.GLRILRMa.GKR	856.54	856.53	11.7	73	X	
SPP-5	[44-51]	KR.GLRILRMG.KR	914.55	914.55	0	70	X	
SPP-5	[54-61]	KR.NDLFRLLD.KR	1004.5	1004.53	-29.9	99	X	
SPP-6	[29-39]	KR.IPGIGFNRNFA.IYKR	1204.61	1204.64	-24.9	90	X	
SPP-6	[29-41]	KR.IPGIGFNRNFAIY.KR	1480.73	1480.78	-33.8	91	X	X
SPP-6	[49-62]	KR.LIDPMTFGYGFSNL.K#	1573.7	1573.75	-31.8	98	X	X
SPP-7	[26-38]	KR.TVGFGFNRNLHLY.KR	1536.7	1536.78	-52.1	99	X	X
SPP-7	[46-59]	KR.LIDPMTFGSGFANL.K#	1481.67	1481.72	-33.7	99	X	
SPP-8	[26-38]	KR.TMGFGFNRNMLLY.KR	1562.6	1562.74	-89.6	81	X	X
SPP-8	[46-59]	KR.LIDPMTFGSGFANL.R#	1481.67	1481.72	-33.7	99	X	
SPP-9	*[18-22]*	_*LAN.VCCGV.QKR_	*479.22*	*479.19*	*62.6*	*59*	*X*	
SPP-9	*[18-23]*	_*LAN.VCCGVQ.KR_	*607.21*	*607.25*	*-65.9*	*42*	*X*	*X*
SPP-9	[26-36]	KR.SLPYNPEYELY.KR	1386.61	1386.63	-14.4	96	X	
SPP-9	[44-57]	KR.LIDPLTFGSGFSNL.#	1479.62	1479.76	-94.6	99	X	X
SPP-10	[55-64]	KR.GAEFFLRRVVa.GKR	1191.61	1191.69	-67.1	70	X	

SPP-10	[55-65]	KR.GAEFFLRRVVG.KR	1249.65	1249.69	-32	93	X	
SPP-10	*[69-78]*	*KRS.TKPIDPNQYP.LVYGE#*	*1171.02*	*1171.59*	*-486.5*	*66*	*X*	
SPP-11	[24-28]	KR.YIRFG.KR	654.46	654.35	168.1			X
SPP-11	[31-37]	KR.HQQLFPN.KR	882.34	882.43	-102	85	X	X
SPP-11	[55-71]	KR.NDLLDSEFLNDMNENLE.KR	2023.97	2023.87	49.4	99	X	
SPP-12	[40-54]	KR.NYMDFFGLNGDMQRF.KK	1853.61	1853.79	-97.1	99	X	X
**SPP-13**	**[60-64](x2)**	**KK.FRGLLa.GKR**	**603.45**	**603.37**	**132.6**	**84**	**X**	**X**
SPP-15	[48-53](x8)	KR.FDPIQFa.GKR	764.35	764.39	-52.3	62	X	
SPP-15	[48-54](x8)	KR.FDPIQFG.KR	822.42	822.4	24.3			X
SPP-15	[120-125]	KR.FDPIMFa.GR#	767.4	767.37	39.1			X
SPP-16	[32-45]	KR.pQFDPIMYGKLRQFY.RR	1787.69	1787.87	-100.7	89	X	
SPP-16	*[33-38]*	*KRQ.FDPIMYa.GK* [§]	*783.32*	*783.35*	*-38.3*	*27*	*X*	
SPP-16	*[70-76]*	*KR.pQFDPIMY.KR*	*895.23*	*895.38*	*-167.5*	*84*	*X*	
SPP-16	*[70-76]*	KR.QFDPIMY.KR	912.49	912.41	87.7			X
SPP-16	[79-91]	KR.pQSNPYFLSDIRSI.KR	1521.67	1521.75	-52.6	99	X	X
SPP-16	[79-91]	KR.QSNPYFLSDIRSI.KR	1538.7	1538.7	0	83	X	X
SPP-16	*[80-91]*	*KRQ.SNPYFLSDIRSI.KR*	*1410.55*	*1410.71*	*-113.4*	*98*	*X*	
SPP-17	[51-64]	KK.IIDPMTYGTGFSNL.#	1527.6174	1527.73	-73.7	72	X	
SPP -18	[29-35]	KR.GYHFFRL.KK	938.45	938.48	-32	96	X	X
SPP -19	[30-39]	KR.KHIGHQIFRL.KR	1247.77	1247.73	32.1	73	X	
SPP -19	[34-39]	G.HQIFRL.KR	812.39	812.47	-98.5	30	X	
SPP -19	[42-48]	KR.GYHFFRL.RK	938.45	938.48	-32	96	X	X

[a] Period (.) indicates a cleavage site. Asterisk (*) indicates the presence of signal peptide before the sequence. Pound (#) indicates the end of a precursor sequence. (x number) indicates the number of repeated sequences in this prohormone. "X" indicates the instrument used to identify the peptide. Putative PTMs include N-terminal pyroglutamination ("p") and C-terminal amidation ("a"). **Bold** type indicates prohormone precursors characterized only in asexual animals by mass spectrometry. Underlined peptides are only detected in asexual animals, while the peptides in *italic* are identified as different forms (such as modified by PTM, etc.) compared to the ones in sexual animals. In addition, peptides detected with lower confidence are marked as §. Three prohormones labeled with Φ are tentative and not confirmed, since they do not meet the criteria for prohormone identification as described in experimental section.
