# Supplementary material for: Genome-Wide Analyses Reveal a Role for Peptide Hormones in Planarian Germline Development
Source: PLoS Biol. 2010 Oct 12;8(10):e1000509. doi: 10.1371/journal.pbio.1000509 (PMC2953531; doi:10.1371/journal.pbio.1000509)
Supplement: Table S4 — Peptide families encoded from S. mediterranea prohormone genes. (0.07 MB PDF) [file pbio.1000509.s009.pdf]

Table S4. Peptide families encoded from *S. mediterranea* prohormone genes

Prohormone Gene	Peptide Family
*mpl-1**	RL/RMamide
mpl-2	RL/RMamide
*npp-1**	RL/RMamide
*npp-22**	RL/RMamide
ppl-1	RL/RMamide
spp-1	RL/RMamide
spp-2	RL/RMamide
spp-3	RL/RMamide
spp-4	RL/RMamide
spp-5	RL/RMamide
ppp-1	Pedal Peptide
ppp-2	Pedal Peptide
*npp-3**	RFamide
*npp-4**	RFamide
spp-11	RFamide
*npp-18**	RRVVamide
spp-10	RRVVamide
*npp-2**	Miscellaneous amidated peptides
*npp-8**	Miscellaneous amidated peptides
*npp-12**	Miscellaneous amidated peptides
spp-13	Miscellaneous amidated peptides
spp-14	Miscellaneous amidated peptides
spp-15	Miscellaneous amidated peptides
cpp-1	Wamide
*npp-5**	Wamide
spp-12	Miscellaneous non-amidated peptides
spp-16	Miscellaneous non-amidated peptides

Prohormone Gene	Family
eye53-1	eye53
eye53-1	eye53
1020HH-1	1020HH
1020HH-1	1020HH
grh-1	FRL-containing
spp-18	FRL-containing
spp-19	FRL-containing
spp-6	Planarin
spp-7	Planarin
spp-8	Planarin
spp-9	Planarin
spp-17	Planarin
ilp-1	Insulin/IGF
*npy-1**	Neuropeptide Y Superfamily
npy-2	Neuropeptide Y Superfamily
npy-3	Neuropeptide Y Superfamily
*npy-4**	Neuropeptide Y Superfamily
npy-5	Neuropeptide Y Superfamily
npy-6	Neuropeptide Y Superfamily
npy-7	Neuropeptide Y Superfamily
npy-8	Neuropeptide Y Superfamily
*npy-9**	Neuropeptide Y Superfamily
npy-10	Neuropeptide Y Superfamily
npy-11	Neuropeptide Y Superfamily

*Genes previously predicted from the *S. mediterranea* genome (*6*)
