# Supplementary material for: Genome-Wide Analyses Reveal a Role for Peptide Hormones in Planarian Germline Development
Source: PLoS Biol. 2010 Oct 12;8(10):e1000509. doi: 10.1371/journal.pbio.1000509 (PMC2953531; doi:10.1371/journal.pbio.1000509)
Supplement: Table S5 — Sequence information for S. mediterranea prohormone genes. (0.25 MB PDF) [file pbio.1000509.s010.pdf]

Table S5. Sequence information for *S. mediterranea* prohormone genes (listed alphabetically)

**Gene Name:** *Smed-1020HH-1*	**Abbreviation:** *Smed-1020HH-1*
**Prohormone Sequence:** MNLKIFFILLIGIFVTCVTSYLSDGTSLENFDDLESNSDYMKRYSYLKGGIRWKKPNYRNNRYLKGGIRWKRRNYLKGGLRF	
**Signal Sequence:** MNLKIFFILLIGIFVTCVTS	
**Predicted Peptide(s):** YLSDGTSLENFDDLESNSDYM[Φ], YSYLKGGIRW[Φ], PNYRNNRYLKGGIRW, NYLKGGLRF	
**Prohormone Cloning Method:** 3' RACE	
**Oligonucleotide Primer(s):** TTTGAAGATGAATTTGAAAATTT (OUTER)	
**Asexual Expression Pattern:** CG, pharynx, VNC, ring of cells anterior to CG	
**Genbank accession:** GU295180	

**Gene Name:** *Smed-1020HH-2*	**Abbreviation:** *Smed-1020HH-2*
**Prohormone Sequence:** MIMKTVIVFFISVLLYGLLSISEAVPRSAENLRTYDSLLEELNDYDPIYKRQSYLTGGIRYKKQVPGKRYLTGGIRY	
**Signal Sequence:** MIMKTVIVFFISVLLYGLLSISEA	
**Predicted Peptide(s):** VPRSAENLRTYDSLLEELNDYDPIY, QSYLTGGIRY, pQSYLTGGIRY, QVPamide, QVPG, YLTGGIRY	
**Prohormone Cloning Method:** 3' RACE	
**Oligonucleotide Primer(s):** ATGATAATGAAAACTGTAATAGTA (OUTER), TTTTCATAAGCGTTCTCTTATACGG (INNER)	
**Asexual Expression Pattern:** CG (concentration in lateral region), VNCs, unidentified sub-epidermal cells, cells surrounding pharynx	
**Genbank accession:** BK007025	

**Gene Name:** *Smed-cerebral peptide prohormone Like-1*	**Abbreviation:** *Smed-cpp-1*
**Prohormone Sequence:** MNFTNRPWTCGTLILILSITFCVGLEGEDDTFVTFQEILRNGKPDLLSDNQDNQKVISFYKSKRVPGWGKRSYQWIKKTPGWGKRTYQWSKKTPGWG	
**Signal Sequence:** MNFTNRPWTCGTLILILSITFCVG	
**Predicted Peptide(s):** LEGEDDTFVTFQEILRNamide, LEGEDDTFVTFQEILRNG, PDLLSDNQDNQKVISFYKS, VPGWamide, VPGWG, SYQWI, TPGWamide (x2), TPGWG(x2), TYQWS	
**Prohormone Cloning Method:** 3' RACE	
**Oligonucleotide Primer(s):** TGAATTTTACGAATAGACCTTGGA (OUTER), CCTTGGACTTGTGGGACATT (INNER)	
**Asexual Expression Pattern:** CG (lateral cells), unidentified cells between VNCs in anterior half of animal	
**Genbank accession:** BK007012	

Gene Name: *Smed-eye53-1*	**Abbreviation:** *Smed-eye53-1*
**Prohormone Sequence:** MNLVPILTILCSLCLWLPRNADSMSMQKKLSIPTYWDDIDTSKRNAKRLSVPTYFDDWESRKKRSSAGKRLSVPPYWDEWESQRR	
**Signal Sequence:** MNLVPILTILCSLCLWLPRNADS	

Predicted Peptide(s): MSMQ, LSIPTYWDDIDTS, NA, LSVPTYFDDWESR, SSAamide, SSAG, LSVPPYWDEWESQ

Prohormone Cloning Method: EST PL05009A1G02, Contig6690

Oligonucleotide Primer(s): N/A

Asexual Expression Pattern: CG, VNC, photoreceptors, small cells near anterior margin, pharynx, lateral stripes of cells (ventral)

Genbank accession: BK007033

**Gene Name:** *Smed-eye53-2*	**Abbreviation:** *Smed-eye53-2*

Prohormone Sequence: MIIALVSAVIIGLTQGEYRSSKNSGRNDWSLLKSSHIKRALVPDAWDDWEIKRSEHKKRAVVPDAWDDWEIKRAI

Signal Sequence: MIIALVSAVIIGLTQG

Predicted Peptide(s): EYRSSKNSGRNDWSLLKSSHI, ALVPDAWDDWEI, SEH, AVVPDAWDDWEI$^{\Phi}$, AI

Prohormone Cloning Method: 3' RACE

Oligonucleotide Primer(s): GCGTGATGATAATAGCATTGG (Outer), AATAGCATTGGTATCAGCAGTGA(Inner)

Asexual Expression Pattern: CG, VNC, unidentified sub-epidermal cells, pharynx, photoreceptors

Genbank accession: BK007024

**Gene Name:** *Smed-gonadotropin releasing hormone like-1*	**Abbreviation:** *Smed-grh-1*

Prohormone Sequence: MTKFVRFFIILFIFLQIVSEICPQSYHFSNDWLPGKRYHTGHQMFRLKKGFHYFRLKKPSNCIFDNYFKQDILKLARKHSGLCPSDLSFIEELQNIK

Signal Sequence: MTKFVRFFIILFIFLQIVS or MTKFVRFFIILFIFLQIVSEICP

Predicted Peptide(s): EICPQSYHFSNDWLPamide, EICPQSYHFSNDWLPG, YHTGHQMFRL, GFHYFRL, PSNCIFDNYFKQDILKLARKHSGLCPSDLSFIEELQNIK

Prohormone Cloning Method: 3' RACE

Oligonucleotide Primer(s): (Outer)

Asexual Expression Pattern: CG, VNC, pharynx

Genbank accession: GU295185

**Gene Name:** *Smed-insulin like prohormone-1*	**Abbreviation:** *Smed-ilp-1*

Prohormone Sequence: MLKMYFAFYLVVLYIQFYSGEIFKYELYNQSQADLERNLEVRFCQHRLLKAITTLCNNVNINYLRHFFANRTNIMHPIYKYVIRPELLSMASIGRYYSPESINCNAYKKSLVDECCCKSCTMLNLFKYCPSDDEARSLKFIK

Signal Sequence: MLKMYFAFYLVVLYIQFYSG

Predicted Peptide(s): EIFKYELYNQSQADLERNLEVRFCQHRLL$^{\Phi}$, AITTLCNNVNINYLRHFFANRTNIMHPIYKYVIRPELLSMASIGRYYSPESINCNAY$^{\Phi}$, SLVDECCCKSCTMLNLFKYCPSDDEARSL, FIK

Prohormone Cloning Method: EST PL05016A1H10, Contig3473

Oligonucleotide Primer(s): N/A

Asexual Expression Pattern: CG, VNC

Genbank accession: BK007034

**Gene Name**: *Smed-myomodulin prohormone like-1*	**Abbreviation:** *Smed-mpl-1*
**Prohormone Sequence:** MSKLTYFIFMIMLFIFVQTIDINSNQYEEDYDPNDDHELDKRAYRLMRMGKRAVRLMRMGKKAVRLMRLGKRSDMA	
**Signal Sequence:** MSKLTYFIFMIMLFIFVQT	
**Predicted Peptide(s):** IDINSNQYEEDYDPNDDHELD, AYRLMRMamide, AYRLMRMG, AVRLMRMamide, AVRLMRMG, AVRLMRLamide, AVRLMRLG, SDMA	
**Prohormone Cloning Method:** Amplified from cDNA	
**Oligonucleotide Primer(s):** TTTTCATGATAATGCTCTTCATTTT (Forward), TGCCATATCGCTTCTTTTACC (Reverse)	
**Asexual Expression Pattern:** CG, VNCs, cells surrounding pharynx	
**Genbank accession:** BK007017	

**Gene Name:** *Smed-myomodulin prohormone like-2*	**Abbreviation:** *Smed-mpl-2*
**Prohormone Sequence:** MQYLFAAFIFMAYYIRCEDFNENIIYPELFEDSEQYNAIPWNKRAVRLMRLGKRIAPLKRAVKLMRLGKREE	
**Signal Sequence:** MQYLFAAFIFMAYYIRC	
**Predicted Peptide(s):** EDFNENIIYPELFEDSEQYNAIPWN, AVRLMRLamide, AVRLMRLG, IAPL, AVKLMRLamide, AVKLMRLG[§], EE	
**Prohormone Cloning Method:** Amplified from cDNA	
**Oligonucleotide Primer(s):** TGCAATATTTATTTGCAGCTTTC (Forward), CGTTAAATAAAATTCGATTATTCCTC (Reverse)	
**Asexual Expression Pattern:** CG, PR, pharynx (at base and within), VNCs, marginal adhesive gland cells	
**Genbank accession:** BK007016	

**Gene Name:** *Smed-neuropeptide precursor-1*	**Abbreviation:** *Smed-npp-1*
**Prohormone Sequence:** MKLEIRIVVVTVLLSAVLADYYNSDDSDSKKRSFVRLGKRASFVRLGRSGYEWQSPFVKKASFVRLGKKSRLDYEPVDDYNKRASFVRLGRTYE	
**Signal Sequence:** MKLEIRIVVVTVLLSAVLA	
**Predicted Peptide(s):** DYYNSDDSDS, SFVRLamide, SFVRLG, ASFVRLamide (x3), ASFVRLG (x3), SGYEWQSPFV, SRLDYEPVDDYN, TYE	
**Prohormone Cloning Method:** EST PL04023B1E01, Contig3553	
**Oligonucleotide Primer(s):** N/A	
**Asexual Expression Pattern:** pharynx	
**Genbank accession:** BK007036	

**Gene Name:** *Smed-neuropeptide precursor-2*	**Abbreviation:** *Smed-npp-2*
**Prohormone Sequence:** MIFQFLLLLFVTTVWTDSDSDCDICEICEAGGLMDSTECCLSSVLYKICESKLEISRDKRRGLIGKRRGLIGKRRGLIGKRRGLIGKRRGLIGKRRGLIGKRRGLIGKRQLLNSKQSIFQDEY	
**Signal Sequence:** MIFQFLLLLFVTTVWT	
**Predicted Peptide(s):** DSDSDCDICEICEAGGLMDSTECCLSSVLYKICESKLEISRD, RGLIamide or GLIamide (x7), RGLIG or GLIG (x7), QLLNSKQSIFQDEY, pQLLNSKQSIFQDEY	

**Prohormone Cloning Method:** EST PL05001B1G10, Contig194	
**Oligonucleotide Primer(s):** N/A	
**Asexual Expression Pattern:** CG, VNC, pharynx, unidentified sub-epidermal cells	
**Genbank accession:** BK007035	

**Gene Name:** *Smed-neuropeptide precursor-3*	**Abbreviation:** *Smed-npp-3*
**Prohormone Sequence:** MMAKQFPYFIINLSIVLCLCFYIQFADQMPDQYENRYELLDPISKHELNYQTGKRAILLTRYGKRGS RRYFLNNDDSKRAIVLTRFG	
**Signal Sequence:** MMAKQFPYFIINLSIVLCLCFYIQFADQ	
**Predicted Peptide(s):** MPDQYENRYELLDPISKHELNYQTamide, MPDQYENRYELLDPISKHELNYQTG, AILLTRYamide, AILLTRYG, GS,YFLNNDDS, GSRRYFLNNDDS, AIVLTRFamide, AIVLTRFG	
**Prohormone Cloning Method:** EST PL06010A1H10, Contig7559	
**Oligonucleotide Primer(s):** N/A	
**Asexual Expression Pattern:** CG, VNC, pharynx, unidentified sub-epidermal cells at lateral margin	
**Genbank accession:** GU295187	

**Gene Name:** *Smed-neuropeptide precursor-4*	**Abbreviation:** *Smed-npp-4*
**Prohormone Sequence:** MNSLSITIFVLSILVSWAQSEQKRSSVFRFGKRDQSIDQLTDDSAFSNVPVESTRRNYEQLLSPYP ENDQDENKRSVAFRFGKRRGVAFRFGKRGSVFRYGKRQSVFRYG	
**Signal Sequence:** MNSLSITIFVLSILVSWAQS	
**Predicted Peptide(s):** EQ, SSVFRFamide, SSVFRFG, DQSIDQLTDDSAFSNVPVEST, NYEQLLSPYPENDQDEN, SVAFRFamide, SVAFRFG, RGVAFRFamide, RGVAFRFG, GSVFRYamide, GSVFRYG, QSVFRYamide, QSVFRYG,	
**Prohormone Cloning Method:** EST Pl08007a2b03, Contig7622	
**Oligonucleotide Primer(s):** N/A	
**Asexual Expression Pattern:** CG, pharynx, unidentified sub-epidermal cells	
**Genbank accession:** BK007037	

**Gene Name:** *Smed-neuropeptide precursor-5*	**Abbreviation:** *Smed-npp-5*
**Prohormone Sequence:** MNLFLLIGVIIVGVQVTVDAENSDLIDALKKRPNWKDMPWGKRSAWRDMPWGKRSAWRDMPWG KRSAWRDMPWGKRSAWRDMPWGKKSAWRDMPWGKRSDWKDISWEKKQGLHDLSLNNPFEK SLILNNSEFED	
**Signal Sequence:** MNLFLLIGVIIVGVQVTVDA	
**Predicted Peptide(s):** ENSDLIDAL, PNWKDMPWamide, PNWKDMPWG, SAWRDMPWamide(x5), SAWRDMPG(x5), SDWKDISWE, QGLHDLSLNNPFEKSLILNNSEFED	
**Prohormone Cloning Method:** 3’ RACE	
**Oligonucleotide Primer(s):** CGGAGTTATTATTGTTGGCGTTC(OUTER), AAAAAGGCCAAATTGGAAGG(INNER)	
**Asexual Expression Pattern:** CG, cells surrounding ventral midline	
**Genbank accession:** BK007015	

**Gene Name:** *Smed-neuropeptide precursor-8*	**Abbreviation:** *Smed-npp-8*
**Prohormone Sequence:** MKLVFMLLFSLNILILSNRWADGMMQDYLDRKLFLASKRLADKKDPRFSDQVWHSGYGKRNYYNRFDGQAWYSGYGKRNDAFDGQAWYSGYGR	
**Signal Sequence:** MKLVFMLLFSLNILILSNRWADG	
**Predicted Peptide(s):** MMQDYLDRKLFLAS, LAD, DPRFSDQVWHSGYamide, DPRFSDQVWHSGYG, NYYNRFDGQAWYSGYamide, NYYNRFDGQAWYSGYG, NDAFDGQAWYSGYamide, NDAFDGQAWYSGY	
**Prohormone Cloning Method:** 3' RACE	
**Oligonucleotide Primer(s):** AAATTCCAAATAAACACCTGACG (OUTER), TTCTAGCATCTAAAAGATTAGCAGACA (INNER)	
**Asexual Expression Pattern:** CG, VNC, pharynx (tip), intestine, unidentified sub-epidermal cells at lateral margin	
**Genbank accession:** GU295189	

**Gene Name:** *Smed-neuropeptide precursor-12*	**Abbreviation:** *Smed-npp-12*
**Prohormone Sequence:** MKLILLFILLLVCIVHSRYFQPQQDFPKSNIFQLPESEFENDDVIQKRNYFGKRNYFGKRNYFGKRNYFWKKKLFRLKIQTIN	
**Signal Sequence:** MKLILLFILLLVCIVHS	
**Predicted Peptide(s):** RYFQPQQDFPKSNIFQLPESEFENDDVIQ, NYFamide(x3), NYFG(x3), NYFW, LFRLKIQTIN	
**Prohormone Cloning Method:** 3' RACE	
**Oligonucleotide Primer(s):** CAGATTTGTTTTAACAATGAAATTAA (OUTER)	
**Asexual Expression Pattern:** CG, cells near or in VNC, pharynx, cells at anterior margin, Photoreceptors	
**Genbank accession:** GU295182	

**Gene Name:** *Smed-neuropeptide precursor-18*	**Abbreviation:** *Smed-npp-18*
**Prohormone Sequence:** MELFSRTTVFFLIIFPIWMMSVILIEARNMDLDEYDSLLPKDKRGAEFFIRRVVGKRGAEFFIRRVVGKRNSDYLIQ	
**Signal Sequence:** MELFSRTTVFFLIIFPIWMMSVILIEA	
**Predicted Peptide(s):** RNMDLDEYDSLLPKD, GAEFFIRRVVamide (x2), GAEFFIRRVVG(x2), NSDYLIQ	
**Prohormone Cloning Method:** 3' RACE	
**Oligonucleotide Primer(s):** TGGAATTTATCAGTAGAACTACAGTA (OUTER)	
**Asexual Expression Pattern:** unidentified sub-epidermal cells	
**Genbank accession:** BK007027	

**Gene Name:** *Smed-neuropeptide precursor-22*	**Abbreviation:** *Smed-npp-22*
**Prohormone Sequence:** MKTLSLCLSIGFSLVLIASLPFVSSTTDDEVNENICQSLCHKSLSCLDECHDISESNDSMEKRAKYFRLGKRAKYFRLGKRSFDSSNLEKRAKYFRLG	
**Signal Sequence:** MKTLSLCLSIGFSLVLIASLPFVSS	
**Predicted Peptide(s):** TTDDEVNENICQSLCHKSLSCLDECHDISESNDSME, AKYFRLamide(x3), AKYFRLG(x3), SFDSSNLE	

**Prohormone Cloning Method:** EST PL05004B1G05, Contig1226
**Oligonucleotide Primer(s):** N/A
**Asexual Expression Pattern:** CG, VNC, pharynx, unidentified sub-epidermal cells, anterior concentration of cells outside CG
**Genbank accession:** BK007038

**Gene Name:** *Smed-neuropeptide y superfamily-1*	**Abbreviation:** *Smed-npy-1*
**Prohormone Sequence:** MTFIYGFLCLTLVNVICSQKSLFIEPPAKPEFFDDPELLRNYIKKLNEYFAIVGRPRFGKRFDRGFS	
**Signal Sequence:** MTFIYGFLCLTLVNVICS	
**Predicted Peptide(s):** QKSLFIEPPAKPEFFDDPELLRNYI, pEPPAKPEFFDDPELLRNYI, LNEYFAIVGRPRFamide[§], LNEYFAIVGRPRFG, FDRGFS	
**Prohormone Cloning Method:** 3' RACE	
**Oligonucleotide Primer(s):** ATGACTTTCATTTACGGATTTCTATG	
**Asexual Expression Pattern:** CG, VNC	
**Genbank accession:** GU295175	

**Gene Name:** *Smed-neuropeptide y superfamily-2*	**Abbreviation:** *Smed-npy-2*
**Prohormone Sequence:** MNFPIISIVALLTVFNCFSAMEDDTKSLAELKNLLSDLNEEYLIAGRPRFGKRNMIFKRSANPLKWMTL	
**Signal Sequence:** MNFPIISIVALLTVFNCFS	
**Predicted Peptide(s):** AMEDDTKSLAELKNLLSDLNEEYLIAGRPRFamide, AMEDDTKSLAELKNLLSDLNEEYLIAGRPRFG, NMIF, SANPLKWMTL	
**Prohormone Cloning Method:** 3' RACE	
**Oligonucleotide Primer(s):** ATGAATTTCCCGATTATTTCTATTG	
**Asexual Expression Pattern:** CG, VNC	
**Genbank accession:** BK007019	

**Gene Name:** *Smed-neuropeptide y superfamily-3*	**Abbreviation:** *Smed-npy-3*
**Prohormone Sequence:** MSTKEFTFCFVMICALFCQIVQISANKDELDILFKKRNNNQLDDPDIQQYLQDLNNFYQFYGRPRFGKRQKFHRD	
**Signal Sequence:** MSTKEFTFCFVMICALFCQIVQISA	
**Predicted Peptide(s):** NKDELDILF, NNNQLDDPDIQQYLQDLNNFYQFYGRPRFamide, NNNQLDDPDIQQYLQDLNNFYQFYGRPRFG, QKFHRD, pQKFHRD	
**Prohormone Cloning Method:** Amplified from cDNA	
**Oligonucleotide Primer(s):** CATTATTTTGCCAAATCGTTCA (Forward), CTGGATAAAGTAATATCATTTATTTAA (Reverse)	
**Asexual Expression Pattern:** CG, VNC, unidentified sub-epidermal cells, pharynx, cells surrounding pharynx	
**Genbank accession:** BK007009	

**Gene Name:** *Smed-neuropeptide y superfamily-4*	**Abbreviation:** *Smed-npy-4*
**Prohormone Sequence:** MKFCCAPRLSVLFLAVFIFGISLTAKEVKRKVVYLKSRNHFRSDEDYVSYLRKVQKYIQLYGRPRFGKRQTNWYDMKNYEGNENYDSYTF	

**Signal Sequence:** MKFCCAPRLSVLFLAVFIFGISLTA
**Predicted Peptide(s):** KEV, VVYLKSRNHFRSDEDYVSYLRKVQKYIQLYGRPRFamide, VVYLKSRNHFRSDEDYVSYLRKVQKYIQLYGRPRFG, QTNWYDMKNYEGNENYDSYTF
**Prohormone Cloning Method:** EST PL05016B2E03, Contig3649
**Oligonucleotide Primer(s):** N/A
**Asexual Expression Pattern:** unidentified sub-epidermal cells (ventral)
**Genbank accession:** BK007039

**Gene Name:** *Smed-neuropeptide y superfamily-5*	**Abbreviation:** *Smed-npy-5*
**Prohormone Sequence:** MLIGYNCKSGIILLMAVLGAMWLSGIQTDDRVDIRKSIFSSPEALRRYLLQMNEYLAIVARPRYGKRSLPIDMNRLQFDNYENYFK	
**Signal Sequence:** MLIGYNCKSGIILLMAVLGAMWLSGIQT	
**Predicted Peptide(s):** DDRVDIRKSIFSSPEAL, YLLQMNEYLAIVARPRYamide[Φ], YLLQMNEYLAIVARPRYG[Φ], SLPIDMNRLQFDNYENYF	
**Prohormone Cloning Method:** 3' RACE	
**Oligonucleotide Primer(s):** TTGATCGGGTATAACTGTAAAAGC	
**Asexual Expression Pattern:** CG, VNC, pharynx (distal region), lateral stripes of cells (ventral)	
**Genbank accession:** BK007008	

**Gene Name:** *Smed-neuropeptide y superfamily-6*	**Abbreviation:** *Smed-npy-6*
**Prohormone Sequence:** MYGFSHSALKIILILISISSLILSTFSSDPVIDFDLEKDKDLMKYMQDLNNYSQLYGRPRFGKRSLNSFQTIREFVRDRRLW	
**Signal Sequence:** MYGFSHSALKIILILISISSL	
**Predicted Peptide(s):** ILSTFSSDPVIDFDLEKDKDLMKYMQDLNNYSQLYGRPRFamide, ILSTFSSDPVIDFDLEKDKDLMKYMQDLNNYSQLYGRPRFG, SLNSFQTIREFVRD, LW	
**Prohormone Cloning Method:** 3' RACE	
**Oligonucleotide Primer(s):** TATGGGTTTTCGCATTCTGC	
**Asexual Expression Pattern:** CG, cells anterior to CG, pharynx (inner ring), VNCs	
**Genbank accession:** BK007031	

**Gene Name:** *Smed-neuropeptide y superfamily-7*	**Abbreviation:** *Smed-npy-7*
**Prohormone Sequence:** MFERIRNSIFLLFISLNWCEAQYPIFGKMLDSIPFKSRTPIAGIVNKMGQIRKLSEREIKLLVYLLNEHFAIYGRPRYG	
**Signal Sequence:** MFERIRNSIFLLFISLNWCEA	
**Predicted Peptide(s):** QYPIFGKMLDSIPFKSRTPIAGIVNKMGQIRKLSEREI[§], LLVYLLNEHFAIYGRPRYamide, LLVYLLNEHFAIYGRPRYG	
**Prohormone Cloning Method:** 3' RACE	
**Oligonucleotide Primer(s):** GTTTGAGAGAATTAGGAATTCG (OUTER), AGGAATTCGATATTTCTTCTTTTCA (INNER)	
**Asexual Expression Pattern:** CG, VNC, pharynx, unidentified Sub-epidermal cells	
**Genbank accession:** BK007020	

Gene Name: *Smed-neuropeptide y superfamily-8* | **Abbreviation:** *Smed-npy-8*

Prohormone Sequence:
MIINKCYFLVFILCFMSFIHLNTCNQKRPMFDSADAFRNYLRKLNNEYMIAGRPRFGKRRSDFEKELFYNLKSNL

Signal Sequence: MIINKCYFLVFILCFMSFIHLNTC

Predicted Peptide(s): PMFDSADAFRNYLRKLNNEYMIAGRPRFamide, PMFDSADAFRNYLRKLNNEYMIAGRPRFG, RSDFEKELFYNLKSNL

Prohormone Cloning Method: 3' RACE

Oligonucleotide Primer(s):
TCATTAACAAATGCTATTTTCTCG (OUTER), CAATCAGAAAAGGCCGATGT (INNER)

Asexual Expression Pattern: None

Genbank accession: BK007010

Gene Name: *Smed-neuropeptide y superfamily-9* | **Abbreviation:** *Smed-npy-9*

Prohormone Sequence:
MYFLQKFFILSISLTLCVICFNRNVSGLTKQKYSLFSGPEDLRNYLRQLNEYIALSSRPRYGKRNEWTSISE

Signal Sequence: MYFLQKFFILSISLTLCVICFNRNVSG

Predicted Peptide(s): LTKQKYSLFSGPEDLRNYLRQLNEYIALSSRPRYamide, LTKQKYSLFSGPEDLRNYLRQLNEYIALSSRPRYG, NEWTSISE, pEWTSISE[§]

Prohormone Cloning Method: EST PL04007A1D04

Oligonucleotide Primer(s): N/A

Asexual Expression Pattern: CG, VNC, unidentified sub-epidermal cells, pharynx

Genbank accession: BK007040

Gene Name: *Smed-neuropeptide y superfamily-10* | **Abbreviation:** *Smed-npy-10*

Prohormone Sequence:
MNISRFLKVFVIFILFQNICTLSDYETSSFNQWYDKRDLKPLFNNAKQLLWYLQKLDKMYAIAGRPRYGKR

Signal Sequence: MNISRFLKVFVIFILFQNICT

Predicted Peptide(s): LSDYETSSFNQWYD,
DLKPLFNNAKQLLWYLQKLDKMYAIAGRPRYamide,
DLKPLFNNAKQLLWYLQKLDKMYAIAGRPRYG

Prohormone Cloning Method: 3' RACE

Oligonucleotide Primer(s):
CAAAATATTTGCACGTTGAGTGA (OUTER), TGCACGTTGAGTGATTATGAAA (INNER)

Asexual Expression Pattern: CG, VNC, intestine

Genbank accession: BK007011

Gene Name: *Smed-neuropeptide y superfamily-11* | **Abbreviation:** *Smed-npy-11*

Prohormone Sequence:
MLIPCLLGALIFAIQDSHCYFIMPSNSMDPNKPPIPLNSHATSDEIKDYLHHLNLYFQIVSRPRLGKRQKYDFHDILNQF

Signal Sequence: MLIPCLLGALIFAIQDSHC

Predicted Peptide(s): YFIMPSNSMDPNKPPIPLNSHATSDEIKDYLHHLNLYFQIVSRPRLamide, YFIMPSNSMDPNKPPIPLNSHATSDEIKDYLHHLNLYFQIVSRPRLG, QKYDFHDILNQF

**Prohormone Cloning Method:** 3' RACE	
**Oligonucleotide Primer(s):** TGTTGATTCCATGCTTATTAGGTG (OUTER), TTCCATGCTTATTAGGTGCATT (INNER)	
**Asexual Expression Pattern:** pharynx (distal tip)	
**Genbank accession:** BK007021	

**Gene Name:** *Smed-pyrokinin prohormone like-1*	**Abbreviation:** *Smed-ppl-1*
**Prohormone Sequence:** MKFSRICFIILVSLELFVVNEAVHMSDQLVFSLLVLIANHEINSKNFIYIPDDSFINEMDRAIDKKIFSP RMGKRYFSPRMGKRYFSPRMGKRYFSPRMGKRYFSPRLGK	
**Signal Sequence:** MKFSRICFIILVSLELFVVNEA	
**Predicted Peptide(s):** VHMSDQLVFSLLVLIANHEINSKNFIYIPDDSFINEMDRAID, IFSPRMamide, IFSPRMG, YFSPRMamide (x3), YFSPRMG(x3), YFSPRLamide, YFSPRLG	
**Prohormone Cloning Method:** Amplifcation from cDNA	
**Oligonucleotide Primer(s):** TGAAATTCAGCAGGATTTGTTT (Forward), TTATGAAGCACTTTATGTTCATTTACC (Reverse)	
**Asexual Expression Pattern:** CG, pharynx(tip), occasional cells labeled near VNC's	
**Genbank accession:** BK007007	

**Gene Name:** *Smed-pedal peptide prohormone like-1*	**Abbreviation:** *Smed-ppp-1*
**Prohormone Sequence:** MKSTGLLILTFVLVSVEFGDFYRVSSSDLRRFKKYSYYDSIGSGLLKRGAYYDPIGGGLLKRSSYY DPIGGGLLKRDSNYDPIGGGLLKRRSFYDPIGGGLLKRRSFYDPIGGGLLKRRSFYDPIGGGLLKK RFYNDPLGVALLKSRFDKDSIN	
**Signal Sequence:** MKSTGLLILTFVLVSVEFG	
**Predicted Peptide(s):** DFYRVSSSDLRRF, YSYYDSIGSGLL, GAYYDPIGGGLL, SSYYDPIGGGLL, DSNYDPIGGGLL, RSFYDPIGGGLL (x3), RFYNDPLGVALLKSRFDKDSIN	
**Prohormone Cloning Method:** EST PL05001A2A04, Contig128	
**Oligonucleotide Primer(s):** N/A	
**Asexual Expression Pattern:** CG, VNC, pharynx (inner ring), unidentified sub-epidermal cells	
**Genbank accession:** BK007041	

**Gene Name:** *Smed-pedal peptide prohormone like-2*	**Abbreviation:** *Smed-ppp-2*
**Prohormone Sequence:** MTVNGILISSMMITIILSTVDLSKSREHGEIKRFRYFDKIGSDLLKRSYFDKIGNDLLKRSYMDKIGSD LLKRRYFDKIGSEMLKRSYMDKIGSDL	
**Signal Sequence:** MTVNGILISSMMITIILS	
**Predicted Peptide(s):** TVDLSKSREHGEI, FRYFDKIGSDLL, SYFDKIGNDLL, SYMDKIGSDLL, RYFDKIGSEML, SYMDKIGSDL	
**Prohormone Cloning Method:** Amplified from cDNA	
**Oligonucleotide Primer(s):** TATAATGACAGTCAACGGTATTTTGAT (F), GATTTCTACAACAAATCGCTTCC (R)	
**Asexual Expression Pattern:** CG, VNC, pharynx, unidentified sub-epidermal cells	
**Genbank accession:** BK007018	

**Gene Name:** *Smed-secreted peptide prohormone-1*	**Abbreviation:** *Smed-spp-1*

Prohormone Sequence (two splice variants):
SMED-SPP-1A
MTMNKLTILFSLFLSVFVARSNASAFSSPGNSGAMILLTSGCLFMNTFAEDLGSLNADIDLDDSRLDKKAYWASRMGKRAYWASRMGKKAYWASRMGK
SMED-SPP-1B
MILLTSGCLFMNTFAEDLGSLNADIDLDDSRLDKKAYWASRMGKRAYWASRMGKKAYWASRMGK

Signal Sequence: MTMNKLTILFSLFLSVFVARSNA (**SMED-SPP-1A**)
MILLTSGCLFMNTFA (**SMED-SPP-1B**)

Predicted Peptide(s): SAFSSPGNSGAMILLTSGCLFMNTFAEDLGSLNADIDLDDSRLD, EDLGSLNADIDLDDSRLD, AYWASRMamide (x3), AYWASRMG (x3)

Prohormone Cloning Method: Amplified from cDNA

Oligonucleotide Primer(s): GACAGAAAAATATGACAATGAACAA (F), TCATATTAATCAAAACGCAAATAAACA (R)

Asexual Expression Pattern: CG, VNC, pharynx

Genbank accession: GU295176

**Gene Name:** *Smed-secreted peptide prohormone-2*	**Abbreviation:** *Smed-spp-2*

Prohormone Sequence:
MAIKILYSFLSILFCISVQGEFYQNFPADSNPCDELCEPQNLCDKLCSSYQNSDFEHEDAKRAVFLRLGRNIKRAPFLRLGRSQQKKSKFLRLGK

Signal Sequence: MAIKILYSFLSILFCISVQG

Predicted Peptide(s): EFYQNFPADSNPCDELCEPQNLCDKLCSSYQNSDFEHEDA, AVFLRLamide, AVFLRLG, NI, APFLRLamide, APFLRLG, SQQ, SKFLRLamide, SKFLRLG

Prohormone Cloning Method: 3' RACE

Oligonucleotide Primer(s): GGCAATCAAAATTTTGTATTCTTTTC

Asexual Expression Pattern: CG (concentration of cells in medial domain)

Genbank accession: BK007032

**Gene Name:** *Smed-secreted peptide prohormone-3*	**Abbreviation:** *Smed-spp-3*

Prohormone Sequence:
MYYFKIILLISFICIQQEFIFSSVMDDLSKDETYLSKRRYSLINPRLGKRYLINPRLGKRFQIKDIENLD

Signal Sequence: MYYFKIILLISFICIQQEFIFS

Predicted Peptide(s): SVMDDLSKDETYLS, RYSLINPRLamide, RYSLINPRLG, YLINPRLamide, YLINPRLG, FQIKDIENLD

Prohormone Cloning Method: 3' RACE

Oligonucleotide Primer(s): GTATTACTTTAAAATTATATTACTAATAAG

Asexual Expression Pattern: CG (concentration of cells in medial and lateral regions of CG), VNC, pharynx

Genbank accession: BK007022

**Gene Name:** *Smed-secreted peptide prohormone-4*	**Abbreviation:** *Smed-spp-4*

Prohormone Sequence:
MFYKFILLSVIMIFITKIEADYSSLNDDSELEDSYHRYPSSIKRGLRLMRLGKRNMNDEFQFRDLKKRGLRLMRLG

Signal Sequence: MFYKFILLSVIMIFITKIEA

Predicted Peptide(s): DYSSLNDDSELEDSYHRYPSSI, GLRLMRLamide (x2), GLRLMRLG (x2),

NMNDEFQFRDL
**Prohormone Cloning Method:** 3' RACE
**Oligonucleotide Primer(s):** CATCTAACATGTTTTACAAATTTATT
**Asexual Expression Pattern:** unidentified sub-epidermal cells, CG, Pharynx
**Genbank accession:** GU295179

**Gene Name:** *Smed-secreted peptide prohormone-5*	**Abbreviation:** *Smed-spp-5*
**Prohormone Sequence:** MKVLLFIVLCVVFVFALDDPEDSLNYYSNYSPAAERSELVSKRGLRILRMGKRNDLFRLLDKRGMRHMRLG	
**Signal Sequence:** MKVLLFIVLCVVFVFA	
**Predicted Peptide(s):** LDDPEDSLNYYSNYSPAAERSELVS, GLRILRMamide, GLRILRMG, NDLFRLLD, GMRHMRLamide, GMRHMRLG	
**Prohormone Cloning Method:** 3' RACE	
**Oligonucleotide Primer(s):** TTTTTCCCAAGACAAAATGAAA (OUTER), CCCAAGACAAAATGAAAGTGC (INNER)	
**Asexual Expression Pattern:** CG, VNC, Pharynx, unidentified sub-epidermal cells	
**Genbank accession:** GU295188	

**Gene Name:** *Smed-secreted peptide prohormone-6*	**Abbreviation:** *Smed-spp-6*
**Prohormone Sequence:** MIKIRILMSVLLFMAICLAAGLAEIDKRIPGIGFNRNFAIYKRMLEKRLIDPMTFGYGFSNLK	
**Signal Sequence:** MIKIRILMSVLLFMAICLAAGLA	
**Predicted Peptide(s):** EID, IPGIGFNRNFAIY, MLE, LIDPMTFGYGFSNL	
**Prohormone Cloning Method:** 3' RACE	
**Oligonucleotide Primer(s):** ATCAACATGATCAAGATCAGAATTT	
**Asexual Expression Pattern:** unidentified cells between VNC in anterior half of animals, cells surrounding pharynx, cells at medial CG	
**Genbank accession:** GU295177	

**Gene Name:** *Smed-secreted peptide prohormone-7*	**Abbreviation:** *Smed-spp-7*
**Prohormone Sequence:** MKFIISLFVVLFLCVVMAMSEIDKRTVGFGFNRNLHLYKRMLEKRLIDPMTFGSGFANLK	
**Signal Sequence:** MKFIISLFVVLFLCVVMAMS	
**Predicted Peptide(s):** EID, TVGFGFNRNLHLY, MLE, LIDPMTFGSGFANL	
**Prohormone Cloning Method:** 3' RACE	
**Oligonucleotide Primer(s):** ATGAAATTTATCATTTCCTTATTTGTC	
**Asexual Expression Pattern:** unidentified cells between VNC in anterior half of animals, cells surrounding pharynx	
**Genbank accession:** GU295178	

**Gene Name:** *Smed-secreted peptide prohormone-8*	**Abbreviation:** *Smed-spp-8*
**Prohormone Sequence:** MKIIILLAIILAVCAFEALGEVEKRTMGFGFNRNMLLYKRMLEKRLIDPMTFGSGFANLR	
**Signal Sequence:** MKIIILLAIILAVCAFEALG	
**Predicted Peptide(s):** EVE, TMGFGFNRNMLLY, MLE, LIDPMTFGSGFANL	

**Prohormone Cloning Method:** 3' RACE
**Oligonucleotide Primer(s):** CATGAAAATTATTATTTTACTAGCTAT
**Asexual Expression Pattern:** unidentified cells between VNC in anterior half of animals, cells surrounding pharynx
**Genbank accession:** GU295181

**Gene Name:** *Smed-secreted peptide prohormone-9*	**Abbreviation:** *Smed-spp-9*
**Prohormone Sequence:** MNSLIILLIVALVCLANVCCGVQKRSLPYNPEYELYKRFVEKRLIDPLTFGSGFSNL	
**Signal Sequence:** MNSLIILLIVALVC	
**Predicted Peptide(s):** LANVCCGVQ§, SLPYNPEYELY, FVE, LIDPLTFGSGFSNL	
**Prohormone Cloning Method:** 3' RACE	
**Oligonucleotide Primer(s):** TGAATTCGTTGATTATTTTACTTATCG	
**Asexual Expression Pattern:** unidentified cells between VNC in anterior half of animals, cells surrounding pharynx, cells scattered in CG	
**Genbank accession:** BK007026	

**Gene Name:** *Smed-secreted peptide prohormone-10*	**Abbreviation:** *Smed-spp-10*
**Prohormone Sequence:** MNFQSKIIIMIMCHFVITVFNEPLSKYYPDNNEDLSATIKRGAEFFLQRVEGKRGAEFFLRRVVGKR STKPIDPNQYPLVYGE	
**Signal Sequence:** MNFQSKIIIMIMCHFVITVFN	
**Predicted Peptide(s):** EPLSKYYPDNNEDLSATI, GAEFFLQRVEamide, GAEFFLQRVEG, GAEFFLRRVVamide, GAEFFLRRVVG, STKPIDPNQYPLVYGE	
**Prohormone Cloning Method:** 3' RACE	
**Oligonucleotide Primer(s):** GATAATGTGCCACTTTGTTATAACAG	
**Asexual Expression Pattern:** CG, and unidentified cells between VNC in anterior half of animal	
**Genbank accession:** BK007028	

**Gene Name:** *Smed-secreted peptide prohormone-11*	**Abbreviation:** *Smed-spp-11*
**Prohormone Sequence:** MKLLFVAALFLVIMDVYAIQEKRYIRFGKRHQQLFPNKRYIRFGKREYIPLDKRNDLLDSEFLNDMN ENLEKRYIRFGR	
**Signal Sequence:** MKLLFVAALFLVIMDVYA	
**Predicted Peptide(s):** IQE, YIRFamide (x3), YIRFG (x3), HQQLFPN, EYIPLD, NDLLDSEFLNDMNENLE	
**Prohormone Cloning Method:** Amplified from cDNA	
**Oligonucleotide Primer(s):** GAAACTTTTATTCGTTGCTGCAT(Forward), AAAAACACTCGAAATCATCGTC (Reverse)	
**Asexual Expression Pattern:** CG, VNC, pharynx	
**Genbank accession:** BK007014	

**Gene Name:** *Smed-secreted peptide prohormone-12*	**Abbreviation:** *Smed-spp-12*
**Prohormone Sequence:** MQLLLILMLTYTFYSVHEATPAMRSDKLDREDWYGPFKRNYMDFFGLNGDMQRFKKQQFFRNH RPEIEWN	

**Signal Sequence:** MQLLLILMLTYTFYSVH	
**Predicted Peptide(s):** EATPAMRSD§, LDREDWYGPF, NYMDFFGLNGDMQRF, QQFFRNHRPEIEWN, pQQFFRNHRPEIEWN.	
**Prohormone Cloning Method:** 3' RACE	
**Oligonucleotide Primer(s):** TGCAATTACTTCTAATACTTATGCTGA (OUTER), TGACGTATACCTTTTACTCCGTTC(INNER)	
**Asexual Expression Pattern:** CG, VNC, Cells anterior to CG, unidentified sub-epidermal cells, pharynx	
**Genbank accession:** BK007023	

**Gene Name:** *Smed-secreted peptide prohormone-13*	**Abbreviation:** *Smed-spp-13*
**Prohormone Sequence:** MKFTIYTVLTATLWIFLVQTEEAEIPQKDFLQNYDLIPNYEQRIEDSWRDPDRIRLMKKFRGLLGKRVHAKRFRGLLG	
**Signal Sequence:** MKFTIYTVLTATLWIFLVQTEEA	
**Predicted Peptide(s):** EIPQKDFLQNYDLIPNYEQRIEDSWRDPDRIRLM, FRGLLamide(x2), FRGLLG(x2), VHA	
**Prohormone Cloning Method:** 3' RACE	
**Oligonucleotide Primer(s):** ATGAAATTTACAATTTACACCGTTCTG (OUTER), CAATTTACACCGTTCTGACAGCAAC (INNER)	
**Asexual Expression Pattern:** CG, VNC, unidentified sub-epidermal cells, pharynx, semi-circular ring of cells anterior to CG, anterior margin	
**Genbank accession:** BK007029	

**Gene Name:** *Smed-secreted peptide prohormone-14*	**Abbreviation:** *Smed-spp-14*
**Prohormone Sequence:** MKLTYTLSIIYFVTFTLCNSYDYDTKISNKRPYNGKRNGLDIDDLFMGKRTYLGKRTYLGKRTYLGKRLDHSISRLLPQAFANTKYQLYRNQ	
**Signal Sequence:** MKLTYTLSIIYFVTFTLC	
**Predicted Peptide(s):** NSYDYDTKISN, PYNamide, PYNG, NGLDIDDLFMamide, NGLDIDDLFMG, TYLamide (x3), TYLG(x3), LDHSISRLLPQAFANTKYQLYRNQ	
**Prohormone Cloning Method:** 3' RACE	
**Oligonucleotide Primer(s):** ATGAAGCTGACTTATACTCTT (OUTER), AGCTGACTTATACTCTTAGTATCA (INNER)	
**Asexual Expression Pattern:** CG, VNC (single line of cells)	
**Genbank accession:** BK007030	

**Gene Name:** *Smed-secreted peptide prohormone-15*	**Abbreviation:** *Smed-spp-15*
**Prohormone Sequence:** MQFRFSKMTHVTLLITVGLFHVISGYPQTYELNQSDRGYYPILFDKRFDPIQFGKRFDPIQFGKRFDPIQFGKRFDPIQFGKRFDPIQFGKRFDPIQFGKRFDPIQFGKKFDPIQFGKRFDPIMFGR	
**Signal Sequence:** MQFRFSKMTHVTLLITVGLFHVISG	
**Predicted Peptide(s):** YPQTYELNQSDRGYYPILFD, FDPIQFamide (x8), FDPIQFG (x8), FDPIMFamide§, FDPIMFG	
**Prohormone Cloning Method:** Amplified from cDNA	
**Oligonucleotide Primer(s):** TGCAATTTAGATTTTCAAAGATGA (Forward), TCTGCCAAACATTATTGGATCA (Reverse)	
**Asexual Expression Pattern:** CG, VNC, unidentified sub-epidermal cells, pharynx	

**Genbank accession:** BK007013

**Gene Name:** *Smed-secreted peptide prohormone-16*	**Abbreviation:** *Smed-spp-16*
**Prohormone Sequence:** MKIYILCLSLFFLQQLSVTRSFPSSNEIYKRQFDPIMYGKLRQFYRRSSKIGKGQFDPTMYEKSIFA KRQFDPIMYKRQSNPYFLSDIRSIKRQFDPIMY	
**Signal Sequence:** MKIYILCLSLFFLQQLSVTRS	
**Predicted Peptide(s):** FPSSNEIY, QFDPIMYGKLRQFY, pQFDPIMYGKLRQFY, QFDPIMYamide[§], SS, IGKGQFDPTMYEKSIFA, QFDPIMY(x2), pQFDPIMY(x2), QSNPYFLSDIRSI, pQSNPYFLSDIRSI	
**Prohormone Cloning Method:** EST PL05004A1G02, Contig1470	
**Oligonucleotide Primer(s):** N/A	
**Asexual Expression Pattern:** CG, VNC, pharynx, unidentified sub-epidermal cells, anterior concentration of cells outside CG	
**Genbank accession:** BK007042	

**Gene Name:** *Smed-secreted peptide prohormone-17*	**Abbreviation:** *Smed-spp-17*
**Prohormone Sequence:** MNKVIVLLLFLFIAFSSETPFKRYIQDPDENDTLRDFYLQNRMENSKSKKIIDPMTYGTGFSNL	
**Signal Sequence:** MNKVIVLLLFLFIAFS	
**Predicted Peptide(s):** SETPF, YIQDPDENDTLRDFYLQNRMENSKS, IIDPMTYGTGFSNL	
**Prohormone Cloning Method:** 3' RACE	
**Oligonucleotide Primer(s):** AAATGAATAAAGTAATTGTTTTACTCC (Outer)	
**Asexual Expression Pattern:** CG, VNC, pharynx, unidentified sub-epidermal cells, unidentified cells between VNC in anterior half of animals, cells surrounding pharynx	
**Genbank accession:** GU295183	

**Gene Name:** *Smed-secreted peptide prohormone-18*	**Abbreviation:** *Smed-spp-18*
**Prohormone Sequence:** MKSGALTVVGFCLISYCMSAIPEDYEKRGYHFFRLKKSGDCVIPDVMKAMIETKIQNHEQLCAADK KFIEMISSV	
**Signal Sequence:** MKSGALTVVGFCLISYCMS	
**Predicted Peptide(s):** AIPEDYE, GYHFFRL, SGDCVIPDVMKAMIETKIQNHEQLCAAD, FIEMISSV	
**Prohormone Cloning Method:** 3' RACE	
**Oligonucleotide Primer(s):** ATGAAGAGCGGAGCATTAACT (Outer)	
**Asexual Expression Pattern:** CG, unidentified sub-epidermal cells	
**Genbank accession:** GU295184	

**Gene Name:** *Smed-secreted peptide prohormone-19*	**Abbreviation:** *Smed-spp-19*
**Prohormone Sequence:** MNGTVILCTLIVLLASFPSDGSDDLIVKRKHIGHQIFRLKRGYHFFRLRKDEKCLIPGELKSAIKNDLE SGSNLCGSDKSFLAELASLL	
**Signal Sequence:** MNGTVILCTLIVLLASFPSDG	
**Predicted Peptide(s):** SDDLIV, KHIGHQIFRL, GYHFFRL, DEKCLIPGELKSAIKNDLESGSNLCGSDKSFLAELASLL	
**Prohormone Cloning Method:** 3' RACE	

**Oligonucleotide Primer(s):** ATGAATGGAACGGTTATACTTTGC (Outer)
**Asexual Expression Pattern:** CG, VNC, unidentified sub-epidermal cells
**Genbank accession:** GU295186

Peptides confirmed by MS/MS sequencing are colored red and peptides detected by mass match are shaded. Peptides marked with § were identified with lower confidence, and peptides labeled with Φ are tentative, because their corresponding prohormone identification do not meet the criteria as described in experimental section.
