# Supplementary material for: Genome-Wide Analyses Reveal a Role for Peptide Hormones in Planarian Germline Development
Source: PLoS Biol. 2010 Oct 12;8(10):e1000509. doi: 10.1371/journal.pbio.1000509 (PMC2953531; doi:10.1371/journal.pbio.1000509)
Supplement: Table S6 — Changes in characterized and uncharacterized peptides following pc2(RNAi) treatment. (0.09 MB PDF) [file pbio.1000509.s011.pdf]

Table S6. Changes in characterized and uncharacterized peptides following *pc2(RNAi)* treatment

Mass	Prohormone	p-value	Ratio *pc2(RNAi)*/ Control*
1141.15	1020HH-2	0.0547	0.64
1415.07	EYE53-2	0.0168	0.70
1668.29	NPP-18	0.0294	1.92
1295.4	PPP-1	0.000371	0.53
1359.38	PPP-2	0.0055	0.51
1193.37	SPP-10	0.00208	0.63
1251.22	SPP-10/NPP-18	0.0168	1.69
1523.78	SPP-16	0.0153	0.72
1539.49	SPP-16	0.000371	0.42
1031.69	SPP-3	0.0438	2.13
1189.23	SPP-3	0.0547	0.50
1429.61	SPP-4	0.00194	0.53
2594.13	SPP-4	0.0122	0.57
1004.65	SPP-5	0.0153	2.27
2163.3	SPP-5	0.0108	0.72
1482.82	SPP-7	0.0153	0.44
1562.98	SPP-8	0.0341	0.72
1108.85	Unknown	0.0153	1.85
1280.9	Unknown	0.00261	0.46
1353.31	Unknown	0.035	0.75
1544.65	Unknown	0.00633	0.72
1590.67	Unknown	0.000862	0.49
1912.48	Unknown	0.0153	0.30
2086.58	Unknown	0.014	0.51
2173.44	Unknown	0.0372	3.33
2461.78	Unknown	0.00436	0.45
2483.73	Unknown	0.014	0.64
2499.86	Unknown	0.014	0.56
2715.07	Unknown	0.0295	2.13
2726.84	Unknown	0.0168	0.12
2823.48	Unknown	0.0153	0.23
2829.58	Unknown	0.0102	0.05
2851.62	Unknown	0.00783	0.13
2855.71	Unknown	0.044	0.44
2901.57	Unknown	0.0168	0.20
2928.15	Unknown	0.0294	1.33
2960.76	Unknown	0.0249	3.33
2980.42	Unknown	0.0108	0.28
3008.79	Unknown	0.0249	3.23
3034.68	Unknown	0.0523	3.33
3047.79	Unknown	0.0482	2.27
3095.96	Unknown	0.035	2.50
4333	Unknown	0.00633	0.45
4999.7	Unknown	0.0233	0.44
5013.88	Unknown	0.0361	0.32
5884.18	Unknown	0.0514	0.56
5892.15	Unknown	0.035	0.49

*The abundance of some peptides was increased following *Smed-pc2(RNAi)*; whether this reflects a feedback mechanism for regulating peptide levels or an altered threshold of detection for certain peptides caused by a global reduction in neuropeptide levels remains to be determined.
