# Supplementary material for: Genome-Wide Analyses Reveal a Role for Peptide Hormones in Planarian Germline Development
Source: PLoS Biol. 2010 Oct 12;8(10):e1000509. doi: 10.1371/journal.pbio.1000509 (PMC2953531; doi:10.1371/journal.pbio.1000509)
Supplement: Table S7 — Prohormone genes from Schistosoma that encode peptides related to peptides from S. mediterranea . (0.10 MB PDF) [file pbio.1000509.s012.pdf]

Table S7. Prohormone genes from *Schistosoma* that encode peptides related to peptides from *S. mediterranea*

**Gene Name:** *S. mansoni npp-26*	**Abbreviation:** *Sma-npp-26*
**Prohormone Sequence:** MKIYKFDNKMIIEQLFYCLLVFITFSSIVNAIPVHELNYVNHKTNDPIFGRRYEGRYPTDGNSRFIESE YSNFYPMAFKRTLFNPILFKRNFDPILFKRSYFDPIIYKRSYFDPILFKRNEDRQFEKREHFDPIIY	
**Signal Sequence:** MKIYKFDNKMIIEQLFYCLLVFITFSSIVNA	
**Predicted Peptide(s):** IPVHELNYVNHKTNDPIFG, IPVHELNYVNHKTNDPIFamide, YEGRYPTDGNSRFIESEYSNFYPMAF, TLFNPILF, NFDPILF, SYFDPIIY, SYFDPILF, NEDRQFE, EHFDPIIY	
**Accession number:** Smp_071050	

**Gene Name:** *S. japonicum npp-26*	**Abbreviation:** *Sja-npp-26*
**Prohormone Sequence:** MFIEQCFYLLLGLLIFSSTVNAVPVREPNYENHGILGNRLGKHFERQYFVESDYPDKRSY FDPIAFKRTYFDPIAFKRNFDRILFKRNFDPILFKRSYFDPIAFKRNADHQFDKREYFDPIIY	
**Signal Sequence:** MFIEQCFYLLLGLLIFSSTVNA	
**Predicted Peptide(s):** VPVREPNYENHGILGNRLG, VPVREPNYENHGILGNRLamide, HFERQYFVESDYPD, TYFDPIAF, NFDRILF, SYFDPIAF, NADHQFD, EYFDPIIY	
**Accession number:** SJCHGC06632	

**Gene Name:** *S. mansoni npp-27*	**Abbreviation:** *Sma-npp-27*
**Prohormone Sequence:** MVTSVLIFVIVSFCWYNPVKSQFPPNFRTFDLEDDYPEIPGRYSPSMHKDHTTHFNYKDRFRWSK RVPPYITGGIRYR	
**Signal Sequence:** MVTSVLIFVIVSFCWYNPVKS	
**Predicted Peptide(s):** QFPPNFRTFDLEDDYPEIPGRYSPSMHKDHTTHFNYKDRFRWS, VPPYITGGIRY	
**Accession number:** Smp_134550	

**Gene Name:** *S. japonicum npp-27*	**Abbreviation:** *Sja-npp-27*
**Prohormone Sequence:** MNYSMSTIVLLILIVSLCCYVPVKSQLPSTYQLLDTDDEYLDPSGQYSSSTIQHKLHIPHYNYKDKF RWNKRVPPYITGGIRY	
**Signal Sequence:** MNYSMSTIVLLILIVSLCCYVPVKS	
**Predicted Peptide(s):** QLPSTYQLLDTDDEYLDPSGQYSSSTIQHKLHIPHYNYKDKFRWN, VPPYITGGIRY	
**Accession number:** SJCHGC09751	

**Gene Name:** *S. mansoni npp-23*	**Abbreviation:** *Sma-npp-23*
**Prohormone Sequence:** PHKPFYDPLYEYSDYINQERTPNKRYIRFGKRGADDVMRYDGIPALPRHKPYSLYDLLKERQ	
**Signal Sequence:** N/A partial length sequence, see *Sja-spp-11*	
**Predicted Peptide(s):** YIRFG, YIRFamide, GADDVMRYDGIPALPRHKPYSLYDLLKERQ	
**Accession number:** Smp_188580	

**Gene Name:** *S. japonicum npp-23*	**Abbreviation:** *Sja-npp-23*
**Prohormone Sequence:** MKTNISNLFNILLLTILGLLLVNVNSSPDEQNSYKYVYEPLYEYSDNINQETFPAKRYIRFGKRGGD DIMHYGGHPTLARHKTYSIYDLLKEKHYN	
**Signal Sequence:** MKTNISNLFNILLLTILGLLLVNVNS	
**Predicted Peptide(s):** SPDEQNSYKYVYEPLYEYSDNINQETFPA, YIRFG, YIRFamide, GGDDIMHYGGHPTLARHKTYSIYDLLKEKHYN	
**Accession number:** CAX72971	

**Gene Name:** *S. mansoni npp-28*	**Abbreviation:** *Sma-npp-28*
**Prohormone Sequence:** MKHTMVNMISMFTLMVLFTVQLQSIKINAESTDDMNFEKKAYHFFRLKKSDRCLRIPYSIKQ MILREPSIVCPEDREFLGL	
**Signal Sequence:** MKHTMVNMISMFTLMVLFTVQLQS	
**Predicted Peptide(s):** IKINAESTDDMNFE, AYHFFRL, SDRCL, IPYSIKQMILREPSIVCPEDREFLGL	
**Accession number: Smp_043650**	

**Gene Name:** *S. japonicum npp-28*	**Abbreviation:** *Sja-npp-28*
**Prohormone Sequence:** MKHTLINIIYMFTLVILIAIQLETMQTSAERTNDGDFDKRAYQFFRLKKSDGCLRIPESVKRVI LTEPYILCPEDREFLGL	
**Signal Sequence:** MKHTLINIIYMFTLVILIAIQLETMQTSA	
**Predicted Peptide(s):** ERTNDGDFD, AYQFFRL, SDGCLRIPESV, VILTEPYILCPEDREFLGL	
**Accession number:** CX860443	
